# Hidden regulation of herpes simplex virus 1 pre-mRNA splicing and polyadenylation by virally encoded immediate early gene ICP27

**DOI:** 10.1371/journal.ppat.1007884

**Published:** 2019-06-17

**Authors:** Shuang Tang, Amita Patel, Philip R. Krause

**Affiliations:** Division of Viral Products, Office of Vaccines Research and Review, Center for Biologics Evaluation and Research, Food and Drug Administration, Silver Spring, Maryland, United States of America; University of California, Irvine, UNITED STATES

## Abstract

In contrast to human cells, very few HSV-1 genes are known to be spliced, although the same pre-mRNA processing machinery is shared. Here, through global analysis of splice junctions in cells infected with HSV-1 and an HSV-1 mutant virus with deletion of infectious cell culture protein 27 (ICP27), one of two viral immediate early (IE) genes essential for viral replication, we identify hundreds of novel alternative splice junctions mapping to both previously known HSV-1 spliced genes and previously unknown spliced genes, the majority of which alter the coding potential of viral genes. Quantitative and qualitative splicing efficiency analysis of these novel alternatively spliced genes based on RNA-Seq and RT-PCR reveals that splicing at these novel splice sites is efficient only when ICP27 is absent; while in wildtype HSV-1 infected cells, the splicing of these novel splice junctions is largely silenced in a gene/sequence specific manner, suggesting that ICP27 not only promotes accumulation of ICP27 targeted transcripts but also ensures correctness of the functional coding sequences through inhibition of alternative splicing. Furthermore, ICP27 toggles expression of *ICP34*.*5*, the major viral neurovirulence factor, through inhibition of splicing and activation of a proximal polyadenylation signal (PAS) in the newly identified intron, revealing a novel regulatory mechanism for expression of a viral gene. Thus, through the viral IE protein ICP27, HSV-1 co-opts both splicing and polyadenylation machinery to achieve optimal viral gene expression during lytic infection. On the other hand, during latent infection when ICP27 is absent, HSV-1 likely takes advantages of host splicing machinery to restrict expression of randomly activated antigenic viral genes to achieve immune evasion.

## Author summary

Little is known regarding to how HSV, a large DNA virus and known to contain very few spliced genes, escapes host pre-mRNA splicing machinery. Here, by establishing a high throughput splice junction identification platform and quantitative analysis method to assess splicing efficiency based on high throughput data, we find that HSV-1 encodes hundreds of previously unknown alternative splice junctions; however, splicing of these novel spliced genes is largely silenced in wild-type HSV-1 infected cells, explaining why only very few spliced genes have been previously identified in HSV-1. Moreover, ICP27 is required for splicing inhibition and 3’ end formation of *ICP34*.*5*, the major viral neurovirulence factor and also the major target of latently expressed viral miRNAs. These findings not only fundamentally change the view of HSV gene structure, but also reveal a mechanism by which HSV employs host splicing and polyadenylation machineries to achieve optimal gene expression during acute infection and may also contribute to immune evasion during latency when ICP27 is not expressed.

## Introduction

HSV-1 and HSV-2, two closely related human herpes viruses, establish lifelong incurable latency in and reactivate preferentially from trigeminal ganglia and dorsal root ganglia to cause orofacial and genital herpes, respectively. Although infections are usually mild, these viruses can cause severe disease including encephalitis and neonatal herpes. During latency in terminally differentiated neurons, expression of viral genes is repressed, except for the latency-associated transcript (*LAT*) and latency-associated miRNAs [1–3]. During acute infection, herpesvirus genes are expressed in a coordinated temporal cascade characterized by three kinetic classes, immediate-early (IE or α), early (β), and late. Late genes are further divided into two subclasses: leaky-late (γ1) genes that are expressed at very low levels at early times after infection and are dramatically upregulated at late times as a result of the increased number of genomes present after DNA replication, and true late genes (γ2) that are expressed exclusively after and are dependent upon viral DNA replication. HSV infected cell culture polypeptide 27 (*ICP27*), along with *ICP4*, are the only two IE genes essential for virus replication [1, 4]. *ICP27*, highly conserved between HSV-1 and HSV-2, is also the only one of the five HSV-1 IE genes that has clear homologs in all characterized mammalian herpesviruses (8). *ICP27* is known to be required for efficient expression of some viral DNA replication-related early genes and late viral genes as well as for virus growth [5, 6]. ICP27 plays a role in transcriptional regulation through association with the C-terminal domain of RNA polymerase II [7, 8] and interacts with viral transactivating proteins encoded by immediate early genes including *ICP4* and *ICP0* [9–11]. ICP27 forms homo-dimers [12, 13], interacts with U1 snRNP through its C-terminal domain, and colocalizes with U1 and U2 snRNPs [14, 15]. ICP27 also interacts with splicing factors such as SRSF1, SRSF2, SRSF3, and SRSF7 through its C-terminal domain, and SR protein kinase 1 (SRPK1) through its N-terminal RGG RNA-binding domain [16–19]. Recently, ICP27 was shown to inhibit splicing of certain introns and promote use of alternative 5′splice sites (ss) in a small percentage of cellular genes in a sequence specific manner [20]. ICP27 also promotes co-transcriptional cellular pre-mRNA 3’ end formation using cryptic polyadenylation signals (PAS) in proximal introns, generating hundreds of novel, intronless GC-rich cellular transcripts that resemble HSV genes [20].

Although HSV-1 pre-mRNAs are transcribed in the nucleus by host transcription and RNA processing machineries, only 6 genes out of at least 84 genes, including 3 out of the 5 immediate early genes (*ICP0*, *ICP22* and *ICP47*), a latently expressed gene (the latency associate transcript, or *LAT*) and two late genes (*UL15* and *gC*), have until now been identified as spliced genes [1, 21]. Recently, a few novel splice isoforms including two antisense transcripts, *UL41-42C* (transcript initiated antisense to *UL41*) and *AST-2* (transcript antisense to *UL36*), as well as *UL49sp* (splice site flanked by a unusual GC-AG intron) were identified using high throughput long-read sequencing in HSV-1 infected Vero cells [22].

Since ICP27-targeted host genes contain high GC content and cytosine-rich sequences, resembling HSV genes [20], we hypothesized that ICP27 likely co-evolved with the GC-rich viral genome and may have additional unknown viral targets. In this report, we further investigate the role of ICP27 in regulating pre-mRNA processing of viral genes. In addition to discovery of novel alternative splice sites for known viral spliced genes, we identify 22 novel viral spliced genes, most of which are tightly controlled by ICP27. Furthermore, we find that ICP27 tightly regulates expression of monocistronic ICP34.5 mRNA by inhibiting splicing and activating a PAS in the newly identified proximal intron, which represents a novel regulation mechanism for viral gene expression.

## Results

### Confirmation of ICP27’s essential role in regulating viral gene expression

**Fig 1:**
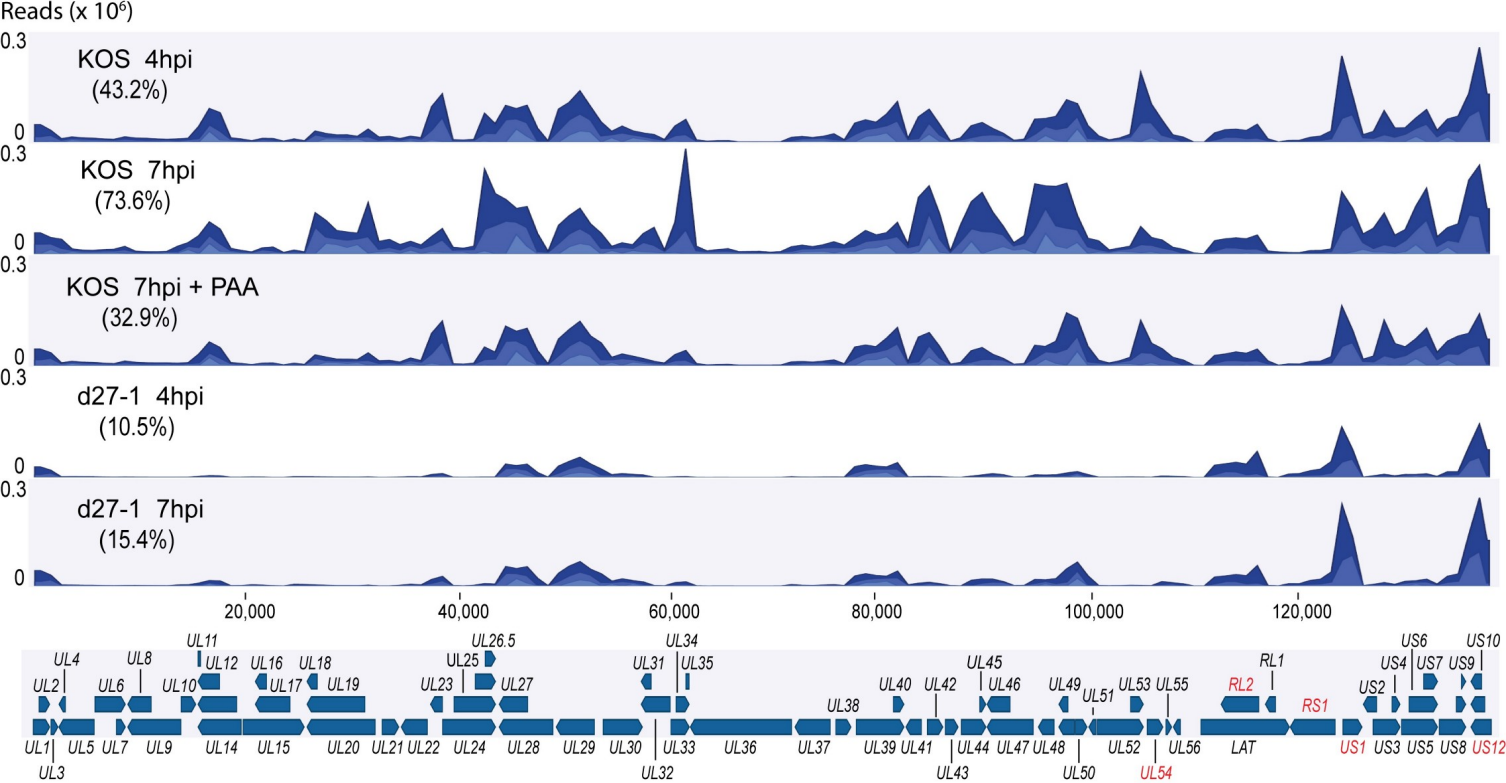
ICP27 deletion reduces expression of non-α viral mRNAs. RNA sequences from HEK-293 cells infected with an HSV-1 ICP27 deletion mutant (d27-1) or its wild-type parental strain (KOS) in the presence of the viral polymerase inhibitor phosphonoacetic acid (PAA) or not at 4 and 7 hpi were aligned to the HSV-1 genome (after removal of terminal repeat sequences, which are represented by internal repeats) and graphed as number of viral reads at each genome location. Genome positions of HSV genes relative to the trimmed genome are shown under the graph. Expression of HSV-1 IE genes including *RL2* (*ICP0*), *RS1* (*ICP4*), *US1* (*ICP22*) and *US12* (*ICP47*) labelled in red was similar between KOS or d27-1 infected cells. IE gene UL54 (ICP27) is not detectable in d27-1 infected cells since the coding region of UL54 was deleted in d27-1.

HSV infects most cell types *in vitro* including human kidney HEK-293 cells, which have been widely used in previous pre-mRNA splicing and polyadenylation related studies. To further understand *ICP27*’s role in viral pre-mRNA processing in a way aligning directly to previous findings [20], we performed RNA-Seq using poly(A)-enriched RNA purified from HEK-293 cells infected with wild-type HSV-1 strain KOS, or an *ICP27* deletion mutant (d27-1) in the presence or absence of the viral polymerase inhibitor phosphonoacetic acid (PAA) at 4 hours post infection (hpi) or 7 hpi. The RNA-Seq data were analyzed using CLC genomic Workbench and the HSV-1 consensus sequence without the terminal repeat sequences was used as the reference (Fig 1). In KOS infected HEK-293 cells, reads mapping to the HSV-1 consensus sequence increased from approximately 43.2% at 4 hpi to 73.6% at 7 hpi, a result similar to that previously reported in infected MRC-5 human fibroblast cells [23]. However, deletion of *ICP27* reduced reads mapping to the HSV-1 genome to approximately 10.5% at 4 hpi and 15.4% at 7 hpi, a reduction much greater than that induced by the viral polymerase inhibitor phosphonoacetic acid (PAA), which markedly reduced transcription only of γ2 genes (the subset of viral DNA replication-dependent γ genes). Reads mapping to the IE gene *UL54* (*ICP27*) are not detectable in d27-1 infected cells since the coding region of *ICP27* was deleted in d27-1. Deletion of *ICP27* does not appreciably affect other α viral gene expression, but reduced non-α (i.e., β and γ) viral gene expression, confirming *ICP27*’s essential role in promoting β and γ viral gene expression.

### Confirmation of HSV-1 and ICP27’s role in mediating aberrant cellular pre-mRNA processing

**Fig 2:**
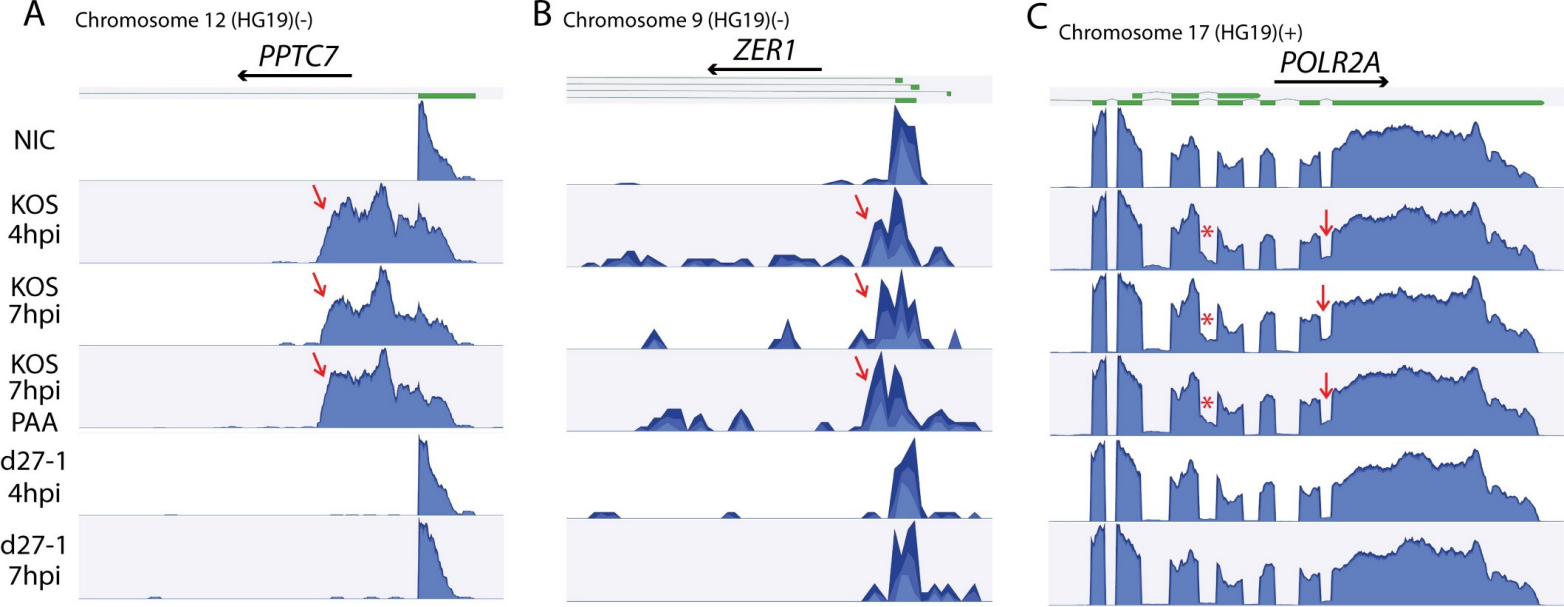
HSV-1 ICP27 causes aberrant pre-mRNA processing of cellular transcripts in infected cells in a gene/sequence specific manner. RNA-seq data from wild-type HSV or ICP27 deletion mutant infected HEK- HEK-293 cells were aligned to the human genome HG19 reference sequence using CLC Genomics Workbench. Representative genes for which co-transcriptional pre-mRNA processing is modulated in ICP27 expression plasmid-transfected cells [20] are provided as examples and shown in (**A**) Use of cryptic PAS in intron 1 of *PPTC7* (negative strand), (**B**) Use of cryptic 5’ss in intron 1 of *ZER1* (negative strand), and (**C**) Last intron retention in *POLR2A* (positive strand), supporting previous findings in HSV-2 ICP27 transfected cells [20]. Retention of intron 24 of *POLR2A* (labelled as *) was not previously observed in HSV-2 ICP27 expression plasmid transfected cells. Previously described transcript isoforms (thick green lines on top of the graphs denote exons) are shown above each graph. Low level read counts mapping downstream of the cryptic 5’ss of *ZER1* suggest that there is likely a low level of intron retention coupled with aberrant use of the cryptic 5’ss. Arrows indicate significant differences in intronic read counts in KOS vs d27-1 infected cells. NIC: noninfection control.

The RNA-Seq data were also analyzed using CLC Genomics Workbench and the human genome consensus sequence (HG19) as the reference. Aberrant mRNA processing of ICP27-targeted cellular genes, previously identified as a result of ectopic HSV-2 ICP27 expression [20], was also observed in HSV-1 infected cells (with or without PAA), but not in d27-1 infected cells, confirming HSV-1 and ICP27’s role in mediating aberrant pre-mRNA processing in infected cells. The three previously described types of ICP27 mediated aberrant pre-mRNA processing (aberrant polyadenylation, aberrant use of 5’ss and intron retention) are apparent in three representative genes, *PPTC7*, *ZER1* and *POLR2A*, respectively (Fig 2). The RNA-Seq results for these three representative genes in KOS and d27-1 infected cells are consistent with RT-PCR and Northern blot results [20]. HSV-1 thus mediates aberrant pre-mRNA processing in a manner similar to ectopic expression of ICP27 alone. Expression of ICP27 in the context of viral infection appears to induce additional intron retention in *POLR2A* that was not observed with ectopic expression of HSV-2 ICP27 in transfected cells, suggesting either subtle differences between HSV-1 and HSV-2 ICP27 or that virus-produced ICP27 more efficiently inhibits splicing than does ectopic expression in transfected cells.

### ICP27 does not significantly influence splicing of viral IE spliced genes

**Fig 3:**
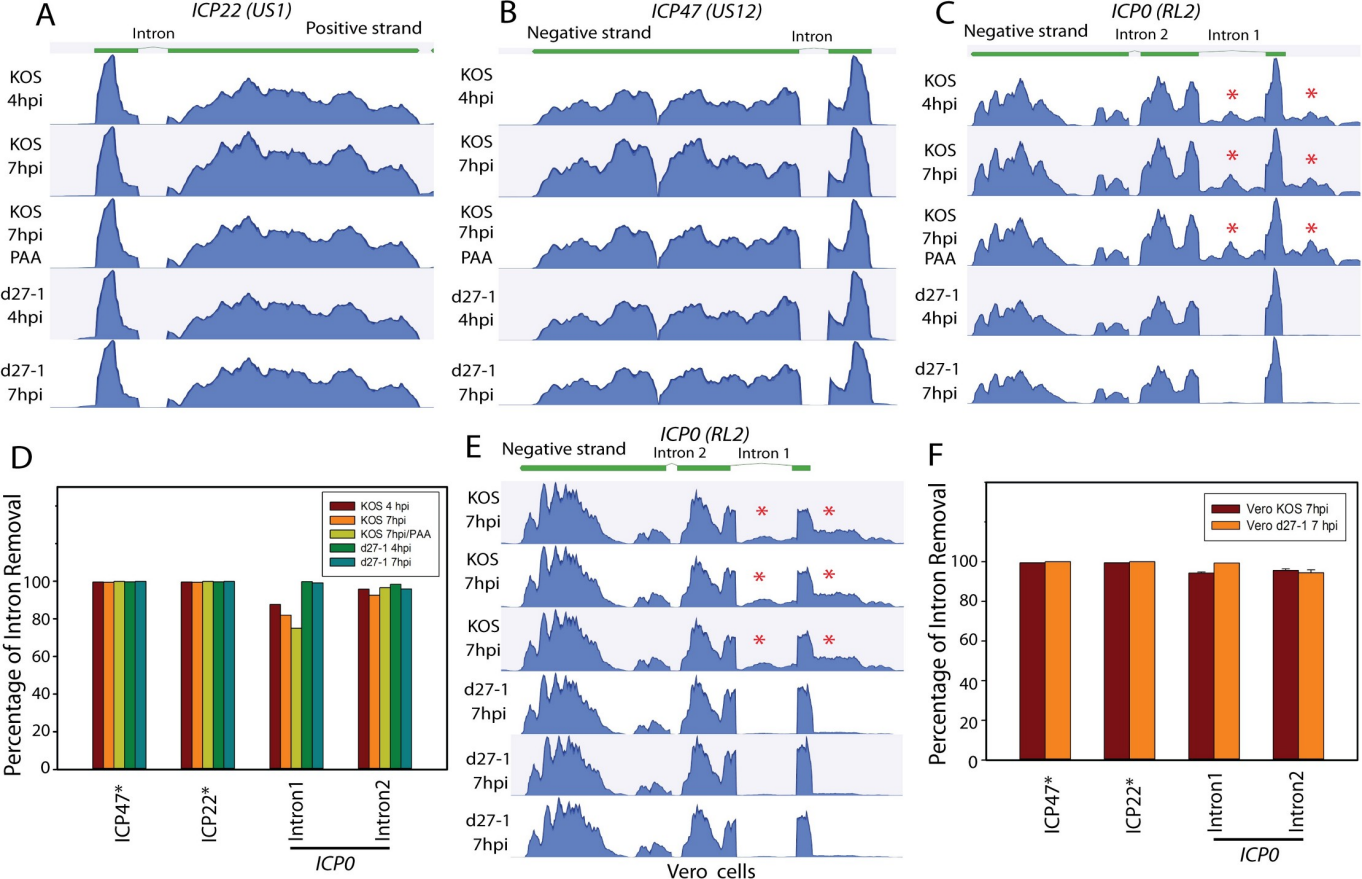
ICP27 does not appear to influence pre-mRNA splicing of IE genes. RNA-seq data from wildtype HSV or *ICP27* deletion mutant infected HEK- HEK-293 cells were aligned to the HSV-1 genome using CLC Genomics Workbench. Previously described non ICP27-dependent transcript isoforms (thick green lines above the graphs denote exons) are shown on top of the graph. Pre-mRNA splicing of (**A**) *ICP22* (positive strand), (**B**) *ICP47* (negative strand), and (**C**) *ICP0* (negative strand) was efficient in both KOS and d27-1 infected cells. Apparent retention of *ICP0* intron 1 in KOS-infected cells was accompanied by increased reads mapping upstream of the *ICP0* transcription initiation site. * indicates difference in read count mapping to ICP0 intron 1 and sequences upstream of the *ICP0* transcription initiation site between KOS and d27-1 infected cells. (**D**) Impact of ICP27 on splicing efficiency of the spliced IE transcripts in infected HEK-293 cells was quantitatively analyzed by mapping the high throughput sequencing data to reference sequences including exon-exon junction and exon-intron junction sequences. (**E**) RNA-seq data from wildtype HSV or *ICP27* deletion mutant infected Vero cells were analyzed for the *ICP0* locus. * indicates difference in read count mapped to ICP0 intron 1 and sequences upstream of the *ICP0* transcription initiation site between KOS and d27-1 infected cells. (**F**) Mean (and standard deviation) percentage of intron removal showing ICP27 impact on splicing efficiency of the spliced IE transcripts in infected Vero cells. based on the. Slightly less severe intron retention for ICP0 intron 2 was observed in KOS infected Vero cells vs. HEK-293 cells.

We used CLC Genomics Workbench to further analyze the RNA-Seq data to view the splicing pattern of known viral spliced genes (as presented in Fig 1) at a resolution of single genes. Expression of ICP27 does not appear to influence pre-mRNA splicing of *ICP47*, *ICP22*, or *ICP0* intron 2 (Fig 3A–3D). Slight retention of *ICP0* intron 1 was observed in HSV-1 strain KOS-infected cells; however, retention of ICP0 intron 1 was accompanied by increased reads mapping upstream of the *ICP0* transcription initiation site (Fig 3C). Because this approach may not detect splicing patterns of other known spliced genes due to much lower expression levels or to complex transcription patterns, the high throughput data were also mapped to a 44-bp reference unspliced sequence containing 22 bp of sequence from each exon and 22 bp sequence from the adjacent intron, and a 44-bp reference spliced sequence containing 22 bp sequence from each of the two exons expected to be joined following splicing. Each result was manually examined to confirm the results. The 44-bp length of these reference sequences detected splices in known spliced genes including ICP47, ICP0, UL15 and gC. The percentage of intron removal was thus calculated for individual genes in each of the five RNA-Seq data sets based on the reads mapping to exon-exon junction sequence relative to total reads mapped to both exon-exon junction and exon-intron junction sequences. The quantitative data support the graphical results in Fig 3A, 3B and 3C. ICP27 does not significantly influence the splicing of IE genes, except for intron 1 of ICP0 (Fig 3D). The splicing efficiency for ICP0 intron 1 appears to increase from approximately 85% in KOS-infected cells to over 99% in d27-1 infected cells. Increase of *ICP0* intron 1 retention is coupled with an increase in reads mapping to sequences upstream of the *ICP0* transcription initiation site. Co-transcriptional splicing of the first intron in a gene can be greatly enhanced by the RNA capping machinery and a large distance from a cap-proximal 5′ss to the RNA 5′ cap may reduce the chance of this splice site being recognized by cellular splicing machinery [24–26]. Taken together with the mapping results in Fig 3C, this implies that the observed retention of ICP0 intron 1 in KOS infected cells is likely in readthrough transcripts from upstream alternative promoters, for which splicing of ICP0 intron 1 is likely less efficient due to the increased distance between the 5’ cap and the 5’ss of ICP0 intron 1. The actual impact of ICP27 on the splicing of monocistronic ICP0 intron 1 is thus minimal, consistent with previous observations [27].

To confirm ICP27’s role in viral gene expression and obtain more precise quantitative information, we also performed RNA-Seq using Vero cells (monkey kidney cells that have been widely used in HSV studies) infected with KOS or d27-1 (S1 Fig). The infection was performed in triplicate in 6 well plates and the poly(A) selected RNA samples were prepared at 7 hpi. In this RNA-Seq data, the impact of ICP27 on overall viral gene expression was similar to the results obtained in infected HEK-293 cells as shown in Fig 1. The mean relative splicing efficiency and standard deviation were calculated based on the mapping results as described above. Retention of ICP0 intron 1, although less severe than in infected HEK-293 cells (Fig 3C), was also coupled with an increase of reads mapping to the sequences upstream of the *ICP0* transcription initiation site (Fig 3E and 3F). Thus, ICP27 does not appear to inhibit splicing of IE genes.

### Mapping viral splice junctions using RNA-Seq data obtained from d27-1 and wild-type HSV-1 infected cells

To understand ICP27’s role in global viral pre-mRNA processing, we further mapped potential viral splice junctions using MapSplice 2, software that identifies potential splice junctions relative to a reference genome without relying on sequence annotations (31), with the default parameter settings and the RNA-Seq data for d27-1 and KOS infected HEK-293 cells (7 hpi) presented in Figs 1, 2 and 3. Possible splice junctions were detected relative to the HSV-1 reference sequence (raw data are presented in S1 and S2 Tables). Most newly identified introns possess canonical splice junctions flanked by GT(GU) and AG. Although the total viral read counts in KOS infected cells are nearly 5-fold more than d27-1 infected cells (Fig 1), the total read counts of the splice junctions mapping to the viral genome were similar between KOS and d27-1 infected cells (148,652 for KOS infected cells, and 143,990 for d27-1 infected cells). A total of 1940 and 450 splice junctions mapping to the HSV-1 genome were identified in KOS infected cells and d27-1 infected cells, respectively. KOS infected cells contained significantly more rare splice junctions (reads ≤ 2) than did d27-1 infected cells (85% vs 44%), suggesting that splicing of these predicted and known viral genes may be generally inhibited in wild-type HSV-1 infected cells as compared with ICP27 deletion mutant virus infected cells.

We next mapped the predicted splice junctions listed in S1 and S2 Tables to the viral genome. Ten (10) out of eleven (11) previously identified splice junctions in six known HSV-1 spliced genes, including three IE genes (*ICP0*, *ICP47* and *ICP22*), two late genes (*UL15* and *gC*) and one latent gene (*LAT*), and two recently identified spliced transcripts (*UL41-42C* and *AST-2*) [28], are also identified by MapSplice 2 (Tables 1 and S1 and S2). Only very few reads (<10) were identified for splicing of *AST-2* intron 2 and no read was obtained for splicing of *AST-2* intron 1 or *UL41-42C* intron 3. No read was obtained for splicing of *UL49sp*, a newly identified spliced transcript with a 74 bp intron flanked by GC-AG [22]. Total reads for these known splice junctions identified in KOS and d27-1 infected cells accounted for approximately 92.3% and 89.5% of total viral splice junctions detected, respectively.

### Known HSV-1 spliced genes, including *LAT*, *ICP0* and *UL15*, contain novel alternative splice sites (ss)

In addition to the known spliced junctions identified, a total of 13 novel alternative splice junctions for transcripts of 7 of the known spliced HSV-1 transcripts, including *LAT*, *ICP0*, *UL15*, *ICP22*, *ICP47*, *gC* and *UL41-42C* were identified and the reads for these novel alternative splice junctions accounted for approximately 2.2% of the total junctions identified (Tables 1 and S1). We confirmed the novel splice junctions mapping to *LAT*, *ICP0* and *UL15* by RT-PCR and sequencing.

**Table 1:** List of splice sites of previously known spliced genes and transcripts identified in the *ICP27* mutant virus and wild-type HSV-1 (KOS) infected cell.

	Viral Genes	5’ Splice Junction	3’ Splice Junction	Reads (d27-1)	Reads (KOS)	Strand	Size of Intron (nt)	Classes	Verified by Sequencing
**Known** **spliced genes**	***LAT***	***119352***	***121311***	4	429	+	1959	Latent; γ²	Fig 4
		119628^**a**^	121311	0	56	+	1683		Fig 4
	***UL15***	***29990***	***33581***	3573	2098	+	3591	γ, essential	Fig 4
		28566	33581	206	3	+	5015		Fig 4
		33431	33581	169	283	+	150		Fig 4
		27364	33581	94	26	+	6217		Fig 4
		28566	29957	130	6	+	1391		Fig 4
		29033	29957	87	2	+	924		Fig 4
	***ICP0*** **(intron 1)**	***123950***	***123186***	16873	28617	-	764	α, nonessential at high multiplicities	Fig 4
		123402	123186	5	1045	-	216		Fig 4
	***ICP0*** **(intron 2)**	***122520***	***122380***	2903	4677	-	140		Fig 4
		122520	122377	1868	3475	-	143		Fig 4
	*ICP22* (*US1*)*	***132232***	***132400***	30190	30732	+	168	α, nonessential	Fig 8
		132213	132400	171	102	+	187		?
	*ICP47* (*US12*)*	***145457***	***145625***	75081	66113	-	168	α, nonessential	Fig 8
		145457	145644	354	202	-	187		?
	***gC* (*UL44*)**	***97641***	***97867***	173	215	+	226	γ², nonessential	Ref [21]
		96231^a^	96364	0	57	+	133		?
**Spliced transcripts recently identified using** **PAC-Bio sequencing**	***UL41-42C (Intron 1)***	***91042***	***91334***	28	870	+	292	Unknown	Ref [69]
		90999	91334	4	369	+	335		?
		91042	91439	1	91	+	397		?
	***UL41-42C (Intron 2)***	***91472***	***92456***	6	3387	+	984		Ref [69]
		***91472***	***97867***	0	5	+	6395		Ref [69]
	***AST-2* Intron 2**	***79818***	***80026***	8	5	+	208	Unknown	Ref [69]

Splice junctions were identified by MapSplice 2 using RNA-Seq data from KOS and d27-1 infected cells at 7 hpi (S1 and S2 Tables). Previously identified splice junctions are labeled in bold italics. For previously reported splice junctions, only the splice junctions with read counts ≥ 1 from either KOS or d27-1 infected cells were included in the table. For previously unreported splice junctions, only the splice junctions with read counts ≥ 30 from either KOS or d27-1 infected cells are listed. ^a^ intron is flanked by GC-AG. * ICP47 and ICP22 share the same splice sites in their 5’ UTR.? denotes that the isoform has not been confirmed by RT-PCR and sequencing. Classification of related viral genes is noted accordingly [1]. KOS complete genome [JQ673480] was used as the reference genome. ORF: Open-reading frame. Aa: amino acid. *UL49sp* [22] that appears to use GC-AG splice sites was not detected in this study.

**Fig 4:**
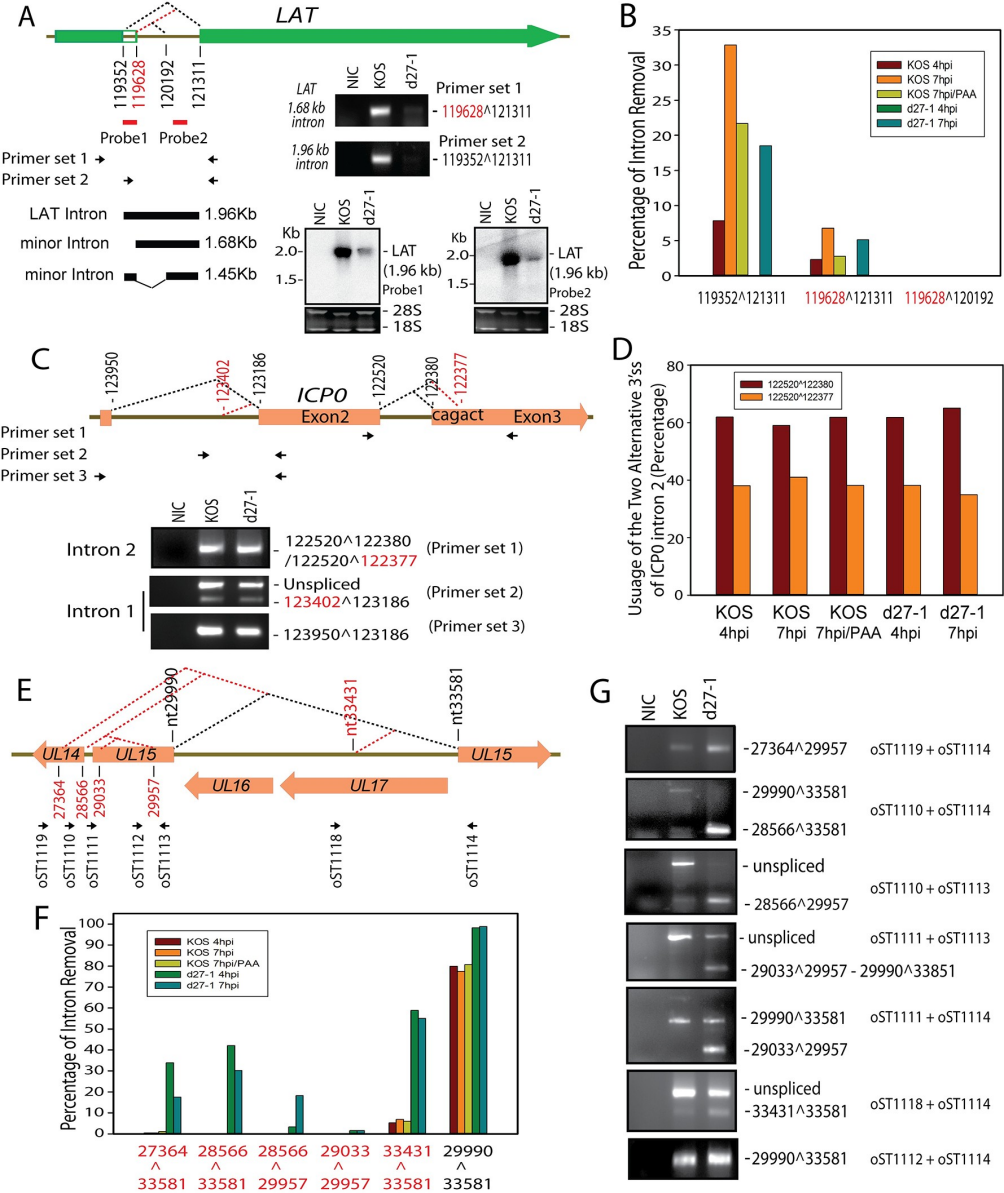
Confirmation of novel alternative splice sites in previously described spliced viral genes. (**A**) A diagram of *LAT* gene structure relative to *LAT* introns is shown at the top. A a novel splice junction mapped to *LAT*, flanked by a 1.68 kb “GC-AG” intron is labeled in red. The primary *LAT* is transcribed antisense to *ICP0*, *ICP34*.*5* and *ICP4*. Relative locations of primers and probes used are labeled under the diagram. The novel splice junction flanked by the 1.68 kb intron and *LAT* splice junction flanked by the 1.96 kb intron were confirmed by RT-PCR and subsequent sequencing using the cDNAs prepared from HEK-293 cells infected with KOS cells (7 hpi) (Middle of the panel). A previously reported splice junction within the LAT intron (119628^120192) was not confirmed by RT-PCR in the same cDNA sample. Northern blot using two different probes in Vero cells infected with KOS or d27-1 (16 hpi) detected the 1.96 kb *LAT* intron; however, the 1.45 kb intron and the predicted 1.68 kb intron were under the detection limit. (**B**) Relative splicing efficiency of the three *LAT* splicing variants were quantitively analyzed by mapping the RNA-Seq data obtained from infected HEK-293 cells to reference sequences including exon-exon and exon-intron junction sequences. No reads mapping to LAT exon-exon and exon-intron junctions were identified for d27-1 infected HEK-293 cells at 4 hpi. Reads mapping to the previously reported splice junction 119628^120192 were not identified in any samples. (**C**) Confirmation of alternative splice junctions for *ICP0*. Diagram of novel splice sites (labeled in red) mapping to *ICP0* intron 1 and intron 2 (top of the panel). The novel splice variants for ICP0 intron 1 and intron 2 were confirmed by RT-PCR and subsequent sequencing using the same cDNAs prepared from HEK-293 cells infected with KOS cells (7 hpi) (Bottom of the panel). For *ICP0* intron 2, the ratio of splice isoforms 122520^122380 (based on the NCBI HSV-1 reference sequence JQ673480) vs. 122520^122377 was approximately 1: 3 in a total of 12 clones sequenced from the PCR band obtained in d27-1 infected cells. Quantitative analysis of usage of the novel 3’ss of *ICP0* intron 2 using the RNA-Seq data obtained from infected HEK-293 cells and CLC Genomic Workbench is shown in Panel (**D**). Usage of the novel ICP0 3’ss appear not to be affected by the presence or absence of ICP27. (**E**) Diagram showing novel splice junctions in the *UL15* region. Novel splice sites are labeled in red. *UL16* and *UL17* are transcribed antisense to the *UL15* intron (29990^33581). Arrows indicate the relative location of primers used in Panel (G). Quantitative analysis of splicing efficiency of the splice junctions mapping to the *UL15* region using the HEK-293 RNA-Seq is shown in Panel (**F**). Presence of ICP27 significantly reduces the splicing efficiency at these novel splice sites. (**G**) Confirmation of novel alternative splice sites mapping to the UL15 region using RT-PCR in HEK-293 cells infected with HSV-1 KOS strain (WT) or d27-1 using primer sets illustrated in panel (E). RT-PCR bands representing novel splicing sites were further confirmed by topo-cloning and DNA sequencing.

During latency of HSV-1, the most abundant viral transcript is the latency-associated transcript (LAT), a noncoding RNA. Primary LAT is a low-abundance transcript of 8.5 kb in latently infected neurons. LAT is spliced, leading to accumulation of abundant 2.0 kb and 1.45 kb highly stable introns in the nucleus [29, 30] and LAT-encoded miRNAs [2, 3]. The 2.0 kb intron appears to be the major species in the latently infected neuron and the 1.45 kb intron flanked by “GC-AG” is produced via secondary pre-mRNA splicing using splice sites within the 2.0 kb intron [30]. The splice junction of the 2.0 kb LAT intron was identified by MapSplice 2 (Table 1), as was a novel splice junction flanking a 1.68 kb GC-AG intron (Fig 4A). The 5’ss of the 1.68 kb intron is the same as previously described second 5’ss of the 1.45 kb intron, which was previously shown to be an intron within the 2.0 kb intron (illustrated in Fig 4A). The alternative splice junctions for the 1.68 kb *LAT* was confirmed by RT-PCR and subsequent sequencing of the PCR fragments (Fig 4A). However, neither the 1.68 kb LAT intron nor the previously reported 1.45 kb LAT intron was readily detectable in KOS or d27-1 infected Vero cells by Northern hybridization (Fig 4A). Splicing of the 1.68 kb intron flanked by “GC-AG” was much less efficient as compared to the 2.0 kb intron (Fig 4B). Reads mapping to the splice junction for the 1.45 kb intron were not found in the high throughput data in either KOS or d27-1 infected cells (Fig 4B).

*ICP0*, one of the five IE genes, is an E3 ubiquitin ligase that promotes viral gene expression and inhibits host cell response. *ICP0* is non-essential at high multiplicities of infection [1]. Use of an alternative 3’ss located within intron 2 of the most common *ICP0* mRNA isoform generates a one amino acid polymorphism and was confirmed by RT-PCR and sequencing (Fig 4C). The frequency of using the alternative 3’ss for intron 2 is unaffected by the presence or absence of ICP27 (Fig 4D). An alternative minor 5’ splice site for *ICP0* intron 1 was also confirmed by RT-PCR (Fig 4C).

*UL15*, an essential γ gene, is required for viral DNA cleavage and packaging [1]. Four alternative 5’ss and one 3’ss were confirmed by RT-PCR and sequencing (Fig 4G). Each of the 5 alternative splices in *UL15* destroys or truncates the open-reading frame (ORF) of UL15 protein (Fig 4E). Although these splice junctions map to UL15, some splice junctions such as the junctions using 5’ss nt27264 and 28566 may represent readthrough transcripts transcribed from upstream promoters. The relative splicing efficiency of the known splice junction (2990^33581) of *UL15* (γ) was modestly increased from approximately 80% in KOS-infected cells to over 98% in d27-1 infected cells. Alternative splicing of *UL15* was very inefficient in KOS infected cells but was significantly increased in d27-1 infected cells (Fig 4F and 4G).

### Identification and confirmation of novel splice junctions mapping to 22 previously unknown spliced viral transcripts

We next analyzed splice junctions listed in S1 and S2 Tables mapping to previously unknown spliced genes in both KOS and d27-1 infected cells. Since low abundance splice junction reads likely represent very low splicing efficiency, only splice junctions greater than 65 nt with more than 30 reads from either KOS or d27-1 infected cells were selected for further verification. Viral transcripts with at least one splice junction verified experimentally by RT-PCR and sequencing are summarized in Table 2. Approximately 56 novel splice junctions were mapped to 20 viral genes, including in 2 previously uncharacterized viral transcripts mapping complementary to UL4 and UL22, named UL4-5C and UL22C. These novel splice junctions accounted for approximately 3.3% and 1.1% of total viral splice junctions for d27-1 and KOS infected cell (7 hpi), respectively. Since the cut-off for further analysis of putative splice junctions was set to 30 reads, the actual number of novel viral spliced transcripts almost certainly exceeds 22. These novel spliced genes include viral DNA replication-related early genes encoding UL5 protein (helicase/primase), UL52 protein (helicase/primase), UL12 protein (exonuclease), TK protein (thymidine kinase) and ICP8 protein (single-strand DNA-binding protein), as well as late genes encoding multiple glycoproteins (gH, gL, gE, gD and gB), and virus-host interaction factors including ICP34.5, US3, UL37, UL24, US11 and the virus-host shut-off protein (*VHS*) that are key viral virulence factors (Table 2). Splicing of these pre-mRNA transcripts destroys, truncates or internally deletes the open-reading frame (ORFs) for the protein encoded by each of these genes, indicating that ICP27-mediated aberrant pre-mRNA processing contributes to efficient expression of full-length viral proteins encoded by these genes with hidden splices.

**Table 2:** List of novel splice sites in previously unknown spliced genes and transcripts identified in the *ICP27* mutant virus and wide-type HSV-1 (KOS) infected cells.

Novel Spliced Viral Genes /transcripts	5’ Splice Junction	3’ Splice Junction	Reads (d27-1)	Reads (KOS)	Strand	Size of Intron (nt)	Classes	Verified by Sequencing
***ICP34*.*5***	125650	124046	804	3	-	1604	γ1, nonessential	Fig 5
	124467	124046	15	78	-	421		Fig 5
	124467	123186	0	46	-	1281		Fig 5
	125650	124073	35	0	-	1577		?
	125650	123186	96	33	-	2464		?
***UL5***	13881	13397	221	24	-	484	β, essential	Fig 7
***UL52***	109052	111003	93	6	+	1951	β, essential	Fig 7
	111281	114960	39	0	+	3679		Ref [38]
***pri-miR-H6/UDG***	6722	9770	65	2	+	3048	Unknown	Fig 6
	7618	9770	38	9	+	2152		Fig 6
***gL (UL1)***	9253	9770	123	4	+	517	γ1, essential	Fig 6
***UL37***	85696	80961	512	2	-	4735	γ1, essential	Fig 8
	83293	80961	465	4	-	2332		?
	81121	80961	89	1	-	160		?
	81571	80961	51	1	-	610		?
	83293	80864	35	0	-	2429		?
	85696	80864	58	0	-	4832		?
***US3***	135089	135293	1162	78	+	204	γ1, nonessential	Fig 8
	135089	135198	133	24	+	109		Fig 8
***gH (UL22)***	45630	44531	358	0	-	1099	γ, essential	Fig 8
	45626	44531	39	0	-	1095		**?**
***UL12***	25375	25140	749	0	-	235	β, nonessential	Fig 8
	27748	25140	39	0	-	2608		?
***gE (US8)***	142539	142641	706	0	+	102	γ1, nonessential	**?**
	142539	142666	38	0	+	122		**?**
	141171	142666	152	0	+	1495		Fig 8
	141171	142491	44	0	+	2420		?
***UL24***	47482	48016	44	9	+	534	γ1, nonessential	Fig 8
	46712	48016	7	246	+	1304		?
	47480	47943	35	0	+	491		?
***UL34***	69536	69868	35	1	+	332	γ1, essential	Fig 8
***VHS* (*UL41*)**	91311	91237	185	1	-	74	γ, nonessential	Fig 8
	91311	91166	142	1	-	145		Fig 8
***ICP8*(*UL29*)**	61447	60735	556	0	-	712	β, essential	Fig 8
	61447	60834	82	0	-	613		?
	61122	60735	172	1	-	387		?
	61122	60834	122	1	-	288		?
	62239	61395	77	0	-	844		?
	62148	61395	53	0	-	753		?
***TK* (*UL23*)**	47416	46696	82	0	-	720	β, nonessential	Fig 8
***US11*** ***/ICP47/US10***	145111	144553	96	0	-	558	γ, nonessential	Fig 8
	145130	144553	95	0	-	577		Fig 8
***gD (US6)***	138918	139058	249	3	+	140	γ, essential	Fig 8
	137670	138846	1	36	+	1176		?
	138252	138750	33	0	+	498		?
***gB (UL27)***	54946	54453	338	0	-	493	γ1, essential	Fig 8
	54454	54366	298	0	-	88		?
	54454	53733	123	0	-	721		?
	53923	53733	111	0	-	190		?
	55947	55220	149	0	-	727		?
	54454	54377	55	0	-	77		?
	55236	55079	46	103	-	157		?
***UL46 (VP11/12)***	99924	99253	38	0	-	671	γ1, essential	Fig 8
***UL42***^***b***^	93048 (93052)	94152	30	384	+	1104	β, essential	Fig 8
***UL4-5C***	12377	12921	14	381	+	544	Unknown	Fig 8
	12137	12921	5	96	+	784		?
***UL22C***	45684	46578	8	52	+	894	Unknown	Fig 8
***UL26/UL26*.*5***^***a***^	51974	52447	0	41	+	473	Unknown	Fig 8

^a^ intron is flanked by GC-AG.

^b^ A GC-AG intron was predicted by MapSplice 2 for *UL42* (93052^94152); however, RT-PCR and sequencing results suggest that a GT(U)-AG intron is used for *UL42* (93048^94152).

All newly confirmed introns possess canonical splice junctions flanked by GT(GU) and AG except for three transcripts, including the *LAT* 1.68 kb intron and *UL26*, for which splice junctions are flanked by GC and AG. Most of the splice junctions flanked by GC and AG including that of *UL49sp*, a recently identified spliced gene, could not be confirmed by RT-PCR in infected cells, suggesting that the majority of the predicted splice junctions flanked by GC-AG may represent sequencing error due to the high GC content of the HSV-1 genome. A GC-AG type intron was predicted for *UL42*; however, sequencing of the RT-PCR bands indicates that the “GT” located 4-bp downstream of the predicted “GC” is included in the intron. In contrast, the vast majority of splice junctions flanked by GT(GU) and AG could be verified by RT-PCR. Two novel splice junctions (*UL4-5C* and *UL22C*) were mapped antisense to the coding region of *UL4-5* and *UL22*, potentially representing read-through transcripts.

### ICP27 promotes expression of *ICP34*.*5* mRNA that is prematurely cleaved and polyadenylated from a proximal PAS by inhibiting use of the ICP34.5 5’ss

**Fig 5:**
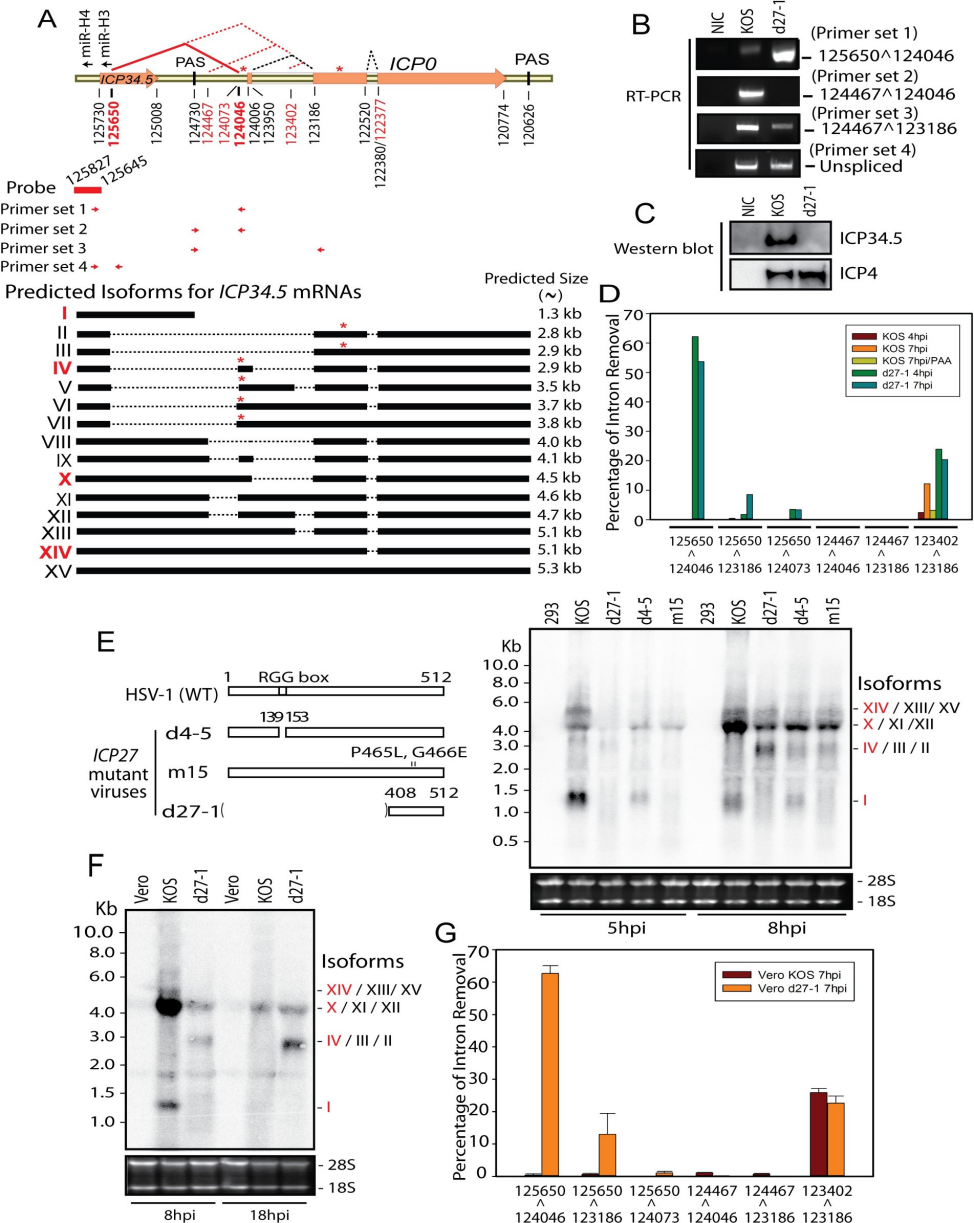
*ICP34*.*5* is a novel alternatively spliced gene for which pre-mRNA splicing and polyadenylation is tightly controlled by ICP27. (**A**) Diagram of the ICP34.5 and ICP0 gene locus. Potential *ICP34*.*5* mRNA isoforms I-XV, based on identified splice sites, with primers and probe used to detect them. “*” represents pre-mature stop codons in frame with the *ICP34*.*5* start codon. Relative locations of two latently expressed viral miRNAs, miR-H3 and miR-H4 [3], also named miR-I homolog and miR-LAT-ICP34.5 [2], are labeled on top of the diagram. Novel splice site positions are numbered in red and major novel splice sites (determined by the results presented below) are shown in bold red. (**B**) Confirmation of novel splice junctions mapping to the *ICP34*.*5-ICP0* region. cDNAs were prepared from total RNAs from HEK-293 cells infected with KOS or d27-1 at 7 hpi. The same cDNA samples were amplified with different RT-PCR primer sets illustrated in Panel (A). All the novel splice junctions in this region were confirmed by sequencing except for 125650^123186 and 125650^124073, likely due to relatively low levels and/or high GC content in the region. (**C**) Expression of HSV-1 ICP34.5 protein requires *ICP27*. Total proteins were prepared from HEK-293 cells infected with KOS or d27-1 at 7 hpi. ICP34.5 was detected using an anti-HSV-1 ICP34.5 antibody, and the same membrane was incubated with an anti-HSV-1 ICP4 antibody, after stripping. (**D**) Splicing using the novel splice sites mapping to the *ICP34*.*5-ICP0* region is only efficient when *ICP27* is absent. Relative splicing efficiency for the 5 novel splice junctions as well as the ICP0 intron 1 splice variant were quantitively analyzed using the high throughput sequencing data obtained from infected HEK-293 cells. 125650^124046 is the most efficient ICP34.5 splice junction in d27-1 infected cells. (**E**) Detection of *ICP34*.*5* splice isoforms in infected HEK-293 cells by Northern blot, with expected sizes of isoforms predicted in Panel (A). Total RNAs were prepared from HEK-293 cells infected with KOS and *ICP27* mutant viruses illustrated in the left panel. The probe mapped to exon 1 of *ICP34*.*5* is illustrated in Panel A. The 28S and 18S RNA shown in the bottom of the panel were used as loading control. The most abundant isoforms based on Panel D and Table 1 are labeled in red and confirmed by the blot. (**F**) Detection of *ICP34*.*5* isoforms in infected Vero cells by Northern blot, with expected sizes of isoforms predicted in Panel (A). Total RNAs were prepared from Vero cells infected with KOS and d27-1 at 8 hpi or 18 hpi. The 28S and 18S RNA shown in the bottom of the panel were used as loading control. (**G**) Quantitative splicing efficiency analysis for the 5 novel splice junctions mapping in the ICP34.5 region and ICP0 intron 1 splice variants in infected Vero cells. The mean relative splicing efficiency and standard deviation were calculated using the high throughput sequencing data obtained from infected Vero cells.

While HSV-2 ICP34.5 contains a 154-bp intron within the coding region and splicing inhibition of the HSV-2 *ICP34*.*5* pre-mRNA by ICP27 results in a truncated form of ICP34.5 [31, 32], HSV-1 *ICP34*.*5*, encoding the major viral neurovirulence factor, was not previously known to be a spliced gene. Splices from a 5’ splice junction (nt125650) in the coding region of *ICP34*.*5* to 3’ splice junctions (predominantly) at nt124046 in the 5’ UTR region of ICP0 and (less frequently) at nt123186, which is also the acceptor splice site of *ICP0* intron 1 were identified (Table 2 and Fig 5A). The predominant spliced *ICP34*.*5* transcript isoform (125650^124046) was readily detectable in d27-1 infected cells by RT-PCR but was barely detectable in KOS infected cells (Fig 5B). Two minor splice junctions (124467^124046 and 124467^123186) were also confirmed by RT-PCR (Fig 5B). Expression of HSV-1 ICP34.5 protein was abolished in d27-1 infected cells (Fig 5C). Further analysis of the splicing efficiency of these novel splice sites in the ICP34.5 region indicates that splicing of the ICP34.5 pre-mRNA is only efficient when ICP27 is absent (Fig 5D). When ICP27 is absent, 12560^124046 is the major splice with approximately 62% intron removal efficiency, followed by 123402^123186, 12560^123186 and 12560^124047. Splicing of 124467^124046 and 124467^123186 is very inefficient (below 0.5%) in the presence of ICP27 or not. Splicing of these novel introns is more efficient when ICP27 is absent. As discussed in Fig 3, splicing of *ICP0* intron 1 and intron 2 is efficient with slight intron retention in *ICP0* intron 1 in KOS infected cells. To be more rigorous, we also performed quantitative analysis of the novel alternative 5’ss (nt123402) of ICP0 intron 1. Splicing of this alternative ICP0 intron 1 appears to be enabled in the presence of ICP27, similar to the case with the alternative 3’ss (nt 122380 and 122377) of ICP0 intron 2 (Fig 4D). We next performed Northern blot of viral RNAs containing *ICP34*.*5* exon 1 in HEK-293 cells infected with wild-type HSV-1 or ICP27 mutants using a probe corresponding to ICP34.5 exon 1 (Fig 5E). m15 has a two-amino acid mutation on the C-terminal domain and d4-5 contains deletion of the N-terminal RGG RNA binding domain /SRPK-1 binding domain. A ~1.3 kb band corresponding to the monocistronic *ICP34*.*5* mRNA (Isoform I in Fig 5A), for which the *ICP34*.*5* polyadenylation signal (PAS) is within the newly identified intron, was abolished in d27-1 and m15 infected cells and significantly reduced in d4-5 infected cells, while a ~3 kb band corresponding to the novel *ICP34*.*5-ICP0* splice isoforms (Isoforms IV, III and II) was only present in d27-1 infected cells, consistent with the RT-PCR and Western blot findings (Fig 5C). A ~4.5 kb band corresponding to readthrough *ICP34*.*5-ICP0* splice isoforms (Isoforms X/XI/XII) is present in cells infected with both wild-type and ICP27 mutants. Other *ICP34*.*5* splicing isoforms at ~5 kb (Isoforms XIV/XIII/XV) predicted to exist based on these splice sites were also detected. Based on the relative splicing efficiency of these introns, the major products corresponding to the 1.3 kb, 3 kb, 4.5 kb and 5 kb Northern blot bands appear to be Isoform I, IV, X and XIV, respectively. Thus, the Northern blot results not only confirm ICP27-dependent splicing inhibition of *ICP34*.*5* pre-mRNAs, but also demonstrate that cleavage and polyadenylation of the monocistronic *ICP34*.5 mRNA is dependent on ICP27 and that the ICP27-dependent effects are dependent on ICP27 C-terminal sequences, as is the case for ICP27-mediated premature termination of cellular mRNAs in HSV-2 ICP27 transfected cells [20]. A similar result was also obtained in infected Vero cells (Fig 5F), confirming that expression of the 1.3 kb monocistronic *ICP34*.*5* mRNA as well as splicing involving the *ICP34*.*5* 5’ss is significantly increased when ICP27 is absent. We further analyzed the relative splicing efficiencies of the novel splice sites mapping to the *ICP34*.*5-ICP0* region using the RNA-Seq data obtained from infected Vero cells (Fig 5G). The quantitative results from Vero cells infected and sequenced in triplicate are comparable to those obtained from HEK-293 cells (Fig 5D). The splicing at these novel splice sites in the ICP34.5 region indicates that splicing of the ICP34.5 pre-mRNA is only efficient when ICP27 is absent (Fig 5D), also confirming that the 12560^124046 is the major splice among these novel ICP34.5 isoforms. Thus, the RNA that encodes HSV-1 ICP34.5 protein is efficiently expressed only when ICP27 is present to inhibit its splicing and promote 3’ formation using its own PAS. Quantitative analysis also confirmed that alternative splicing of ICP0 intron 1 does not appear to depend on absence of ICP27. These results in Vero cells demonstrate that ICP27-mediated aberrant viral pre-mRNA processing is not host cell type dependent.

### The latently and acutely expressed miR-H6 is encoded by previously unidentified introns for which splicing is regulated by ICP27

**Fig 6:**
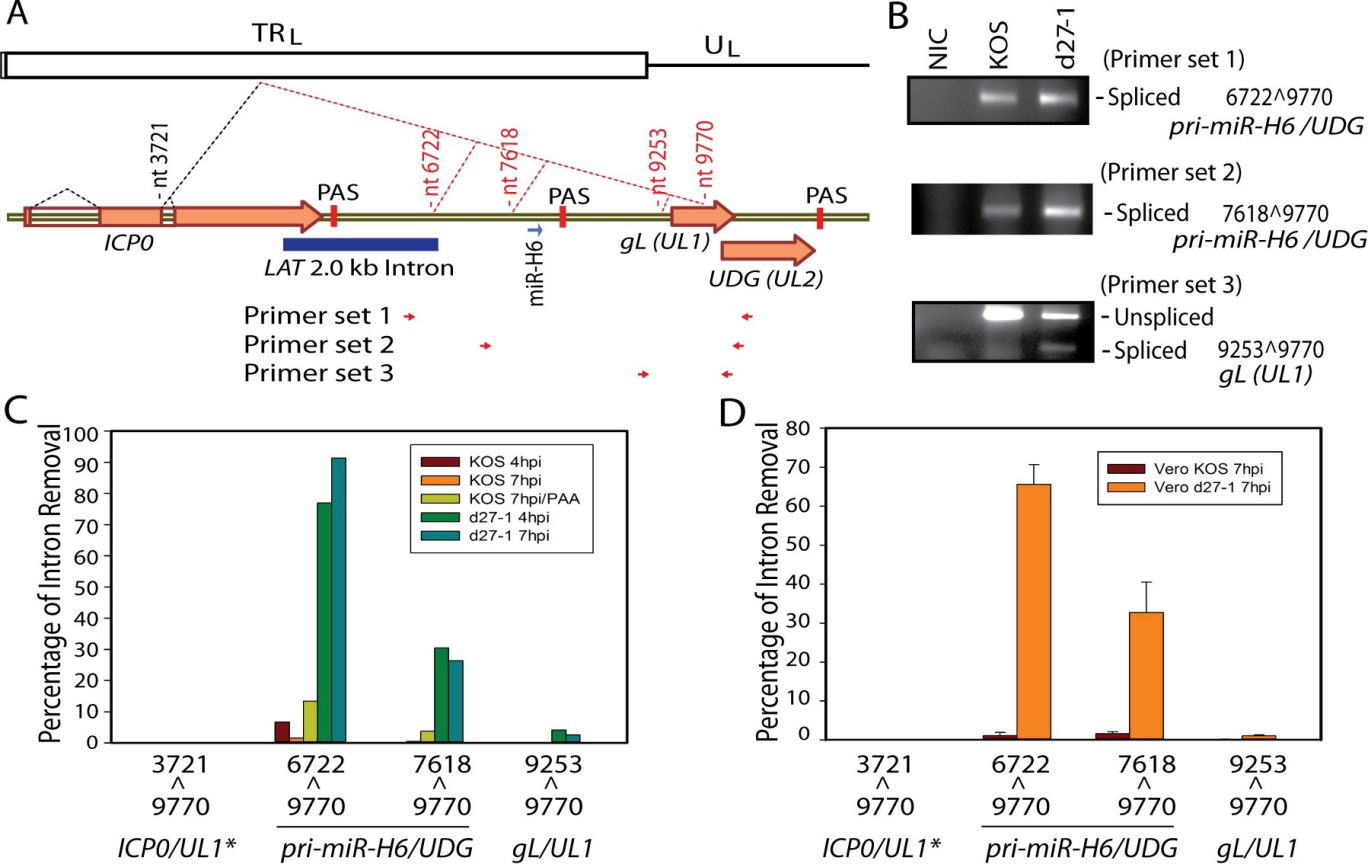
Latently and acutely expressed miR-H6 is encoded by alternatively spliced transcripts for which pre-mRNA splicing is regulated by ICP27. (**A**) Diagram showing the gene structure of the *ICP0*, *LAT*, *miR-H6*, *UL1* and *UL2* region. *ICP0*, miR-H6, and *LAT* are expressed from the repeat regions (shown as the terminal repeat TR_L_ in the diagram). *UL1* (*gL*) and *UL2* (*UDG*) share the same PAS and are expressed from the unique long region (U_L_). Primary *LAT* (not illustrated in the diagram) is transcribed antisense of *ICP0* and *pri-miR-H6*. The novel splice sites identified are numbered in red. The *LAT* TATA box is approximately 45 bp upstream of the novel 5’ss nt 7618 (relative to pri-miR-6/gL/UDG). A PAS is mapped approximately 724 bp downstream of the 5’ss nt7618. The relative locations of primers used in panel (B) are labeled on the bottom of the diagram. (**B**) The splice variants for *pri-miR-H6/gL/UDG* were confirmed by RT-PCR in cells infected with d27-1 or KOS. RT-PCR was performed using the primer sets and the same cDNA prepared for sequencing from KOS and d27-1 infected HEK-293 cells (7 hpi). The PCR bands corresponding to the spliced and unspliced products were cloned and the splice junction sequences were confirmed by DNA sequencing. (**C**) Quantitative analysis of splicing efficiency of the splice junctions mapping to the *ICP0*, *miR-H6* and *UL1/2* region using the RNA-Seq data obtained from infected HEK-293 cells. * The splicing efficiency of ICP0/UL1, a recently reported splice variants, is too low to be detected by this quantitative analysis (<0.03% of the ICP0 exon 2 junctions 3721^3861/3864). (**D**) Quantitative analysis of splicing efficiency of the splice junctions mapping to primary miR-H6-UL1 region using the RNA-Seq data obtained from infected Vero cells confirms the observation in infected HEK-293 cells.

The latently and acutely expressed HSV-1 miR-H6 maps antisense to the LAT promoter regions and was reported to play a role in regulating expression of ICP4, the other IE genes (along with ICP27) required for viral replication [3]. While the primary miR-H6 (*pri-miR-H6*) transcript remains unknown, transcripts in this region including AL and TAL antisense to LAT exon 1 were reported previously [33, 34]. Data obtained from HSV-2 suggest that transcription of HSV-2 pri-miR-H6 is likely initiated just upstream (relative to pri-miR-H6) of the LAT TATA box [35]. Here, we show that miR-H6 maps to newly identified introns that share a common 3’ss mapping to the C-terminus of *UL1* encoding glycoprotein L (gL) (Fig 6A), implying that pri-miR-H6 is also a spliced gene and miR-H6 is an intronic miRNA. This splice destroys the coding region of *gL* but the entire coding region of UL2, which encodes uracil-DNA glycosylase (UDG) is maintained. Thus, these two previously unidentified splice isoforms are named (*pri-miR-H6/UDG*). The gene structure of *pri-miR-H6/UDG* (7618^9770) resembles the gene structure of ICP34.5-ICP0, since both involve an intronic PAS located within 1 kb of the 5’ss. Both spliced isoforms (*pri-miR-H6/UDG* and the *gL* spliced isoforms) were confirmed by RT-PCR in KOS infected cells but were more abundant in d27-1 infected cells (Fig 6B). Quantitative splicing efficiency using the RNA-Seq data obtained from infected HEK-293 cells indicates that splicing of the *pri-miR-H6/UDG* transcript is efficient only when ICP27 is absent (Fig 6C). Quantitative splicing analysis using RNA-Seq of infected Vero cells (in triplicate) confirmed the results in HEK-293 cells (Fig 6D). UDG, which removes uracil that was mis-incorporated or arose by deamination in viral DNA in terminally differentiated neurons where endogenous cellular UDG activity is diminished, is required for DNA replication [36, 37]. This splicing that bridges the pri-miR-H6 promoter and the UDG coding sequences may potentially provide a mechanism for expression of UDG from the miR-H6 primary transcript during latency or early reactivation when ICP27 is absent.

A splice variant using the 5’ss (nt3721) of *ICP0* exon 2 and the 3’ss (9770) of *UL1* of *ICP0-UL1* (3721^9770) was very recently reported [38]. In d27-1 infected cells, there is indeed one read mapping to the splice junction of *ICP0-UL1* (3721^9770); however, there are no corresponding reads identified in KOS infected cells (S1 and S2 Tables). Splicing of *ICP0-UL1* (3721^9770) is below the detection limit and estimated to be less than 0.03% of the ICP0 exon 2 and exon 3 splice junctions in both KOS and d27-1 infected HEK-293 cells (Fig 6C). *ICP0-UL1* (3721^9770) is also under the detection limit in both KOS or d27-1 infected Vero cells (Fig 6D), suggesting splicing of *ICP0-UL1* (3721^9770) is much less efficient compared to other splice variants involving either the *ICP0* 5’ss (nt 3721) or *UL1* 3’ss (nt 9770).

### Key viral DNA synthesis-related early genes are spliced and pre-mRNA splicing of these genes is regulated by ICP27

**Fig 7:**
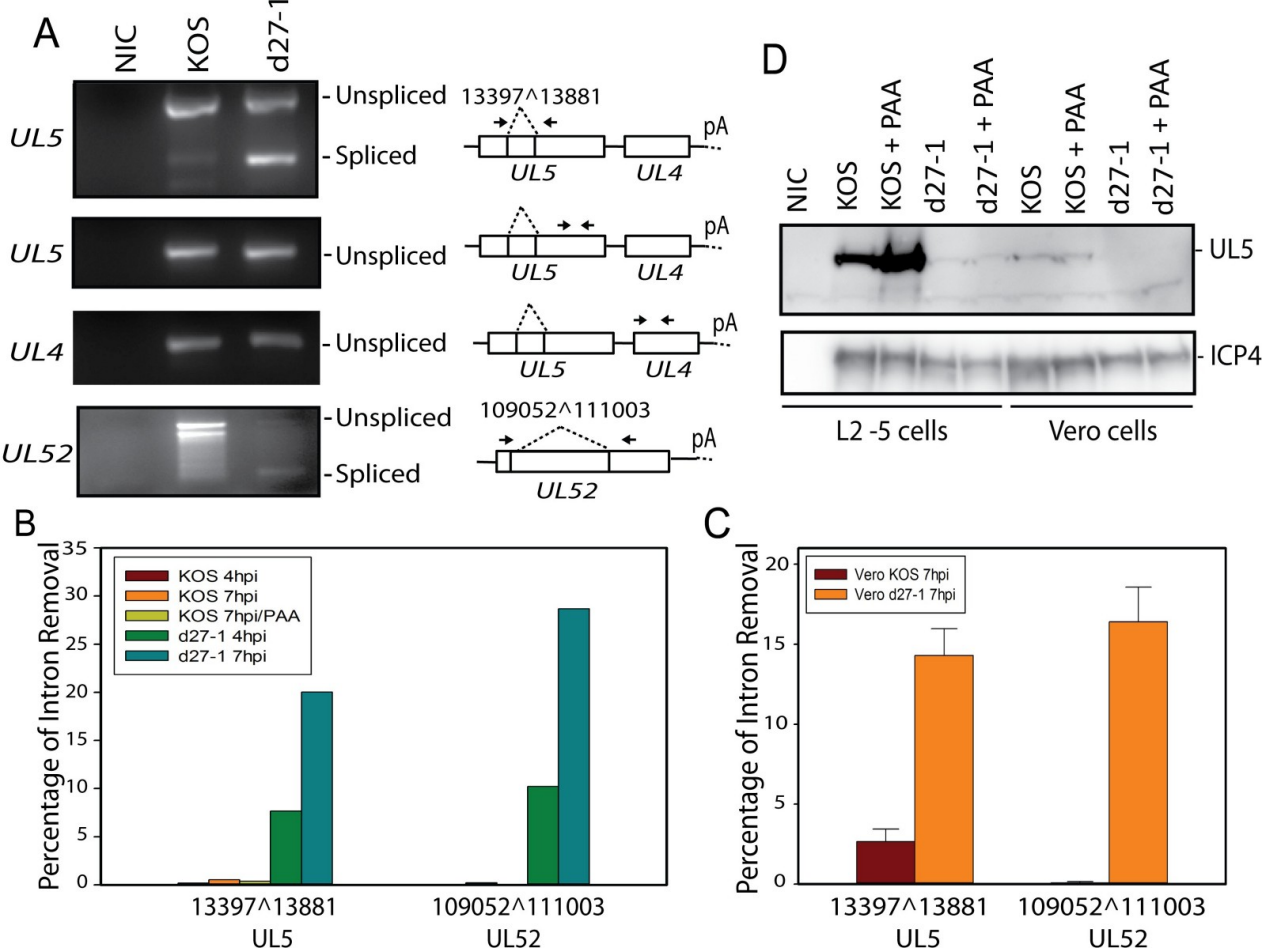
Replication associated early genes *UL5* and *UL52* are alternatively spliced genes and ICP27 inhibits the pre-mRNA splicing of these genes in a sequence specific manner. *UL5* (primase/helicase) and *UL52* (primase/helicase) were used as representative replication-associated genes. (**A**) cDNAs were prepared from HEK-293 cells infected with KOS or d27-1 at 7 hpi. RT-PCR using primers shown to right of each gel shows ICP27-dependent splicing of *UL52* and *UL5* but not *UL4* transcripts. (**B**) Quantitative splicing efficiency analysis for *UL5* and *UL52* using the RNA-Seq data obtained from infected HEK-293 cells. (**C**) Quantitative splicing efficiency analysis for *UL5* and *UL52* using the RNA-Seq data obtained from infected Vero cells at 7 hpi (performed in triplicate). (**D**) Western blot showing that the *ICP27* deletion in viral mutant d27-1 significantly reduces protein expression of *UL5*.

**Fig 8:**
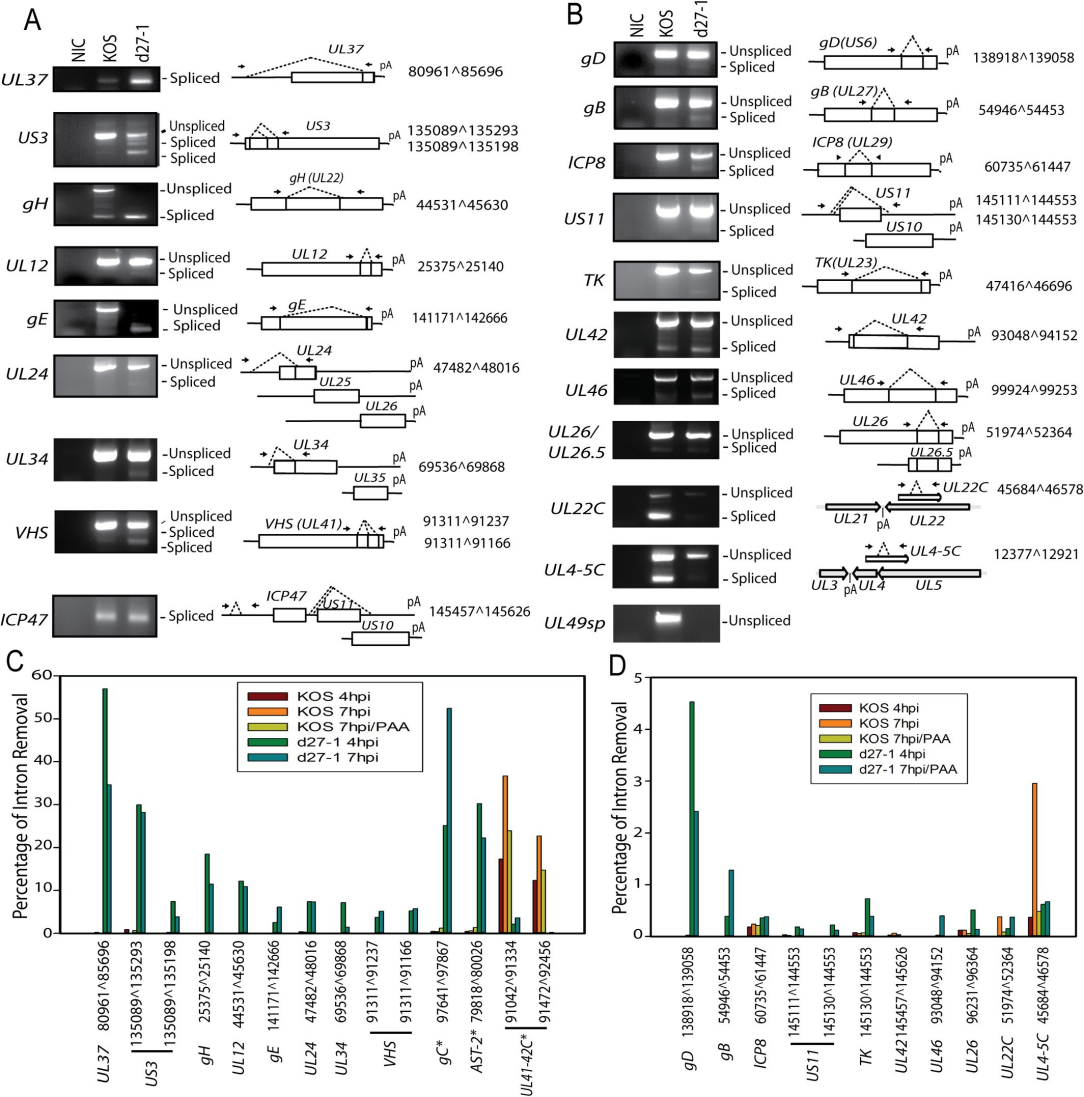
Pre-mRNA splicing of most of the identified novel spliced genes is inhibited by ICP27. The novel spliced isoforms listed in Table 1 and not discussed in other figures were verified by RT-PCR using total RNAs from KOS and d27-1 infected HEK-293 cells prepared at 7 hpi. Corresponding PCR bands were topo-cloned and verified by DNA sequencing. The RT-PCR results were grouped in Panel (**A**) for transcripts with splicing efficiency ≥5% or Panel (**B**) for transcripts with splicing efficiency < 5%. The RT-PCR for *ICP47* and *UL49sp* was used as a control. The diagrams on the right illustrate the relative location of the primers and the verified splices. Splicing efficiency of splice variants (confirmed in this study but not shown in other figures) was determined using the RNA-Seq data obtained from infected HEK-293 cells and is summarized in (**C**) for transcripts with relative splicing efficiency ≥5% or (**D**) for transcripts with relative splicing efficiency <5%. * Relative splicing efficiency of previously identified non-IE spliced genes including *gC*, *AST-2* and *UL41-42C* was also included in the analysis.

*UL5* and *UL52* are essential early genes encoding subunits of the viral helicase and primase complex, which is only weakly expressed in infected cells [39]. Splicing of UL5 and UL52 were confirmed by RT-PCR and sequencing (Fig 7A). Splicing efficiencies of *UL5* and *UL52* are approximately 20%-30% in HEK-293 cells and approximately 14–16% in Vero cells when ICP27 is not present; however, in the presence of ICP27, splicing of these two genes is efficiently inhibited (Fig 7B and 7C). Expression of UL5 protein is much reduced in d27-1 infected Vero cells or L2-5 cells that stably express *UL5* under the ribonucleotide reductase (*ICP6*) promoter (Fig 7D), consistent with previous findings on the role of ICP27 on UL5 expression [5]. The predicted protein corresponding to spliced *UL5* was not detected by Western blot, suggesting that it may be unstable. In addition to *UL5* and *UL52*, DNA replication-related early genes including *ICP8*, *TK*, and *UL12* are also spliced genes and ICP27 appears to inhibit splicing of these transcripts (Table 1 and Fig 8). While not all of these genes appear to be efficiently spliced in the absence of *ICP27*, the combined effect of splicing in several replication-related genes likely exceeds that in any single gene. The finding that in addition to increasing expression of specific viral DNA replication related early genes as reported previously [5] and as shown in Fig 2, ICP27 also contributes to maintain the functional full-length ORFs of targeted early genes by preventing co-transcriptional pre-mRNA splicing reveals a new regulatory mechanism at the pre-mRNA splicing level by which ICP27 controls viral DNA replication, and thus also expression of genes from newly synthesized DNA.

### ICP27 inhibits pre-mRNA splicing of most of the novel viral spliced genes identified

At least one of the splice junctions for each of the novel spliced genes listed in Table 2 was confirmed by RT-PCR and subsequent sequencing (Fig 8A and 8B). Furthermore, splicing is also much more efficient in d27-1 infected cells for most of the novel spliced genes, consistent with our observations for *ICP34*.*5*, *UL5*, *UL52* and *UL15* (Figs 4–6). The RT-PCR splicing patterns of a few inefficiently spliced transcripts (with relative splicing efficiency <5%) including *UL42*, *UL26*, *UL22C* and *UL4-5C* were not obviously different in the presence vs. absence of ICP27, and splicing of *UL26C* and *UL4-5C* appeared to increase in the presence of ICP27 (Fig 8B). The relative splicing efficiency of these novel spliced transcripts were also quantified using the RNA-Seq data. Splicing of *gC*, *AST-2* and *UL41-42C* was much less efficient in KOS infected cells and was significantly increased in d27-1 infected cells with only one major exception (Fig 8C and 8D). Splicing of *UL41-42C* appears to be reduced in d27-1 infected cells (Fig 8C); however, transfection of a *UL41-42C* minigene with or without an ICP27 expression plasmid does not obviously increase splicing efficiency of intron 1 and 2, suggesting that the discrepancy is likely due to extremely low abundance of *UL41-42C* transcripts in d27-1 infected cells. The splicing efficiency of *gC*, a previously identified spliced gene (γ2), increased dramatically from approximately less than 0.5% in KOS-infected cells to more than 50% in d27-1 infected cells, which is consistent with previous observations [31]. We made a similar observation for AST-2, a spliced transcript identified recently by long-read high throughput sequencing in wild-type HSV-1 infected cells using the PacBio sequencing system [22].

### ICP27-mediated aberrant pre-mRNA splicing is not cell type dependent

MRC-5 diploid human fibroblast cells and Vero African green monkey kidney epithelial cells were infected with KOS and d27-1. RT-PCR was performed using primers targeting representative novel spliced genes including *ICP34*.*5*, *UL5*, *UL52*, *gH*, *US3* and *gE* (S2A Fig). Similar splicing patterns (by RT-PCR) were observed in Vero and MRC-5 cells, confirming that ICP27-mediated effects on pre-mRNA splicing is not cell type dependent, consistent with the Northern blot results for *ICP34*.*5* mRNAs. Quantitative splicing efficiency analysis for the novel spliced genes (described in Table 2 and not described in the previous figures) using the RNA-Seq data obtained from infected Vero cells (in triplicate) was determined (S2B and S2C Fig). The gB splice junction (54946^54453) was under the detection limit of the quantitative analysis; however, all of the other novel splice junctions (presented in Table 2) were confirmed in the Vero RNA-Seq data, with splicing of most of these transcripts inhibited by ICP27 as in infected HEK-293 cells (Fig 8C and 8D). The transcripts in Vero and HEK-293 cells for which splicing was not significantly influenced by ICP27 include *UL41-42C*, *UL4-5C* and *UL22C*, which are transcribed complementary to known viral genes and have unknown significance in viral pathogenesis. The overall splicing efficiency in Vero cells (when ICP27 is absent) appears to be lower than that in HEK-293 cells, suggesting that cell type specific splice factors may influence the alternative splicing process of ICP27 targeted spliced genes.

### In addition to its role in alternative splicing and polyadenylation, ICP27 also regulates the accumulation of most of these novel spliced viral transcripts

Most viral transcripts except for IE transcripts are reduced in d27-1 infected cells compared to KOS infected cells (Fig 1). To further quantify the effect of ICP27 on the accumulation of ICP27-targeted genes, especially the functional open-reading frames, we further analyzed relative expression levels of ICP27 targeted novel spliced genes (listed in Tables 1 and 2) using RNA-Seq data. In this analysis, the RNA-Seq data were mapped to reference sequences selected to represent the total expression level of different splice variants as well as the ORFs of these genes. ICP27 does not appear to affect the accumulation of IE gene transcripts including *ICP0*, *ICP22*, *ICP4*, or *ICP47* (as indicated by the shared exon 2 sequence from *US11*), which serve as an infection control (S3A and S3B Fig). There is an approximately 8–10 fold reduction in levels of two γ2 late genes including *gC* and *LAT* in the presence of PAA, the DNA synthesis inhibitor, consistent with the previous finding that expression of *gC* and *LAT* in infected cell cultures depends on viral DNA replication [1, 40]. Accumulation of γ1 late and β genes appears to be relatively unaffected by DNA replication inhibition with PAA (< 2-fold changes); however, expression of many of these genes are significantly reduced when ICP27 is absent, indicating that ICP27’s role in regulating expression of these genes goes beyond just its influence on DNA replication. Accumulation of all other ICP27-targeted genes (ORFs) was positively correlated with ICP27. In d27-1 infected cells, reduced accumulation of ICP27 targeted transcripts ranged from approximately 470-fold for *gC* (*UL44*) to 1.7-fold for *UL15* (S3B Fig) relative to cells infected with strain KOS. The median fold-reduction of ICP27-targeted gene levels in d27-1infected cells was approximately 18-fold for infection at 4 hpi and 8-fold at 7 hpi. Substantial reduction of accumulation of the *gC* transcript in d27-1 infected cells is consistent with previous findings that ICP27 regulates *gC* mRNA accumulation through a responsive element (also a C-rich sequence) on the *gC* mRNA [41], in addition to its role in splicing inhibition of the *gC* transcript [21]. Following gC, the other most responsive genes including *UL26*, *US3*, *UL52*, *UL42*, and *LAT* were more than 60-fold reduced in d27-1 infected cells. Genes including *gH* (*UL22*), *UL37*, *VHS* (*UL41*) and *gE* (*US8*) were approximately 20-fold reduced at 5 hpi in d27-1 infected cells (S3B Fig). Expression of *ICP34*.*5* (as a total of transcript variants) was reduced by approximately 14-fold at 4 hpi and 9-fold at 7 hpi in d27-1 infected cells, consistent with the observation by Northern blot (Fig 5E and 5F). Consistently with previous findings [5, 42], replication-associated spliced early genes including *UL5*, *UL52*, *UL42* and *TK* were also reduced significantly in d27-1 infected cells (6- to 73 -fold), while expression of ICP8 (β) was only modestly reduced (approximately 2.5-fold) in d27-1 infected cells. Due to the complexity of the transcription patterns, including sharing of PAS by different viral genes, read-through transcripts and viral transcription in both directions in certain locations, the RNA-Seq data should be interpreted cautiously in the context of nearby viral gene structures. Thus, splice junctions mapping in unknown transcripts antisense to known ORFs including *UL22C*, *UL4-5C*, *AST-2* and *UL41-42C* were not included in this analysis. Nevertheless, these data are consistent with previously described results describing ICP27’s impact on virus gene accumulation [1], and illustrates an additional mechanism by which ICP27 regulates expression of its targeted viral genes.

## Discussion

By transcriptome analysis in cells infected with an HSV-1 ICP27-deletion mutant, we identified hundreds of novel splice sites mapping to the HSV genome and experimentally confirm at least 22 novel viral alternatively spliced genes, many of which are essential for efficient viral replication. We find that ICP27 inhibits splicing and promotes efficient polyadenylation using a proximal intronic PAS to facilitate expression of ICP34.5 and pri-miR-H6/UDG, both of which are novel spliced genes with intact ORFs. These findings not only fundamentally change our understanding of HSV-1 gene structure by quantitively mapping alternative splicing to more than one third of the known viral genes, but also reveal a novel mechanism by which ICP27 hijacks host splicing and polyadenylation for optimal viral gene expression. These findings also imply that during latent infection when ICP27 is absent, HSV-1 likely takes advantage of host splicing machinery to restrict expression of randomly activated antigenic viral genes to achieve immune evasion.

Analysis of high throughput RNA-Seq data for splice variants remains challenging. In this study, we used MapSplice 2 software to map pair-ended high throughput RNA-Seq data to an HSV-1 reference genome for novel splice junction discovery. This powerful approach was able to identify nearly all previously known splice junctions, as well as hundreds of novel splice junctions. We did not attempt to verify every single one of the hundreds of splice junctions identified (S1 and S2 Tables) but chose novel splice junctions with ≥ 30 reads for further experimental verification. We were able to confirm at least 22 previously unidentified spliced genes, recognizing that splice junctions with lower read counts could also likely be verified experimentally. For example, the very recently reported *ICP0-UL1* splice junction [38] was detected with 1 read in d27-1 infected HEK-293 cells (S1 Table). However, no *ICP0-UL1* splice junction read was identified using the RNA-Seq obtained in KOS infected cells or Vero cells infected with either KOS or d27-1 (S2 Table and Fig 6E), suggesting splice junctions even with extremely low read counts (e.g., one read count as listed in S1 Table) may be experimentally verifiable and thus that splicing is a widespread event in HSV-1 infected cells.

Due to the high GC content in HSV, we found that determination of splicing patterns by RT-PCR favors smaller PCR fragments representing spliced products, which is prone to overestimating splicing efficiency, especially for larger introns. Thus, we established a relative quantitative method by calculating the percentage of the exon-exon junction reads among the total exon-intron junctions and exon-exon junctions for each splice based on RNA-Seq data. This quantitative analysis is in agreement with the RT-PCR results for most of the spliced genes and is also consistent with Northern blot results in this this and previous studies, providing a powerful high throughput tool for better understanding the nature of a splice junction.

Little is known regarding to how HSV, a large DNA virus and known to contain very few spliced genes, escapes host pre-mRNA splicing machinery. Pre-mRNA splicing is a “default” process initiated by binding of U1 snRNP to the consensus 5’ss and of U2 snRNP to the consensus 3’ss. Splicing factors can regulate gene expression by influencing the inclusion or exclusion of particular exons in a gene’s mRNA [43]. We identified much more alternative splicing in a large group of novel viral spliced genes in infected cells when ICP27 is absent. When ICP27 is present, most of the splices in these novel viral spliced genes are largely silenced, suggesting the viral IE protein functions in a way analogous to a splicing factor to inhibit splicing both of its own transcripts and of a small percentage of host transcripts that resemble HSV genes in a gene/sequences specific manner [20]. Thus, HSV ICP27 likely coevolved with GC-rich viral genes that contain C-rich sequences and co-opts host splicing machinery to ensure the correctness of viral ORFs.

Interaction of U1 snRNP with 5’ss and PAS near the transcription start site controls the length of cellular mRNAs and promoter directionality [44–47]. We showed previously that ICP27 counteracts U1 snRNA’s function and promotes expression of hundreds of cellular short intronless transcripts resembling HSV genes [20]. Here, we show that ICP27 also toggles expression of the HSV-1 monocistronic *ICP34*.*5* mRNA that encodes the major viral neurovirulence factor by activating the proximal PAS located in the newly identified intron and inhibiting splicing of the newly identified intron (Fig 5). In ICP27 deletion mutant infected HEK-293 and Vero cells, approximately 64% to 75% of *ICP34*.*5* transcripts are spliced, destroying the coding sequence. Interestingly, *ICP34*.*5* is also negatively regulated by the two most abundant latently expressed miRNAs mapping antisense to its 5’ UTR and exon 1 [2, 3, 48, 49], further suggesting the importance to viral pathogenesis of tight regulation of HSV-1’s major neurovirulence factor. The distance between the newly identified 5’ss of *ICP34*.*5* and its PAS, which is located in the newly identified intron, is within the range (≤ 1 kb) typical for both ICP27 or U1 snRNP inhibitor mediated activation of intronic PAS of cellular genes [20, 44–46]. Because inhibition of U1 snRNP’s binding to a 5’ss also typically relieves its inhibition of polyadenylation at a downstream PAS (typically within 1 kb of the 5’ss) [44–46], and ICP27 is known to interact with U1 snRNP through its C-terminal domain, colocalizing with U1 and U2 snRNPs [14, 15], this suggests that U1 snRNP is likely to be involved in the mechanism of ICP27-mediated splicing inhibition and activation of intronic PAS.

The primary transcript of miR-H6, a latently and acutely expressed viral miRNA that was reported to target the key viral transactivator *ICP4* [3], has not been identified although it is certainly transcribed antisense to the *LAT*. There are reports of transcripts antisense to *LAT* such as AL RNA and TAL RNA antisense to *LAT* exon 1; however, the 5’ start sites and 3’ ends of these RNAs has not been determined [33, 34]. Identification of novel splice junctions for *pri-miR-H6* suggests that during latency when ICP27 is absent, *pri-miR-H6* is likely a spliced transcript. The gene structure around the splice 7616^9770 appears similar to that of *ICP34*.*5*, with a PAS mapping to approximately 700 bp downstream of the novel splice site (nt7618) and with the splicing inhibited by ICP27 (Fig 6). Splicing of the *pri-miR-H6-UDG* transcript during latency when ICP27 is absent may potentially lead to expression of UDG, a critical viral DNA repair enzyme. It is known that the endogenous enzymatic UDG activity is absent in terminally differentiated neurons.

In contrast to its role in virus-host shutoff by inhibiting expression of selected cellular transcripts [20], ICP27-mediated aberrant pre-mRNA processing is required to efficiently express full-length viral ORFs of ICP27 targeted viral genes during the coordinated temporal cascade of gene expression to promote efficient viral replication. Thus, ICP27 ensures the quality of its targeted viral transcripts. For example, inhibition of splicing of the low-abundance *UL5* and *UL52*, both essential early genes that encode the primase/helicase complex, may also contribute to a “switch” effect by which ICP27 regulates viral DNA replication and the many viral genes that rely on viral DNA replication. Splicing control of critical virulence factors, such as ICP34.5, as well as essential glycoproteins, such as gH and gC, likely also collectively contributes to the avirulent phenotype of ICP27 deletion mutants.

High GC content (approximately 68% to 70% in HSV) can contribute to intron retention or reduced splicing efficiency of mammalian genes [50, 51] and likely contributes to the fairly low observed baseline splicing efficiency of many of the novel spliced genes identified in this study. Even low splicing efficiency indicates that, these genes contain authentic U1 snRNP binding sites at 5’ss. U1 snRNP binding to 5’ss inhibits 3’ end formation at proximal PAS (typically <~1 kb from the transcription start site), a mechanism used by the cell to define the length and direction of its transcripts. Persistent U1 snRNP binding to cryptic 5’ss in proximity to a PAS can prevent accumulation of certain adenovirus, polyomavirus and bovine papillomavirus (BPV) mRNAs [52–54]. We thus hypothesize that some of the HSV genes, such as intronless short transcripts with cryptic 5’ss near a proximal PAS are also subject to U1 snRNP-mediated restriction and that ICP27 is required to remove U1 snRNP-mediated suppression of polyadenylation, analogously to ICP27’s role in promoting expression of cellular intronless transcripts polyadenylated from proximal intronic PAS as well as the *ICP34*.*5* monocistronic RNA [20] (Fig 5). Thus, the overall consequence of ICP27 mediated splicing inhibition during lytic infection likely ensures not only the quality (correct ORF and stabilized monocistronic mRNAs) but also the quantity (abundance) of certain ICP27-targeted viral genes, in addition to ICP27’s known role in RNA exporting and transcription [1, 55, 56].

While the virus might have been able to achieve expression of full-length genes via mutation of its splice sites, conserved splice site sequences among different HSV-1 strains suggests that ICP27-regulated aberrant posttranscriptional pre-mRNA processing likely has additional important functions. For example, this mechanism may also help reduce accidental expression of full-length viral antigens targeted by ICP27 during latency when ICP27 is absent. Indeed, recent studies revealed that HSV latency is not entirely quiescent and frequent switching on of certain antigenic lytic genes has been reported in immunological and molecular studies [57–59]. Thus, posttranscriptional regulation including splicing and the LAT-encoded miRNAs that disrupt major viral antigens and genes required for viral replication during latency when ICP27 is absent may contribute to immune-evasion and maintenance of viral latency. We also hypothesize that binding of U1 snRNP may play an important role in suppression of polyadenylation of certain ICP27 targeted viral genes during latency in the absence of ICP27, further contributing to immune-evasion and maintenance of viral latency.

Other viruses, such as papillomavirus, polyomavirus, adenovirus, retrovirus, and influenza virus, for which viral mRNA is transcribed in the nucleus, take advantage of the host pre-mRNA splicing and polyadenylation machinery to encode more viral products using limited viral DNA sequences through alternative splicing and polyadenylation to suit their viral life cycles [60]. Many of these viruses encode viral proteins to co-opt the cellular pre-mRNA processing machinery. For example, influenza NS-1 interacts with SRSR2, U6 snRNA and NS1-BP and altering host and viral mRNA splicing [60, 61]. NS-1 also interacts with CPSF30, polyadenylation factor required for the 3’ end processing of cellular pre-mRNA, resulted in reduced expression of cellular antiviral genes but not viral genes [61].

In contrast to many other viruses, the HSV-1 life cycle contains both a lytic infection phase, characterized by a coordinated temporal cascade, and a latent infection phase, characterized by the absence of significant viral antigen expression and viral DNA replication. During lytic infection, through its IE protein ICP27, HSV-1 activates PAS contained within the proximal intron and near the transcription start site of its genes, while inhibiting splicing of viral and cellular genes in a gene/sequence specific manner to achieve optimal viral gene expression. Many ICP27-targeted cellular genes are related to host immune response [20]. During latent infection in the absence of ICP27, HSV-1 likely uses host RNAi and splicing machinery to restrict expression of randomly activated viral antigens to achieve optimal immune evasion. Further investigation of the details of ICP27 mediated aberrant pre-mRNA processing will likely yield insight both into mechanisms of viral pathogenesis, potentially leading to identification of new targets for antiviral strategies, and into the mechanisms by which the cell itself controls alternative polyadenylation and splicing of selected genes.

## Methods

### Cells, virus and antibodies

HEK- HEK-293, MRC-5 and Vero cells were obtained from ATCC. L2-5 cells, a *UL5* mutant complementary cell line established from Vero cells that stably expresses HSV-1 UL5, were obtained from Dr. Sandra K. Weller [62]. HSV-1 strain KOS, HSV-1 *ICP27* mutant viruses (as shown in Fig 5E), and the V27 *ICP27*-complementing Vero cell line used to grow *ICP27* mutant viruses were obtained from Dr. Stephen Rice (University of Minnesota) [63, 64]. Anti-HSV ICP4 antibody (Santa Cruz) and anti-Flag antibody (Sigma) were sourced commercially. Anti-HSV-1 ICP34.5 antibody was obtained from Dr. Ian Mohr [65]. Anti-HSV-1 UL5 antibody was prepared from rabbits using peptides (AGGERQLDGQKPGPP and LTSNPASLEDLQRR).

### Transcriptome analysis of virus-infected cells

HEK-293 cells were infected with HSV-1 KOS or an ICP27 deletion mutant, d27-1 at a MOI of 5 in the presence or absence of the viral DNA replication inhibitor, phosphonoacetic acid (PAA), at 300 mg/mL. At four or seven hours post-infection (hpi), total RNAs were purified with the All-Prep DNA/RNA Kit (Qiagen). cDNA libraries were prepared from polyadenylated RNA using the TruSeq RNA sample Kit V2 (Illumina) and were sequenced on the NextSeq 500 according to the manufacturer’s instructions (Illumina). The six samples shared a single sequencer lane. Vero cells (in 6-well plates) were infected with HSV-1 KOS or d27-1 at a MOI of 5 in triplicate. At 7 hpi, total RNAs were purified with the All-Prep DNA/RNA Kit (Qiagen). cDNA libraries were prepared from polyadenylated RNA using the TruSeq RNA sample Kit V2 (Illumina) and were sequenced on the NextSeq 500. A total of 18 samples shared the same sequencing lane.

### Analysis of RNA-seq data using CLC Genomics Workbench

Viral gene expression profile was analyzed using CLC Genomics Workbench (QIAGEN) with an HSV-1 strain 17 (NC_001806.2) consensus sequence without the terminal repeat sequences as a reference (note: the genome sequence of strain 17 was only used in the CLC Genomics Workbench related analysis and all the exact splice site notations were based on HSV-1 KOS strain (JQ673480.1) as described below. CLC Genomics Workbench mapping of RNA-Seq data to genomes was performed without strand specificity. The cellular gene expression profile was analyzed using CLC Genomics Workbench and the human HG19 consensus sequence as a reference.

### Identification of viral splicing junctions by MapSplice 2

The RNA-Seq data were analyzed using MapSplice 2, software developed for mapping RNA-Seq data to a reference genome for splice junction discovery [66]. The HSV-1 KOS genome (JQ673480.1) was used as the reference sequence. Splice junctions with more than 30 reads were selected for further analysis.

### Relative splicing efficiency and gene expression level determined by RNA-seq

The RNA-Seq data were further mapped to the exon-exon junction (22 bp from each adjacent exon) or splice site junction (22 bp from the exon and 22 bp from the 22 bp from the adjacent intron) reference sequences using CLC Genomics Workbench. Each mapping result was visually checked to avoid partial or false alignments. Relative splicing efficiency was calculated using the percentage of exon-exon junctions reads in the total reads mapped relative to the total exon-exon junctions and splice site (5’ or 3’) junctions for each splice. For further analysis of relative expression of ICP27 targeted genes, 44 bp sequences from the N-terminus of coding sequences or the sequence upstream of the splice site of targeted genes were used as references. Read counts were normalized with the highest reads of KOS or d27-1 infected cells to generate the relative expression levels between KOS and d27-1 infected cells (S3A Fig). Fold-reduction comparisons were generated based on relative expression levels (S3B Fig).

### Analysis of conservation of novel splice sites

All identified novel 5’ss sequences (3 bases in exon and 6 bases in intron) and the 3’ss sequences (20 bases in the intron and 4 bases in the exon) were aligned to the genomic sequences of five commonly referenced laboratory and clinical HSV-1 strains including strain KOS, HSV-1 strain 17+ (NC_001806), strain F (GU734771), strain McKrae (JX142173), and strain H129 (GU734772). The strength of the splice sites were measured by MaxEntScan [67].

### Plasmids, primers and probes

pICP27, an HSV-2 expression vector, was described previously [32]. HSV-2 *ICP27* mutant plasmids including pΔR2 and pM15 were obtained from Dr. Masatoshi Hagiwara (Tokyo Medical and Dental University) [68]. The HSV-1 *ICP34*.*5-*specific DNA probe template (nt 125645–125827) containing 97 bp of the 5’ UTR sequence and 86 bp of the exon 1 sequence upstream of the novel 5’ss (as illustrated in Fig 5A) was prepared from plasmid constructed using PCR fragment by oST1076 and oST1075B. Oligonucleotide primers and synthesized DNA fragments are included in S3 Table.

### Western blot, RT-PCR and Northern blot

HEK-293 cells, MRC-5 cells, Vero cells or L2-5 cells were infected with viruses indicated in the figures at a multiplicity of infection (MOI) of 5. Total protein or RNAs were prepared at different time points post inoculation. Western blot was performed using the antibodies described above. For RT-PCR, total RNAs were extracted using All-Prep DNA/RNA kits (Qiagen). The primer sequences are listed in S3 Table. The RT-PCR bands shown in the figures that correspond to novel splice junctions were further confirmed by Topo cloning and sequencing. HEK-293 cells were transfected with plasmids indicated in Fig 6D using Lipofectamine 2000 (Invitrogen). Total protein or RNAs were prepared 24 hours post transfection. For Northern blots, total RNAs were prepared from HEK-293 cells or Vero cells infected with HSV-1 KOS strain or *ICP27* mutants by TRIzol (Invitrogen). Approximately 30 μg of total RNAs were separated in a formaldehyde denaturing 1.2% agarose gel (Life Technologies). After transfer to GeneScreen Plus hybridization transfer membrane (Perkin-Elmer), the membrane was incubated in NorthernMax hybridization buffer (ThermoFisher Scientific) at 58°C overnight with an HSV-1 ICP34.5-specific probe labeled with [α-32P] dCTP using a random priming kit (Promega).

## Supporting information

S1 Table List of all splice sites identified by MapSplice 2 in *ICP27* mutant virus-infected cells (7hpi). (PDF)

S2 Table List of all splice sites identified by MapSplice 2 in wild-type HSV-1 strain KOS-infected cells (7hpi). (PDF)

S3 Table Sequences of oligonucleotide primers, probes and point mutations for splicing reporter genes. (PDF)

S1 Fig ICP27 deletion reduces expression of non-α viral mRNAs in infected vero cells. RNA sequences from Vero cells infected with an HSV-1 ICP27 deletion mutant (d27-1) or its wild-type parental strain (KOS) at 7 hpi (in triplicate) were aligned to the HSV-1 genome (after removal of terminal repeat sequences, which are represented by internal repeats) and graphed as number of viral reads at each genome location. Genome positions of HSV genes relative to the trimmed genome are shown under the graph. Expression of HSV-1 IE genes including *RL2* (*ICP0*), *RS1* (*ICP4*), *US1* (*ICP22*) and *US12* (*ICP47*) labelled in red was similar between KOS or d27-1 infected cells. IE gene UL54 (ICP27) is not detectable in d27-1 infected cells since the coding region of UL54 was deleted in d27-1.
(TIF)

S2 Fig Verification of representative novel spliced genes in different cell lines. (**A**) cDNAs were prepared from total RNAs from KOS or d27-1 infected MRC-5 cells at 7hpi (left) or Vero cells at 8 hpi (right). Novel spliced isoforms were amplified using primers specific to the representative genes listed. The diagrams in the middle illustrate the relative location of the primers and the verified splice sites. Splicing efficiency of spliced variants listed in Table 2 but not described in other figures were determined by mapping the high throughput sequencing data obtained from infected Vero cells (in triplicate) (**B**) for transcripts with relative splicing efficiency ≥5% or (**C**) for transcripts with relative splicing efficiency <5%. *Relative splicing efficiency of previously identified non-IE spliced genes including *gC*, *AST-2* and *UL41-42C* were also included in the analysis.
(TIF)

S3 Fig Effect of ICP27 on accumulation of ICP27-targeted transcripts. (**A**) The RNA-Seq reads from infected HEK-293 cells at 4 and 7 hpi with KOS or d27-1 in the presence of PAA or not were mapped to 44bp reference sequences of the genes listed. The reference sequences for *gL*, *UL24*, *US11* and *UL15* were taken from sequences immediate downstream of the 3’ss in order to represent coding sequences. The reference sequence for *ICP22* (*US1*) was taken from the end of its coding sequences in order to distinguish ICP22 from *ICP47*, for which sequences near their splice sites are the same. The first 44 bp sequences following the start codon was used for the reference sequence for *ICP4* as a control. All other reference sequences were taken from immediately upstream of the 5’ss of the genes. The expression level was normalized to the most abundant reads obtained among KOS and d27-1 infected cells. Results should be cautiously interpreted since some viral genes may share the same PAS. For example, although the US11 reference sequence was taken from its exon 2 coding region, *ICP47 (US12)* transcripts also share the same PAS. (**B**) The data presented in the panel (**A**) was replotted to show relative fold reduction.
(TIF)
