# Supplementary material for: Hidden regulation of herpes simplex virus 1 pre-mRNA splicing and polyadenylation by virally encoded immediate early gene ICP27
Source: PLoS Pathog. 2019 Jun 17;15(6):e1007884. doi: 10.1371/journal.ppat.1007884 (PMC6597130; doi:10.1371/journal.ppat.1007884)
Supplement: S1 Table — (PDF) [file ppat.1007884.s001.pdf]

Splice Junction (nt)	Splice Junction (nt)	Strand	Junction Name	Read Counts	Intron Type	Intron Size (nt)
145457	145625	-	JUNC_443	75081	CTAC	168
132232	132400	+	JUNC_372	30190	GTAG	168
2291	3055	+	JUNC_8	16252	GTAG	764
29990	33581	+	JUNC_98	3573	GTAG	3591
122380	122520	-	JUNC_336	2867	CTAC	140
122377	122520	-	JUNC_335	1845	CTAC	143
135089	135293	+	JUNC_397	1162	GTAG	204
25140	25375	-	JUNC_68	749	CTAC	235
142539	142641	+	JUNC_428	706	GTAG	102
123186	123950	-	JUNC_348	621	CTAC	764
60735	61447	-	JUNC_198	556	CTAC	712
80961	85696	-	JUNC_262	512	CTAC	4735
2291	3685	+	JUNC_11	502	GTAG	1394
80961	83293	-	JUNC_259	465	CTAC	2332
124046	125650	-	JUNC_353	395	CTAC	1604
44531	45630	-	JUNC_120	358	CTAC	1099
145457	145644	-	JUNC_444	354	CTAC	187
54453	54946	-	JUNC_168	338	CTAC	493
54366	54454	-	JUNC_163	298	CTAC	88
141171	142641	+	JUNC_422	286	GTAG	1470
138918	139058	+	JUNC_411	249	GTAG	140
13397	13881	-	JUNC_51	221	CTAC	484
28566	33581	+	JUNC_89	206	GTAG	5015
91237	91311	-	JUNC_287	185	CTAC	74
97641	97867	+	JUNC_300	173	GTAG	226
60735	61122	-	JUNC_197	172	CTAC	387
132213	132400	+	JUNC_371	171	GTAG	187
33431	33581	+	JUNC_107	169	GTAG	150
141171	142666	+	JUNC_423	152	GTAG	1495
55220	55947	-	JUNC_177	149	CTAC	727
91166	91311	-	JUNC_286	142	CTAC	145
135089	135198	+	JUNC_396	133	GTAG	109
28566	29957	+	JUNC_88	130	GTAG	1391
9253	9770	+	JUNC_37	123	GTAG	517

53733	54454	-	JUNC_157	123	CTAC	721
60834	61122	-	JUNC_199	122	CTAC	288
123067	123161	+	JUNC_343	122	GCAG	94
53733	53923	-	JUNC_155	111	CTAC	190
591	2195	+	JUNC_3	102	GTAG	1604
591	3055	+	JUNC_4	96	GTAG	2464
144553	145111	-	JUNC_434	96	CTAC	558
55220	55913	-	JUNC_176	95	CTAC	693
144553	145130	-	JUNC_435	95	CTAC	577
27364	33581	+	JUNC_79	94	GTAG	6217
109052	111003	+	JUNC_321	93	GTAG	1951
141171	141940	+	JUNC_420	91	GTAG	769
80961	81121	-	JUNC_251	89	CTAC	160
29033	29957	+	JUNC_95	87	GTAG	924
46696	47416	-	JUNC_130	82	CTAC	720
60834	61447	-	JUNC_200	82	CTAC	613
61395	62239	-	JUNC_207	77	CTAC	844
12413	12997	-	JUNC_47	73	CTAC	584
6722	9770	+	JUNC_33	65	GTAG	3048
148160	148220	-	JUNC_447	64	CTGC	60
80864	85696	-	JUNC_249	58	CTAC	4832
54377	54454	-	JUNC_164	55	CTAC	77
28803	29957	+	JUNC_91	54	GTAG	1154
61395	62148	-	JUNC_206	53	CTAC	753
80961	81571	-	JUNC_253	51	CTAC	610
2839	3055	+	JUNC_16	49	GTAG	216
55079	55236	-	JUNC_174	46	CTAC	157
47482	48016	+	JUNC_138	44	GTAG	534
141171	142491	+	JUNC_421	44	GTAG	1320
90100	90155	-	JUNC_278	40	CTGC	55
25140	27748	-	JUNC_69	39	CTAC	2608
44531	45626	-	JUNC_119	39	CTAC	1095
55079	55211	-	JUNC_173	39	CTGC	132
111281	114960	+	JUNC_325	39	GTAG	3679
7618	9770	+	JUNC_34	38	GTAG	2152

99253	99924	-	JUNC_303	38	CTAC	671
142539	142666	+	JUNC_429	38	GTAG	127
3721	3861	+	JUNC_21	36	GTAG	140
47482	47973	+	JUNC_137	35	GTAG	491
69536	69868	+	JUNC_225	35	GTAG	332
80864	83293	-	JUNC_247	35	CTAC	2429
124073	125650	-	JUNC_357	35	CTAC	1577
138252	138750	+	JUNC_404	33	GTAG	498
61723	62239	-	JUNC_217	32	CTAC	516
93048	94152	+	JUNC_298	30	GCAG	1104
80961	88335	-	JUNC_264	28	CTAC	7374
91042	91334	+	JUNC_282	28	GTAG	292
61723	62148	-	JUNC_216	27	CTAC	425
14249	15328	-	JUNC_56	25	CTAC	1079
134556	135070	-	JUNC_391	24	CTAC	514
138266	138750	+	JUNC_407	24	GTAG	484
3721	3864	+	JUNC_22	23	GTAG	143
91573	92354	-	JUNC_294	23	CTAC	781
138918	139032	+	JUNC_409	23	GTAG	114
31415	33581	+	JUNC_101	20	GTAG	2166
54453	55221	-	JUNC_169	20	CTAC	768
9944	117935	-	JUNC_39	20	CTAC	107991
25243	25375	-	JUNC_71	18	CTAC	132
145393	145400	+	JUNC_439	18	CCAC	7
88472	89328	-	JUNC_274	17	CTAC	856
61723	61960	-	JUNC_215	16	CTAC	237
69613	69868	+	JUNC_226	15	GTAG	255
1774	2195	+	JUNC_6	14	GTAG	421
12377	12921	+	JUNC_44	14	GTAG	544
14198	15328	-	JUNC_55	14	CTAC	1130
32391	33581	+	JUNC_105	14	GTAG	1190
108732	109065	-	JUNC_318	14	CTAC	333
12127	12249	-	JUNC_40	13	CTAC	122
35677	36091	-	JUNC_108	12	CTAC	414
80961	82328	-	JUNC_255	12	CTAC	1367

124046	126343	-	JUNC_354	12	CTAC	2297
131043	131870	+	JUNC_367	12	GTAG	827
131043	131989	+	JUNC_368	12	GTAG	946
28070	33581	+	JUNC_85	11	GTAG	5511
29033	33581	+	JUNC_96	11	GTAG	4548
47499	47973	+	JUNC_140	11	GTAG	474
47499	48016	+	JUNC_141	11	GTAG	517
50719	59375	+	JUNC_145	11	GTAG	8656
132889	133073	-	JUNC_383	11	CTAC	184
132899	133082	-	JUNC_384	11	CTAC	183
142733	143189	+	JUNC_430	11	GTAG	456
47188	47416	-	JUNC_134	10	CTAC	228
99253	100138	-	JUNC_304	10	CTAC	885
2291	142641	+	JUNC_15	10	GTAG	140350
31499	33581	+	JUNC_102	9	GTAG	2082
35677	36182	-	JUNC_109	9	CTAC	505
45813	46073	-	JUNC_126	9	CTAC	260
48367	48523	+	JUNC_143	9	GTAG	156
80961	81312	-	JUNC_252	9	CTAC	351
80961	85397	-	JUNC_261	9	CTAC	4436
88472	88737	-	JUNC_273	9	CTAC	265
91573	92013	-	JUNC_293	9	CTAC	440
72165	121492	+	JUNC_229	9	GTAG	49327
138918	142666	+	JUNC_415	9	GTAG	3748
120209	145400	-	JUNC_329	9	CTAC	25191
145454	145625	-	JUNC_441	9	CTAC	171
2291	3264	+	JUNC_10	8	GTAG	973
2291	9770	+	JUNC_12	8	GTAG	7479
13397	15328	-	JUNC_52	8	CTAC	1931
41652	42190	+	JUNC_113	8	GTAG	538
45684	46578	+	JUNC_124	8	GTAG	894
54453	54853	-	JUNC_166	8	CTAC	400
55220	55847	-	JUNC_175	8	CTAC	627
59688	60227	-	JUNC_185	8	CTAC	539
60717	60772	+	JUNC_196	8	GTAG	55

79818	80023	+	JUNC_240	8	GTAG	205
80864	81571	-	JUNC_245	8	CTAC	707
105394	105580	+	JUNC_311	8	GTAG	186
138918	140516	+	JUNC_412	8	GTAG	1598
27007	33581	+	JUNC_76	7	GTAG	6574
27619	33581	+	JUNC_80	7	GTAG	5962
28803	33581	+	JUNC_92	7	GTAG	4778
31020	33581	+	JUNC_100	7	GTAG	2561
46712	47973	+	JUNC_132	7	GTAG	1261
46712	48016	+	JUNC_133	7	GTAG	1304
52292	52479	+	JUNC_148	7	GTAG	187
60663	61122	-	JUNC_195	7	CTAC	459
50719	116101	+	JUNC_146	7	GTAG	65382
123186	126343	-	JUNC_350	7	CTAC	3157
88000	130828	+	JUNC_271	7	GTAG	42828
132466	132472	+	JUNC_381	7	GGGG	6
138918	139038	+	JUNC_410	7	GTAG	120
132905	147592	-	JUNC_387	7	CTAC	14687
28866	29001	-	JUNC_93	6	CTAC	135
26997	33581	+	JUNC_75	6	GTAG	6584
80864	81121	-	JUNC_244	6	CTAC	257
80961	89328	-	JUNC_265	6	CTAC	8367
91472	92456	+	JUNC_288	6	GTAG	984
111258	114960	+	JUNC_324	6	GTAG	3702
123186	125650	-	JUNC_349	6	CTAC	2464
124046	126622	-	JUNC_355	6	CTAC	2576
119201	127039	-	JUNC_327	6	CTAC	7838
132903	133082	-	JUNC_385	6	CTAC	179
134833	135293	+	JUNC_395	6	GTAG	460
141507	141737	-	JUNC_425	6	CTAC	230
12137	12921	+	JUNC_43	5	GTAG	784
27751	33581	+	JUNC_81	5	GTAG	5830
31769	33581	+	JUNC_103	5	GTAG	1812
42950	43278	+	JUNC_117	5	GTAG	328
44739	45630	-	JUNC_122	5	CTAC	891

53234	53365	+	JUNC_150	5	GTAG	131
53762	54454	-	JUNC_159	5	CTAC	692
54194	54454	-	JUNC_161	5	CTAC	260
79818	80026	+	JUNC_241	5	GTAG	208
91042	91439	+	JUNC_284	5	GTAG	397
97760	97867	+	JUNC_301	5	GTAG	107
105628	106261	-	JUNC_313	5	CTAC	633
123186	123402	-	JUNC_347	5	CTAC	216
133162	133276	-	JUNC_389	5	CTAC	114
141171	141319	+	JUNC_419	5	GTAG	148
145858	149861	-	JUNC_445	5	CTAC	4003
591	2115	+	JUNC_1	4	GTAG	1524
2291	2633	+	JUNC_7	4	GTAG	342
12791	12921	+	JUNC_49	4	GTAG	130
14352	15328	-	JUNC_57	4	CTAC	976
16845	17525	-	JUNC_59	4	CTAC	680
22598	33581	+	JUNC_62	4	GTAG	10983
22943	33581	+	JUNC_63	4	GTAG	10638
46696	47009	-	JUNC_129	4	CTAC	313
46677	47416	-	JUNC_128	4	CTAC	739
54453	54601	-	JUNC_165	4	CTAC	148
65592	65663	+	JUNC_222	4	GTAG	71
69776	69868	+	JUNC_227	4	GTAG	92
80961	85706	-	JUNC_263	4	CTAC	4745
90999	91334	+	JUNC_280	4	GTAG	335
91573	91938	-	JUNC_292	4	CTAC	365
91472	94303	+	JUNC_289	4	GTAG	2831
99940	100118	+	JUNC_306	4	GTAG	178
105628	106229	-	JUNC_312	4	CTAC	601
107756	115045	-	JUNC_317	4	CTAC	7289
124126	125650	-	JUNC_359	4	CTAC	1524
132232	133396	+	JUNC_373	4	GTAG	1164
134786	135293	+	JUNC_394	4	GTAG	507
132447	135789	-	JUNC_380	4	CTAC	3342
138252	138773	+	JUNC_405	4	GTAG	521

132232	142641	+	JUNC_377	4	GTAG	10409
138918	142641	+	JUNC_414	4	GTAG	3723
145385	145625	-	JUNC_437	4	CTAC	240
2965	3055	+	JUNC_18	3	GTAG	90
26661	27748	-	JUNC_73	3	CTAC	1087
7618	33581	+	JUNC_35	3	GTAG	25963
25114	33581	+	JUNC_67	3	GTAG	8467
41652	42184	+	JUNC_112	3	GTAG	532
44807	45630	-	JUNC_123	3	CTAC	823
45684	46617	+	JUNC_125	3	GTAG	933
47499	47621	+	JUNC_139	3	GTAG	122
48367	48753	+	JUNC_144	3	GTAG	386
53246	53469	-	JUNC_151	3	CTAC	223
53762	53923	-	JUNC_158	3	CTAC	161
53733	54432	-	JUNC_156	3	CTAC	699
54453	54933	-	JUNC_167	3	CTAC	480
59889	60094	+	JUNC_186	3	GTAG	205
55220	61447	-	JUNC_179	3	CTAC	6227
61269	61447	-	JUNC_202	3	CTAC	178
61395	61447	-	JUNC_204	3	CTAC	52
61723	61934	-	JUNC_214	3	CTAC	211
62001	62239	-	JUNC_218	3	CTAC	238
53827	64203	+	JUNC_160	3	GTAG	10376
64298	65663	+	JUNC_220	3	GTAG	1365
4824	79075	-	JUNC_28	3	CTAC	74251
79195	80026	+	JUNC_239	3	GTAG	831
80961	82323	-	JUNC_254	3	CTAC	1362
80961	82589	-	JUNC_256	3	CTAC	1628
80961	83050	-	JUNC_258	3	CTAC	2089
80961	84875	-	JUNC_260	3	CTAC	3914
80864	88335	-	JUNC_250	3	CTAC	7471
89134	89246	+	JUNC_277	3	GTAG	112
98799	99952	+	JUNC_302	3	GTAG	1153
107360	107448	-	JUNC_316	3	CTAC	88
123186	123276	-	JUNC_346	3	CTAC	90

122556	123950	-	JUNC_339	3	CTAC	1394
124073	126343	-	JUNC_358	3	CTAC	2270
85089	132003	-	JUNC_267	3	CTAC	46914
54749	140051	+	JUNC_170	3	GTAG	85302
23939	140291	-	JUNC_64	3	CTAC	116352
141333	141436	+	JUNC_424	3	GTAG	103
138918	142491	+	JUNC_413	3	GTAG	3573
124046	145625	-	JUNC_356	3	CTAC	21579
2291	3124	+	JUNC_9	2	GTAG	833
3389	3453	-	JUNC_20	2	CTAC	64
4905	5305	-	JUNC_29	2	CTAC	400
4584	5537	-	JUNC_25	2	CTAC	953
4930	6889	-	JUNC_30	2	CTAC	1959
9253	9637	+	JUNC_36	2	GTAG	384
591	9770	+	JUNC_5	2	GTAG	9179
9490	9770	+	JUNC_38	2	GTAG	280
14012	15328	-	JUNC_54	2	CTAC	1316
14574	15328	-	JUNC_58	2	CTAC	754
25092	25375	-	JUNC_66	2	CTAC	283
27202	27748	-	JUNC_77	2	CTAC	546
28070	28420	+	JUNC_82	2	GTAG	350
27364	28456	+	JUNC_78	2	GTAG	1092
28070	29957	+	JUNC_84	2	GTAG	1887
29300	29957	+	JUNC_97	2	GTAG	657
26460	33581	+	JUNC_72	2	GTAG	7121
31996	33581	+	JUNC_104	2	GTAG	1585
32400	33581	+	JUNC_106	2	GTAG	1181
41652	43278	+	JUNC_114	2	GTAG	1626
46712	47621	+	JUNC_131	2	GTAG	909
47482	47621	+	JUNC_136	2	GTAG	139
41652	48016	+	JUNC_115	2	GTAG	6364
47212	48016	+	JUNC_135	2	GTAG	804
51653	52215	+	JUNC_147	2	GTAG	562
53087	53484	-	JUNC_149	2	CTAC	397
24985	53598	+	JUNC_65	2	GTAG	28613

42700	55789	+	JUNC_116	2	GTAG	13089
55220	56000	-	JUNC_178	2	CTAC	780
58749	58850	-	JUNC_182	2	CTAC	101
12402	58921	+	JUNC_46	2	GTAG	46519
58756	58951	+	JUNC_183	2	GTAG	195
60585	61122	-	JUNC_190	2	CTAC	537
60603	61447	-	JUNC_193	2	CTAC	844
61375	61447	-	JUNC_203	2	CTAC	72
60645	61690	+	JUNC_194	2	GTAG	1045
61609	61724	+	JUNC_211	2	GTAG	115
61637	61945	+	JUNC_212	2	GTAG	308
60834	62239	-	JUNC_201	2	CTAC	1405
65569	65663	+	JUNC_221	2	GTAG	94
68504	69868	+	JUNC_224	2	GTAG	1364
74616	75106	+	JUNC_230	2	GTAG	490
5623	77191	-	JUNC_32	2	CTAC	71568
78802	79164	+	JUNC_237	2	GTAG	362
75340	80023	+	JUNC_231	2	GTAG	4683
80864	85397	-	JUNC_248	2	CTAC	4533
55220	85696	-	JUNC_180	2	CTAC	30476
19523	86231	-	JUNC_61	2	CTAC	66708
87539	87674	-	JUNC_270	2	CTAC	135
88099	88163	-	JUNC_272	2	CTAC	64
89030	89328	-	JUNC_276	2	CTAC	298
58626	90762	-	JUNC_181	2	CTAC	32136
90474	90811	-	JUNC_279	2	CTAC	337
91042	91436	+	JUNC_283	2	GTAG	394
91573	91833	-	JUNC_291	2	CTAC	260
91573	93683	-	JUNC_296	2	CTAC	2110
103905	104082	-	JUNC_309	2	CTAC	177
107048	107130	-	JUNC_315	2	CTAC	82
111721	113130	+	JUNC_326	2	GTAG	1409
60108	116443	-	JUNC_189	2	CTAC	56335
38443	121168	+	JUNC_110	2	GTAG	82725
119352	121311	+	JUNC_328	2	GTAG	1959

63503	121336	+	JUNC_219	2	GTAG	57833
120936	121336	+	JUNC_331	2	GTAG	400
91141	121547	-	JUNC_285	2	CTAC	30406
121502	121641	+	JUNC_333	2	GTAG	139
120704	121657	+	JUNC_330	2	GTAG	953
2845	122520	-	JUNC_17	2	CTAC	119675
122788	122852	+	JUNC_341	2	GTAG	64
55052	123335	+	JUNC_172	2	GTAG	68283
3721	123396	+	JUNC_24	2	GTAG	119675
123117	123950	-	JUNC_344	2	CTAC	833
127218	128212	-	JUNC_360	2	CTAC	994
128777	129490	-	JUNC_363	2	CTAC	713
5498	131076	+	JUNC_31	2	GTAG	125578
99759	132035	-	JUNC_305	2	CTAC	32276
133153	133265	-	JUNC_388	2	CTAC	112
122544	133627	+	JUNC_337	2	GTAG	11083
122544	134523	+	JUNC_338	2	GTAG	11979
135503	136329	+	JUNC_400	2	GTAG	826
132232	138750	+	JUNC_374	2	GTAG	6518
132232	138773	+	JUNC_375	2	GTAG	6541
141171	141316	+	JUNC_418	2	GTAG	145
141161	141737	-	JUNC_417	2	CTAC	576
141021	142641	+	JUNC_416	2	GTAG	1620
144065	144071	+	JUNC_432	2	GGCG	6
143081	144354	-	JUNC_431	2	CTAC	1273
144618	144624	+	JUNC_436	2	AGCG	6
131007	146810	-	JUNC_366	2	CTAC	15803
145868	146814	-	JUNC_446	2	CTAC	946
131047	146850	+	JUNC_369	2	GTAG	15803
132903	147018	-	JUNC_386	2	CTAC	14115
145438	147030	-	JUNC_440	2	CTAC	1592
149645	150639	+	JUNC_449	2	GTAG	994
591	2168	+	JUNC_2	1	GTAG	1577
3375	3909	-	JUNC_19	1	CTAC	534
4600	4739	-	JUNC_26	1	CTAC	139

4815	4867	-	JUNC_27	1	CTAC	52
3721	9770	+	JUNC_23	1	GTAG	6049
12127	12288	-	JUNC_41	1	CTAC	161
12384	12921	+	JUNC_45	1	GTAG	537
12127	12997	-	JUNC_42	1	CTAC	870
19510	19743	+	JUNC_60	1	GTAG	233
28070	28456	+	JUNC_83	1	GTAG	386
28566	29397	+	JUNC_86	1	GTAG	831
29009	29604	-	JUNC_94	1	CTAC	595
28566	29715	+	JUNC_87	1	GTAG	1149
13089	33581	+	JUNC_50	1	GTAG	20492
26788	33581	+	JUNC_74	1	GTAG	6793
28582	33581	+	JUNC_90	1	GTAG	4999
30287	33581	+	JUNC_99	1	GTAG	3294
41413	41489	+	JUNC_111	1	GTAG	76
44739	45626	-	JUNC_121	1	CTAC	887
44382	45630	-	JUNC_118	1	CTAC	1248
47916	48110	-	JUNC_142	1	CTAC	194
46654	53505	+	JUNC_127	1	GTAG	6851
53504	53598	+	JUNC_152	1	GTAG	94
54352	54358	+	JUNC_162	1	CCCG	6
58835	58841	+	JUNC_184	1	GGGC	6
60010	60016	+	JUNC_187	1	GCTA	6
60042	60094	+	JUNC_188	1	GTAG	52
53730	60488	+	JUNC_154	1	GTAG	6758
60603	61122	-	JUNC_192	1	CTAC	519
60585	61447	-	JUNC_191	1	CTAC	862
61542	61594	+	JUNC_210	1	GTAG	52
61662	61744	+	JUNC_213	1	GTAG	82
61395	61934	-	JUNC_205	1	CTAC	539
68377	68558	+	JUNC_223	1	GTAG	181
78432	79164	+	JUNC_235	1	GTAG	732
79195	79630	+	JUNC_238	1	GTAG	435
78312	80023	+	JUNC_234	1	GTAG	1711
78432	80023	+	JUNC_236	1	GTAG	1591

71240	80026	+	JUNC_228	1	GTAG	8786
76164	80026	+	JUNC_232	1	GTAG	3862
77142	80026	+	JUNC_233	1	GTAG	2884
80387	80393	+	JUNC_243	1	GGTG	6
80864	82328	-	JUNC_246	1	CTAC	1464
80961	82640	-	JUNC_257	1	CTAC	1679
82558	83293	-	JUNC_266	1	CTAC	735
87068	87320	-	JUNC_268	1	CTAC	252
87068	88335	-	JUNC_269	1	CTAC	1267
88574	88737	-	JUNC_275	1	CTAC	163
90999	91439	+	JUNC_281	1	GTAG	440
91573	93394	-	JUNC_295	1	CTAC	1821
91472	97867	+	JUNC_290	1	GTAG	6395
96718	97867	+	JUNC_299	1	GTAG	1149
102274	107294	-	JUNC_308	1	CTAC	5020
108732	109458	-	JUNC_319	1	CTAC	726
108732	109845	-	JUNC_320	1	CTAC	1113
109178	111003	+	JUNC_323	1	GTAG	1825
91573	112083	-	JUNC_297	1	CTAC	20510
2291	114960	+	JUNC_13	1	GTAG	112669
109052	114960	+	JUNC_322	1	GTAG	5908
99951	116653	+	JUNC_307	1	GTAG	16702
61464	121311	+	JUNC_208	1	GTAG	59847
122772	122778	+	JUNC_340	1	GTGC	6
122332	122866	+	JUNC_334	1	GTAG	534
25140	123950	-	JUNC_70	1	CTAC	98810
123149	123950	-	JUNC_345	1	CTAC	801
124046	124467	-	JUNC_352	1	CTAC	421
13397	125650	-	JUNC_53	1	CTAC	112253
127989	128255	+	JUNC_361	1	GTAG	266
121175	129595	+	JUNC_332	1	GTAG	8420
130638	130828	+	JUNC_364	1	GTAG	190
104090	132047	+	JUNC_310	1	GTAG	27957
132157	132163	+	JUNC_370	1	CACC	6
132753	132759	+	JUNC_382	1	GCGT	6

54803	133238	+	JUNC_171	1	GTAG	78435
106197	133238	+	JUNC_314	1	GTAG	27041
122788	133238	+	JUNC_342	1	GTAG	10450
133723	133729	+	JUNC_390	1	GCTG	6
2291	135293	+	JUNC_14	1	GTAG	133002
79818	135293	+	JUNC_242	1	GTAG	55475
135089	135308	+	JUNC_398	1	GTAG	219
135089	135336	+	JUNC_399	1	GTAG	247
134556	135550	-	JUNC_392	1	CTAC	994
135634	136292	+	JUNC_401	1	GTAG	658
134556	136342	-	JUNC_393	1	CTAC	1786
61464	138509	+	JUNC_209	1	GTAG	77045
138266	138773	+	JUNC_408	1	GTAG	507
138252	138972	+	JUNC_406	1	GTAG	720
137670	139593	-	JUNC_402	1	CTAC	1923
132232	142491	+	JUNC_376	1	GTAG	10259
142166	142491	+	JUNC_426	1	GTAG	325
127989	142540	+	JUNC_362	1	GTAG	14551
132232	142666	+	JUNC_378	1	GTAG	10434
142166	142666	+	JUNC_427	1	GTAG	500
145391	145397	+	JUNC_438	1	CCCC	6
123186	145625	-	JUNC_351	1	CTAC	22439
130646	145625	-	JUNC_365	1	CTAC	14979
137670	145625	-	JUNC_403	1	CTAC	7955
145454	146810	-	JUNC_442	1	CTAC	1356
12413	146814	-	JUNC_48	1	CTAC	134401
144495	146985	+	JUNC_433	1	GTAG	2490
132232	147211	+	JUNC_379	1	GTAG	14979
53675	148013	-	JUNC_153	1	CTAC	94338
149602	149868	-	JUNC_448	1	CTAC	266
