# Supplementary material for: Hidden regulation of herpes simplex virus 1 pre-mRNA splicing and polyadenylation by virally encoded immediate early gene ICP27
Source: PLoS Pathog. 2019 Jun 17;15(6):e1007884. doi: 10.1371/journal.ppat.1007884 (PMC6597130; doi:10.1371/journal.ppat.1007884)
Supplement: S2 Table — (PDF) [file ppat.1007884.s002.pdf]

Splice Junction (nt)	Splice Junction (nt)	Strand	Junction Name	Read Counts	Intron Type	Intron Size (nt)
145457	145625	-	JUNC_26199	66113	CTAC	168
132232	132400	+	JUNC_22659	30732	GTAG	168
123186	123950	-	JUNC_22007	14506	CTAC	764
2291	3055	+	JUNC_215	14111	GTAG	764
91472	92456	+	JUNC_15179	3387	GTAG	984
122380	122520	-	JUNC_21823	2375	CTAC	140
3721	3861	+	JUNC_490	2302	GTAG	140
29990	33581	+	JUNC_3544	2098	GTAG	3591
122377	122520	-	JUNC_21822	1770	CTAC	143
3721	3864	+	JUNC_491	1705	GTAG	143
91042	91334	+	JUNC_15101	870	GTAG	292
123186	123402	-	JUNC_22006	548	CTAC	216
2839	3055	+	JUNC_282	497	GTAG	216
93048	94152	+	JUNC_15491	384	GCAG	1104
12377	12921	+	JUNC_1579	381	GTAG	544
90999	91334	+	JUNC_15097	369	GTAG	335
33431	33581	+	JUNC_3845	283	GTAG	150
46712	48016	+	JUNC_6662	246	GTAG	1304
4930	6889	-	JUNC_674	224	CTAC	1959
97641	97867	+	JUNC_16697	215	GTAG	226
119352	121311	+	JUNC_21471	205	GTAG	1959
145457	145644	-	JUNC_26200	202	CTAC	187
114061	114374	-	JUNC_21025	173	CTGC	313
55079	55236	-	JUNC_9087	103	CTAC	157
132213	132400	+	JUNC_22643	102	GTAG	187
55079	55211	-	JUNC_9086	97	CTGC	132
12137	12921	+	JUNC_1548	96	GTAG	784
123067	123161	+	JUNC_21969	92	GCAG	94
91042	91439	+	JUNC_15103	91	GTAG	397
135089	135293	+	JUNC_23510	78	GTAG	204
41652	42190	+	JUNC_5849	59	GTAG	538
96231	96364	+	JUNC_16232	57	GCAG	133
105692	106205	+	JUNC_19396	56	GCAG	513

45684	46578	+	JUNC_6470	52	GTAG	894
54006	54085	+	JUNC_8567	51	GCAG	79
1774	3055	+	JUNC_116	48	GTAG	1281
90100	90155	-	JUNC_14858	47	CTGC	55
123186	124467	-	JUNC_22009	46	CTAC	1281
51974	52447	+	JUNC_7703	42	GCAG	473
1774	2195	71	JUNC_115	42	GTAG	421
148160	148220	-	JUNC_26463	40	CTGC	60
129637	129697	+	JUNC_22363	40	GCAG	60
124046	124467	-	JUNC_22117	36	CTAC	421
91042	91436	+	JUNC_15102	36	GTAG	394
23936	140270	-	JUNC_2568	29	CTAC	116334
137670	138846	-	JUNC_23920	29	CTAC	1176
4930	6613	-	JUNC_673	28	CTGC	1683
119628	121311	+	JUNC_21480	28	GCAG	1683
9944	117935	-	JUNC_1157	27	CTAC	107991
27364	33581	+	JUNC_3330	26	GTAG	6217
90999	91439	+	JUNC_15099	25	GTAG	440
13397	13881	-	JUNC_1674	24	CTAC	484
135089	135198	+	JUNC_23509	24	GTAG	109
77223	98933	+	JUNC_12915	22	GTAG	21710
16845	17525	-	JUNC_1979	22	CTAC	680
92997	94208	-	JUNC_15465	21	CTAC	1211
138509	138605	-	JUNC_24102	21	CTGC	96
52413	120197	-	JUNC_7930	20	CTAC	67784
61554	71309	+	JUNC_10688	20	GCAG	9755
82823	82888	+	JUNC_13403	20	GCAG	65
12791	12921	+	JUNC_1624	19	GTAG	130
131043	131870	+	JUNC_22454	18	GTAG	827
123186	125650	-	JUNC_22010	17	CTAC	2464
95005	95093	-	JUNC_16130	17	CTGC	88
23936	140291	-	JUNC_2570	16	CTAC	116355
591	3055	+	JUNC_26	16	GTAG	2464
45920	47326	+	JUNC_6517	16	GCAG	1406

145987	146814	-	JUNC_26336	16	CTAC	827
93006	94152	+	JUNC_15474	15	GTAG	1146
90999	91436	+	JUNC_15098	15	GTAG	437
23939	140291	-	JUNC_2573	14	CTAC	116352
5557	98933	+	JUNC_809	14	GTAG	93376
98035	106333	+	JUNC_16862	14	GTAG	8298
137670	138978	-	JUNC_23921	14	CTAC	1308
51149	51649	-	JUNC_7385	14	CTGC	500
23939	140270	-	JUNC_2572	13	CTAC	116331
93064	94169	-	JUNC_15506	13	CTGC	1105
53234	53365	+	JUNC_8179	13	GTAG	131
96500	120197	-	JUNC_16326	12	CTAC	23697
27619	33581	+	JUNC_3371	12	GTAG	5962
102586	102887	-	JUNC_18181	12	CTGC	301
119450	123950	-	JUNC_21476	11	CTAC	4500
2291	6791	+	JUNC_218	11	GTAG	4500
111281	113709	+	JUNC_20648	11	GTAG	2428
31415	33581	+	JUNC_3728	11	GTAG	2166
88472	88737	-	JUNC_14426	11	CTAC	265
120209	145400	-	JUNC_21492	10	CTAC	25191
25114	33581	+	JUNC_2768	10	GTAG	8467
123117	123950	-	JUNC_21985	10	CTAC	833
2291	3124	+	JUNC_216	10	GTAG	833
120933	121336	+	JUNC_21576	10	GCAG	403
79818	80023	+	JUNC_13056	10	GTAG	205
105394	105580	+	JUNC_19266	10	GTAG	186
23302	23401	-	JUNC_2451	10	CTGC	99
107227	122608	+	JUNC_20010	9	GTAG	15381
123186	126343	-	JUNC_22011	9	CTAC	3157
7618	9770	+	JUNC_892	9	GTAG	2152
31499	33581	+	JUNC_3739	9	GTAG	2082
92997	94160	-	JUNC_15464	9	CTGC	1163
87659	88335	-	JUNC_14235	9	CTAC	676
47482	48016	+	JUNC_6917	9	GTAG	534

90999	91320	+	JUNC_15096	9	GTAG	321
40979	41118	+	JUNC_5766	9	GCAG	139
50719	66308	+	JUNC_7326	8	GTAG	15589
4905	5308	-	JUNC_658	8	CTGC	403
124073	124467	-	JUNC_22130	8	CTAC	394
96270	96470	-	JUNC_16252	8	CTGC	200
93064	93181	-	JUNC_15505	8	CTGC	117
76407	76468	+	JUNC_12873	8	GTAG	61
52413	84407	-	JUNC_7927	7	CTAC	31994
30854	54333	+	JUNC_3646	7	GTAG	23479
27007	33581	+	JUNC_3290	7	GTAG	6574
116001	120192	+	JUNC_21310	7	GTAG	4191
46712	48753	+	JUNC_6663	7	GTAG	2041
46712	47973	+	JUNC_6661	7	GTAG	1261
93045	94160	-	JUNC_15488	7	CTGC	1115
41652	42184	+	JUNC_5848	7	GTAG	532
51134	51644	-	JUNC_7382	7	CTGC	510
134786	135293	+	JUNC_23433	7	GTAG	507
1774	2168	+	JUNC_114	7	GTAG	394
28070	28456	+	JUNC_3420	7	GTAG	386
101681	101790	+	JUNC_17965	7	GCAG	109
30840	134990	+	JUNC_3645	6	GTAG	104150
38929	93768	+	JUNC_5184	6	GTAG	54839
92922	140362	-	JUNC_15412	6	CTAC	47440
12377	33581	+	JUNC_1580	6	GTAG	21204
35688	52302	+	JUNC_4204	6	GTAG	16614
44220	46578	+	JUNC_6236	6	GTAG	2358
109052	111003	+	JUNC_20460	6	GTAG	1951
28566	29957	+	JUNC_3454	6	GTAG	1391
45684	46617	+	JUNC_6471	6	GTAG	933
134587	135293	+	JUNC_23382	6	GTAG	706
121466	121973	-	JUNC_21721	6	CTGC	507
4268	4775	+	JUNC_563	6	GCAG	507
97223	97710	-	JUNC_16545	6	CTAC	487

132889	133073	-	JUNC_22852	6	CTAC	184
132903	133082	-	JUNC_22862	6	CTAC	179
52375	135218	-	JUNC_7902	5	CTAC	82843
60084	140285	-	JUNC_10169	5	CTAC	80201
51739	115987	-	JUNC_7557	5	CTAC	64248
103944	140237	+	JUNC_18581	5	GTAG	36293
106197	133238	+	JUNC_19590	5	GTAG	27041
12137	33581	+	JUNC_1549	5	GTAG	21444
109218	123950	-	JUNC_20490	5	CTAC	14732
97695	104805	+	JUNC_16715	5	GTAG	7110
91472	97867	+	JUNC_15181	5	GTAG	6395
132232	135293	+	JUNC_22661	5	GTAG	3061
98758	99965	-	JUNC_17082	5	CTAC	1207
45984	46578	+	JUNC_6529	5	GTAG	594
52208	52436	-	JUNC_7797	5	CTGC	228
79818	80026	+	JUNC_13057	5	GTAG	208
105899	106074	-	JUNC_19476	5	CTAC	175
145454	145625	-	JUNC_26195	5	CTAC	171
106394	106487	+	JUNC_19700	5	GTAG	93
68321	68406	+	JUNC_11895	5	GCAG	85
92048	92127	+	JUNC_15273	5	GCAG	79
88525	88602	+	JUNC_14443	5	GTAG	77
123060	123124	-	JUNC_21961	5	CTGC	64
3117	3181	+	JUNC_335	5	GCAG	64
97974	98032	-	JUNC_16829	5	CTGC	58
60591	60646	+	JUNC_10365	5	GTAG	55
23936	140279	-	JUNC_2569	4	CTAC	116343
24044	140300	-	JUNC_2599	4	CTAC	116256
28803	144249	+	JUNC_3475	4	GTAG	115446
53483	140285	-	JUNC_8305	4	CTAC	86802
2291	81949	+	JUNC_224	4	GTAG	79658
52375	121710	-	JUNC_7901	4	CTAC	69335
52383	118461	-	JUNC_7909	4	CTAC	66078
63503	121336	+	JUNC_11076	4	GTAG	57833

12397	58921	+	JUNC_1585	4	GCAG	46524
61569	107386	-	JUNC_10699	4	CTGC	45817
94124	139170	-	JUNC_15933	4	CTAC	45046
51643	94282	-	JUNC_7484	4	CTGC	42639
53504	88128	+	JUNC_8322	4	GTAG	34624
106394	132629	+	JUNC_19701	4	GTAG	26235
35510	61690	+	JUNC_4127	4	GTAG	26180
5557	31296	+	JUNC_806	4	GTAG	25739
26172	48457	-	JUNC_3094	4	CTAC	22285
2291	21993	+	JUNC_220	4	GTAG	19702
132191	138605	-	JUNC_22632	4	CTGC	6414
80961	83293	-	JUNC_13162	4	CTAC	2332
145454	146810	-	JUNC_26196	4	CTAC	1356
87068	88335	-	JUNC_14083	4	CTAC	1267
98799	99952	+	JUNC_17107	4	GTAG	1153
9253	9770	+	JUNC_963	4	GTAG	517
47499	48016	+	JUNC_6922	4	GTAG	517
139846	140303	-	JUNC_24536	4	CTAC	457
28070	28420	+	JUNC_3419	4	GTAG	350
55077	55315	+	JUNC_9084	4	GTAG	238
55538	55773	+	JUNC_9294	4	GTAG	235
56994	57229	+	JUNC_9505	4	GTAG	235
4930	5111	-	JUNC_671	4	CTAC	181
39469	39611	+	JUNC_5347	4	GTAG	142
35456	35595	+	JUNC_4098	4	GTAG	139
104858	104988	-	JUNC_19063	4	CTAC	130
79896	80023	+	JUNC_13063	4	GTAG	127
83310	83435	+	JUNC_13476	4	GCAG	125
107360	107448	-	JUNC_20066	4	CTAC	88
61051	61139	+	JUNC_10510	4	GCAG	88
6044	134965	+	JUNC_852	3	GTAG	128921
20962	139989	-	JUNC_2288	3	CTAC	119027
47637	139106	-	JUNC_6958	3	CTAC	91469
17488	108764	-	JUNC_2041	3	CTAC	91276

3599	91454	-	JUNC_451	3	CTAC	87855
53483	140303	-	JUNC_8306	3	CTAC	86820
59964	144445	+	JUNC_10121	3	GTAG	84481
38191	121375	+	JUNC_4958	3	GTAG	83184
59349	132704	+	JUNC_9882	3	GTAG	73355
26164	98252	+	JUNC_3090	3	GTAG	72088
36457	103945	+	JUNC_4471	3	GTAG	67488
3711	69973	-	JUNC_487	3	CTAC	66262
74190	140291	-	JUNC_12740	3	CTAC	66101
78432	139833	+	JUNC_12983	3	GTAG	61401
17208	72779	-	JUNC_2012	3	CTAC	55571
51981	106198	+	JUNC_7709	3	GTAG	54217
54749	99835	+	JUNC_8927	3	GTAG	45086
96500	140285	-	JUNC_16327	3	CTAC	43785
54956	98736	-	JUNC_9030	3	CTGC	43780
59094	100396	+	JUNC_9803	3	GTAG	41302
60444	100096	+	JUNC_10327	3	GCAG	39652
54453	93683	-	JUNC_8761	3	CTAC	39230
9161	47270	+	JUNC_953	3	GTAG	38109
86820	120684	-	JUNC_14045	3	CTAC	33864
99352	132419	+	JUNC_17357	3	GCAG	33067
83890	116702	+	JUNC_13566	3	GTAG	32812
61464	89972	+	JUNC_10652	3	GTAG	28508
77766	106205	+	JUNC_12937	3	GTAG	28439
25681	52557	+	JUNC_2960	3	GCAG	26876
26666	52293	+	JUNC_3219	3	GTAG	25627
31130	54513	+	JUNC_3688	3	GTAG	23383
123186	145625	-	JUNC_22014	3	CTAC	22439
31130	53505	+	JUNC_3687	3	GTAG	22375
109761	132010	-	JUNC_20526	3	CTAC	22249
98550	116410	+	JUNC_17032	3	GTAG	17860
132903	147018	-	JUNC_22863	3	CTAC	14115
51947	65019	+	JUNC_7691	3	GTAG	13072
104370	114025	+	JUNC_18824	3	GTAG	9655

85877	94291	-	JUNC_13868	3	CTAC	8414
123754	131786	-	JUNC_22079	3	CTAC	8032
26460	33581	+	JUNC_3165	3	GTAG	7121
54749	61594	+	JUNC_8922	3	GTAG	6845
63751	70515	-	JUNC_11117	3	CTGC	6764
26995	33581	+	JUNC_3287	3	GTAG	6586
27635	33581	+	JUNC_3377	3	GTAG	5946
28566	33581	+	JUNC_3455	3	GTAG	5015
35594	40575	+	JUNC_4161	3	GTAG	4981
123186	126622	-	JUNC_22012	3	CTAC	3436
124046	126343	-	JUNC_22120	3	CTAC	2297
99297	100972	-	JUNC_17341	3	CTGC	1675
124046	125650	-	JUNC_22119	3	CTAC	1604
591	2195	+	JUNC_25	3	GTAG	1604
137670	139272	-	JUNC_23922	3	CTAC	1602
133761	135293	+	JUNC_23209	3	GTAG	1532
91042	92456	+	JUNC_15104	3	GTAG	1414
139406	140779	-	JUNC_24368	3	CTGC	1373
4930	6010	-	JUNC_672	3	CTAC	1080
120231	121311	+	JUNC_21493	3	GTAG	1080
2268	3154	-	JUNC_207	3	CTAC	886
123087	123973	+	JUNC_21976	3	GTAG	886
104751	105580	+	JUNC_19008	3	GTAG	829
134587	135198	+	JUNC_23381	3	GTAG	611
12384	12921	+	JUNC_1582	3	GTAG	537
134786	135198	+	JUNC_23432	3	GTAG	412
113349	113709	+	JUNC_20892	3	GTAG	360
90474	90811	-	JUNC_14993	3	CTAC	337
108268	108517	-	JUNC_20295	3	CTGC	249
53115	53356	+	JUNC_8119	3	GCAG	241
139616	139844	-	JUNC_24417	3	CTAC	228
119352	119551	+	JUNC_21470	3	GTAG	199
121130	121311	+	JUNC_21623	3	GTAG	181
132232	132403	+	JUNC_22660	3	GTAG	171

38380	38549	-	JUNC_5017	3	CTGC	169
138918	139058	+	JUNC_24217	3	GTAG	140
10285	10421	-	JUNC_1243	3	CTAC	136
87539	87674	-	JUNC_14199	3	CTAC	135
61021	61139	+	JUNC_10502	3	GCAG	118
61609	61724	+	JUNC_10725	3	GTAG	115
106197	106309	+	JUNC_19587	3	GTAG	112
97641	97750	+	JUNC_16696	3	GTAG	109
38753	38861	+	JUNC_5125	3	GTAG	108
15339	15442	+	JUNC_1835	3	GCAG	103
38662	38754	+	JUNC_5108	3	GTAG	92
61662	61744	+	JUNC_10750	3	GTAG	82
128777	128858	-	JUNC_22355	3	CTGC	81
148999	149080	+	JUNC_26475	3	GCAG	81
15333	15403	-	JUNC_1832	3	CTAC	70
11095	11148	-	JUNC_1423	3	CTAC	53
3093	139133	-	JUNC_320	2	CTAC	136040
3633	139286	-	JUNC_464	2	CTAC	135653
8744	140645	-	JUNC_947	2	CTAC	131901
19742	136298	+	JUNC_2203	2	GTAG	116556
26460	142689	+	JUNC_3166	2	GTAG	116229
4233	120197	-	JUNC_559	2	CTAC	115964
6044	122008	+	JUNC_851	2	GTAG	115964
25446	140285	-	JUNC_2891	2	CTAC	114839
20524	134965	+	JUNC_2256	2	GTAG	114441
26689	139547	+	JUNC_3227	2	GTAG	112858
25689	133767	+	JUNC_2965	2	GTAG	108078
25884	130828	+	JUNC_3026	2	GTAG	104944
35022	138477	+	JUNC_3926	2	GTAG	103455
30275	132824	-	JUNC_3569	2	CTAC	102549
38398	139833	+	JUNC_5037	2	GTAG	101435
38753	138477	+	JUNC_5128	2	GTAG	99724
33479	130897	+	JUNC_3852	2	GTAG	97418
5073	101327	-	JUNC_705	2	CTAC	96254

47820	144021	+	JUNC_7006	2	GTAG	96201
596	96459	-	JUNC_29	2	CTAC	95863
38380	132824	-	JUNC_5023	2	CTAC	94444
1774	93725	+	JUNC_117	2	GTAG	91951
25698	116882	+	JUNC_2972	2	GTAG	91184
35022	125391	+	JUNC_3925	2	GTAG	90369
48367	138299	+	JUNC_7051	2	GTAG	89932
51827	140960	+	JUNC_7610	2	GTAG	89133
12402	97168	+	JUNC_1589	2	GTAG	84766
39469	123148	+	JUNC_5349	2	GTAG	83679
58626	140285	-	JUNC_9635	2	CTAC	81659
53888	134824	-	JUNC_8529	2	CTAC	80936
8208	87255	+	JUNC_931	2	GTAG	79047
52007	129220	+	JUNC_7720	2	GTAG	77213
55220	132161	-	JUNC_9153	2	CTAC	76941
66035	142537	-	JUNC_11454	2	CTAC	76502
72021	147545	+	JUNC_12574	2	GTAG	75524
59349	133348	+	JUNC_9883	2	GTAG	73999
58470	132400	+	JUNC_9588	2	GTAG	73930
55592	129421	+	JUNC_9315	2	GTAG	73829
26638	100041	-	JUNC_3211	2	CTAC	73403
61593	133439	+	JUNC_10712	2	GTAG	71846
26500	97246	+	JUNC_3180	2	GTAG	70746
44339	114025	+	JUNC_6260	2	GTAG	69686
66585	134861	+	JUNC_11545	2	GTAG	68276
25884	93879	+	JUNC_3023	2	GTAG	67995
79390	145617	+	JUNC_13040	2	GTAG	66227
74190	140282	-	JUNC_12739	2	CTAC	66092
66417	132407	-	JUNC_11510	2	CTAC	65990
52413	116187	-	JUNC_7928	2	CTAC	63774
36470	97642	+	JUNC_4474	2	GTAG	61172
44958	105693	+	JUNC_6358	2	GTAG	60735
1436	61594	+	JUNC_79	2	GTAG	60158
54947	114025	+	JUNC_9022	2	GTAG	59078

45711	103121	+	JUNC_6473	2	GTAG	57410
58626	113603	-	JUNC_9634	2	CTAC	54977
91374	146163	-	JUNC_15159	2	CTAC	54789
54243	109027	+	JUNC_8654	2	GTAG	54784
86624	141319	+	JUNC_14014	2	GTAG	54695
31310	84926	+	JUNC_3710	2	GTAG	53616
61723	113758	-	JUNC_10786	2	CTAC	52035
37171	88923	+	JUNC_4683	2	GTAG	51752
60834	112521	-	JUNC_10447	2	CTAC	51687
55500	106976	+	JUNC_9277	2	GTAG	51476
37567	88971	+	JUNC_4794	2	GTAG	51404
34252	84857	-	JUNC_3896	2	CTAC	50605
54297	104820	+	JUNC_8684	2	GTAG	50523
46654	96399	+	JUNC_6644	2	GTAG	49745
83685	133292	+	JUNC_13545	2	GTAG	49607
20962	70462	-	JUNC_2287	2	CTAC	49500
54512	103743	+	JUNC_8799	2	GTAG	49231
38951	88128	+	JUNC_5190	2	GCAG	49177
83985	132192	-	JUNC_13587	2	CTGC	48207
89866	137721	-	JUNC_14790	2	CTAC	47855
36964	84733	-	JUNC_4603	2	CTAC	47769
58502	106022	+	JUNC_9597	2	GTAG	47520
10555	57890	+	JUNC_1315	2	GTAG	47335
74880	122008	+	JUNC_12798	2	GCAG	47128
12402	58921	+	JUNC_1588	2	GTAG	46519
89651	135251	-	JUNC_14721	2	CTAC	45600
3711	49283	-	JUNC_485	2	CTAC	45572
98540	143431	289	JUNC_17030	2	GCAG	44891
45684	88971	+	JUNC_6472	2	GTAG	43287
61671	104865	-	JUNC_10760	2	CTGC	43194
47476	90475	-	JUNC_6912	2	CTAC	42999
61464	104394	+	JUNC_10653	2	GTAG	42930
71757	113887	-	JUNC_12553	2	CTAC	42130
55230	96928	+	JUNC_9160	2	GTAG	41698

91166	132770	-	JUNC_15121	2	CTAC	41604
60645	102158	+	JUNC_10384	2	GTAG	41513
10008	51044	-	JUNC_1172	2	CTGC	41036
101347	142032	+	JUNC_17909	2	GTAG	40685
54821	94046	-	JUNC_8956	2	CTGC	39225
54749	93578	+	JUNC_8925	2	GTAG	38829
8420	47168	+	JUNC_939	2	GTAG	38748
55788	94236	+	JUNC_9366	2	GTAG	38448
23741	61663	+	JUNC_2535	2	GTAG	37922
4233	41662	-	JUNC_557	2	CTAC	37429
21794	59201	-	JUNC_2323	2	CTAC	37407
59946	97168	+	JUNC_10110	2	GTAG	37222
104564	141328	+	JUNC_18920	2	GCAG	36764
61446	97784	-	JUNC_10642	2	CTAC	36338
103826	139997	-	JUNC_18495	2	CTGC	36171
11732	47276	+	JUNC_1513	2	GTAG	35544
54453	89990	-	JUNC_8760	2	CTAC	35537
52343	87336	+	JUNC_7882	2	GCAG	34993
70237	104851	+	JUNC_12220	2	GTAG	34614
55037	89157	-	JUNC_9068	2	CTAC	34120
30635	64698	+	JUNC_3618	2	GTAG	34063
106394	140051	+	JUNC_19702	2	GTAG	33657
98852	132344	+	JUNC_17144	2	GTAG	33492
59946	93423	+	JUNC_10107	2	GTAG	33477
98852	132322	+	JUNC_17143	2	GTAG	33470
98852	132300	+	JUNC_17142	2	GTAG	33448
98852	132278	+	JUNC_17141	2	GTAG	33426
98852	132256	+	JUNC_17140	2	GTAG	33404
99811	133037	+	JUNC_17485	2	GCAG	33226
8371	41042	-	JUNC_937	2	CTAC	32671
64894	96688	+	JUNC_11290	2	GTAG	31794
61593	93141	+	JUNC_10710	2	GTAG	31548
23407	54513	+	JUNC_2474	2	GTAG	31106
66919	97642	+	JUNC_11657	2	GTAG	30723

106810	136806	-	JUNC_19828	2	CTAC	29996
54749	84433	+	JUNC_8924	2	GTAG	29684
47153	76720	+	JUNC_6785	2	GTAG	29567
30756	60094	+	JUNC_3628	2	GTAG	29338
116432	145313	-	JUNC_21342	2	CTAC	28881
35660	64203	+	JUNC_4188	2	GTAG	28543
26592	54984	+	JUNC_3200	2	GTAG	28392
77058	104979	+	JUNC_12904	2	GTAG	27921
58626	86490	-	JUNC_9632	2	CTAC	27864
36712	64203	+	JUNC_4530	2	GTAG	27491
25422	52557	+	JUNC_2880	2	GTAG	27135
12791	39911	+	JUNC_1625	2	GTAG	27120
35723	62642	+	JUNC_4229	2	GCAG	26919
105899	132192	-	JUNC_19477	2	CTGC	26293
70498	96751	-	JUNC_12309	2	CTAC	26253
36113	61960	-	JUNC_4350	2	CTAC	25847
26736	52557	+	JUNC_3236	2	GTAG	25821
107294	132890	-	JUNC_20037	2	CTAC	25596
58915	84042	+	JUNC_9733	2	GCAG	25127
68261	93111	+	JUNC_11888	2	GTAG	24850
40258	64275	-	JUNC_5584	2	CTAC	24017
70498	94416	-	JUNC_12308	2	CTAC	23918
38110	61672	-	JUNC_4921	2	CTGC	23562
69620	92958	-	JUNC_12025	2	CTAC	23338
97108	120192	+	JUNC_16505	2	GTAG	23084
47476	70542	-	JUNC_6910	2	CTAC	23066
90460	113505	-	JUNC_14989	2	CTAC	23045
44220	66622	+	JUNC_6237	2	GTAG	22402
74847	97246	+	JUNC_12795	2	GTAG	22399
39529	61846	+	JUNC_5366	2	GTAG	22317
35677	57589	-	JUNC_4199	2	CTAC	21912
66648	88218	+	JUNC_11556	2	GTAG	21570
40060	61372	-	JUNC_5512	2	CTAC	21312
5182	26461	+	JUNC_726	2	GTAG	21279

31523	52247	+	JUNC_3742	2	GTAG	20724
13847	33581	+	JUNC_1717	2	GTAG	19734
53558	73273	+	JUNC_8355	2	GTAG	19715
90411	109845	-	JUNC_14965	2	CTAC	19434
112579	132003	-	JUNC_20802	2	CTAC	19424
78039	97291	-	JUNC_12955	2	CTAC	19252
35594	54750	+	JUNC_4162	2	GTAG	19156
122964	140362	-	JUNC_21930	2	CTAC	17398
37171	54513	+	JUNC_4681	2	GTAG	17342
53504	70539	+	JUNC_8321	2	GTAG	17035
123447	140285	-	JUNC_22039	2	CTAC	16838
39415	55882	-	JUNC_5330	2	CTAC	16467
107421	123670	-	JUNC_20101	2	CTAC	16249
126696	142870	-	JUNC_22287	2	CTAC	16174
36160	52110	+	JUNC_4370	2	GTAG	15950
74077	89915	-	JUNC_12727	2	CTAC	15838
36253	52041	+	JUNC_4397	2	GCAG	15788
10848	26461	+	JUNC_1371	2	GTAG	15613
2213	17480	-	JUNC_187	2	CTAC	15267
31004	46267	-	JUNC_3675	2	CTAC	15263
3219	18348	-	JUNC_358	2	CTAC	15129
54749	69811	+	JUNC_8923	2	GTAG	15062
40574	54513	+	JUNC_5670	2	GTAG	13939
87539	100760	-	JUNC_14200	2	CTAC	13221
91219	104337	+	JUNC_15126	2	GTAG	13118
91543	104091	+	JUNC_15200	2	GTAG	12548
120330	132704	+	JUNC_21494	2	GTAG	12374
55788	68075	+	JUNC_9363	2	GTAG	12287
58626	70636	-	JUNC_9631	2	CTAC	12010
36249	47154	+	JUNC_4395	2	GTAG	10905
48878	58576	-	JUNC_7113	2	CTAC	9698
42545	52230	-	JUNC_6017	2	CTAC	9685
98857	106407	+	JUNC_17147	2	GTAG	7550
107756	115045	-	JUNC_20184	2	CTAC	7289

60162	66910	-	JUNC_10208	2	CTGC	6748
26914	33581	+	JUNC_3270	2	GTAG	6667
91543	98064	+	JUNC_15198	2	GTAG	6521
92252	98743	+	JUNC_15293	2	GTAG	6491
54410	60646	+	JUNC_8732	2	GTAG	6236
35249	41381	-	JUNC_4007	2	CTGC	6132
67021	73013	+	JUNC_11696	2	GTAG	5992
27751	33581	+	JUNC_3395	2	GTAG	5830
61609	67295	+	JUNC_10726	2	GTAG	5686
28070	33581	+	JUNC_3421	2	GTAG	5511
127173	132629	+	JUNC_22291	2	GTAG	5456
83052	88100	-	JUNC_13425	2	CTAC	5048
93260	98195	+	JUNC_15587	2	GTAG	4935
80961	85696	-	JUNC_13163	2	CTAC	4735
29033	33581	+	JUNC_3491	2	GTAG	4548
10243	14520	-	JUNC_1227	2	CTAC	4277
100560	104433	+	JUNC_17707	2	GTAG	3873
35510	39209	+	JUNC_4124	2	GTAG	3699
55332	58921	+	JUNC_9206	2	GTAG	3589
104564	107997	+	JUNC_18917	2	GCAG	3433
104012	107333	-	JUNC_18619	2	CTAC	3321
86667	89957	-	JUNC_14021	2	CTAC	3290
131943	134995	+	JUNC_22514	2	GTAG	3052
6722	9770	+	JUNC_874	2	GTAG	3048
105692	108604	+	JUNC_19397	2	GCAG	2912
105421	108267	+	JUNC_19276	2	GTAG	2846
134587	137261	+	JUNC_23383	2	GTAG	2674
136790	139267	-	JUNC_23735	2	CTGC	2477
104037	106395	+	JUNC_18643	2	GTAG	2358
104204	106528	-	JUNC_18743	2	CTAC	2324
101134	103393	+	JUNC_17859	2	GTAG	2259
96205	97914	-	JUNC_16220	2	CTGC	1709
135139	136848	+	JUNC_23525	2	GTAG	1709
140783	142410	-	JUNC_24904	2	CTAC	1627

98791	100393	+	JUNC_17104	2	GTAG	1602
143858	145406	+	JUNC_25512	2	GTAG	1548
62215	63594	-	JUNC_10949	2	CTAC	1379
92997	94301	-	JUNC_15467	2	CTAC	1304
98857	100051	+	JUNC_17146	2	GTAG	1194
72611	73682	+	JUNC_12618	2	GTAG	1071
89030	89990	-	JUNC_14570	2	CTAC	960
145868	146814	-	JUNC_26333	2	CTAC	946
131043	131989	+	JUNC_22455	2	GTAG	946
106643	107526	+	JUNC_19791	2	GCAG	883
93191	94069	-	JUNC_15556	2	CTGC	878
104763	105580	+	JUNC_19011	2	GTAG	817
32858	33581	+	JUNC_3822	2	GTAG	723
128777	129490	-	JUNC_22356	2	CTAC	713
148367	149080	+	JUNC_26470	2	GTAG	713
124046	124741	-	JUNC_22118	2	CTAC	695
1500	2195	+	JUNC_81	2	GTAG	695
36610	37253	+	JUNC_4512	2	GCAG	643
45984	46617	+	JUNC_6530	2	GTAG	633
41652	42252	+	JUNC_5850	2	GTAG	600
29009	29604	-	JUNC_3488	2	CTAC	595
134556	135070	-	JUNC_23371	2	CTAC	514
51149	51644	-	JUNC_7384	2	CTGC	495
40146	40612	+	JUNC_5547	2	GCAG	466
54243	54649	+	JUNC_8650	2	GTAG	406
89549	89915	-	JUNC_14690	2	CTAC	366
51291	51644	-	JUNC_7403	2	CTGC	353
102586	102933	-	JUNC_18182	2	CTAC	347
28070	28417	+	JUNC_3418	2	GTAG	347
96693	97024	-	JUNC_16369	2	CTGC	331
114033	114351	-	JUNC_21013	2	CTGC	318
55495	55789	+	JUNC_9274	2	GTAG	294
87496	87783	+	JUNC_14189	2	GCAG	287
102600	102886	+	JUNC_18187	2	GCAG	286

99297	99581	-	JUNC_17340	2	CTAC	284
3093	3376	-	JUNC_318	2	CTAC	283
122865	123148	+	JUNC_21911	2	GTAG	283
47890	48166	-	JUNC_7011	2	CTAC	276
64435	64702	-	JUNC_11247	2	CTGC	267
69613	69868	+	JUNC_12021	2	GTAG	255
114109	114364	+	JUNC_21036	2	GTAG	255
51929	52176	+	JUNC_7675	2	GCAG	247
58751	58981	+	JUNC_9678	2	GCAG	230
87647	87861	+	JUNC_14230	2	GTAG	214
4614	4816	-	JUNC_580	2	CTGC	202
121425	121627	+	JUNC_21700	2	GCAG	202
96314	96501	-	JUNC_16273	2	CTAC	187
55251	55437	+	JUNC_9167	2	GTAG	186
53225	53409	-	JUNC_8173	2	CTGC	184
45133	45310	+	JUNC_6394	2	GCAG	177
47254	47426	+	JUNC_6828	2	GCAG	172
104222	104394	+	JUNC_18753	2	GTAG	172
89651	89822	-	JUNC_14720	2	CTGC	171
56994	57164	+	JUNC_9504	2	GTAG	170
96197	96364	+	JUNC_16218	2	GCAG	167
108836	109002	+	JUNC_20408	2	GCAG	166
133180	133345	-	JUNC_22982	2	CTGC	165
99808	99970	+	JUNC_17482	2	GTAG	162
34453	34606	-	JUNC_3906	2	CTAC	153
75906	76057	+	JUNC_12841	2	GCAG	151
53234	53376	+	JUNC_8180	2	GTAG	142
5401	5541	-	JUNC_771	2	CTGC	140
120700	120840	+	JUNC_21513	2	GCAG	140
53237	53376	+	JUNC_8183	2	GCAG	139
61698	61834	+	JUNC_10776	2	GCAG	136
60834	60965	-	JUNC_10444	2	CTAC	131
79896	80026	+	JUNC_13064	2	GTAG	130
89800	89927	+	JUNC_14766	2	GCAG	127

142968	143082	-	JUNC_25355	2	CTGC	114
138918	139032	+	JUNC_24216	2	GTAG	114
51929	52041	+	JUNC_7673	2	GCAG	112
75546	75652	+	JUNC_12822	2	GCAG	106
108338	108440	+	JUNC_20308	2	GCAG	102
133342	133439	+	JUNC_23051	2	GCAG	97
53504	53598	+	JUNC_8319	2	GTAG	94
94995	95086	-	JUNC_16127	2	CTAC	91
105985	106074	-	JUNC_19521	2	CTAC	89
51007	51096	-	JUNC_7367	2	CTGC	89
4905	4992	-	JUNC_654	2	CTGC	87
121249	121336	+	JUNC_21648	2	GCAG	87
92979	93065	-	JUNC_15451	2	CTGC	86
60133	60214	-	JUNC_10198	2	CTGC	81
4792	4867	-	JUNC_608	2	CTAC	75
121374	121449	+	JUNC_21685	2	GTAG	75
5065	5138	-	JUNC_703	2	CTGC	73
60168	60241	+	JUNC_10214	2	GTAG	73
16890	16961	-	JUNC_1985	2	CTGC	71
65592	65663	+	JUNC_11382	2	GTAG	71
3389	3453	-	JUNC_394	2	CTAC	64
122788	122852	+	JUNC_21892	2	GTAG	64
87629	87683	-	JUNC_14223	2	CTGC	54
36418	36471	+	JUNC_4451	2	GTAG	53
15367	15418	+	JUNC_1840	2	GCAG	51
5230	149616	+	JUNC_737	1	GTAG	144386
4930	145625	-	JUNC_676	1	CTAC	140695
596	141234	-	JUNC_30	1	CTAC	140638
869	140291	-	JUNC_52	1	CTAC	139422
3711	141844	-	JUNC_488	1	CTAC	138133
3389	140303	-	JUNC_397	1	CTAC	136914
2176	138798	-	JUNC_177	1	CTAC	136622
4824	140291	-	JUNC_623	1	CTAC	135467
4824	140279	-	JUNC_622	1	CTAC	135455

4830	140285	-	JUNC_632	1	CTAC	135455
2734	136860	+	JUNC_269	1	GTAG	134126
2906	136721	-	JUNC_287	1	CTAC	133815
6677	140332	-	JUNC_873	1	CTAC	133655
2291	135293	+	JUNC_225	1	GTAG	133002
4908	137153	-	JUNC_662	1	CTAC	132245
1641	132688	-	JUNC_91	1	CTAC	131047
9681	140356	-	JUNC_1089	1	CTAC	130675
10097	140645	-	JUNC_1190	1	CTAC	130548
6969	136852	+	JUNC_884	1	GTAG	129883
9161	138150	+	JUNC_954	1	GTAG	128989
6044	134995	+	JUNC_853	1	GTAG	128951
3375	132161	-	JUNC_393	1	CTAC	128786
14507	143110	+	JUNC_1767	1	GTAG	128603
4605	132970	-	JUNC_579	1	CTAC	128365
12402	140117	+	JUNC_1590	1	GTAG	127715
8169	135127	+	JUNC_930	1	GTAG	126958
17224	143234	+	JUNC_2016	1	GTAG	126010
11204	136001	+	JUNC_1449	1	GTAG	124797
7782	132015	+	JUNC_914	1	GTAG	124233
4538	128693	+	JUNC_573	1	GTAG	124155
26487	149496	+	JUNC_3175	1	GTAG	123009
1716	124474	-	JUNC_106	1	CTAC	122758
1767	124525	+	JUNC_112	1	GTAG	122758
11268	132890	-	JUNC_1459	1	CTAC	121622
23378	144396	+	JUNC_2468	1	GTAG	121018
23497	143816	-	JUNC_2492	1	CTAC	120319
24772	145076	-	JUNC_2671	1	CTAC	120304
16859	136887	+	JUNC_1982	1	GTAG	120028
20524	140495	+	JUNC_2258	1	GTAG	119971
18227	137004	-	JUNC_2124	1	CTAC	118777
23365	139928	-	JUNC_2462	1	CTAC	116563
24044	140279	-	JUNC_2598	1	CTAC	116235
2474	118619	-	JUNC_239	1	CTAC	116145

7622	123767	+	JUNC_894	1	GTAG	116145
24172	140285	-	JUNC_2618	1	CTAC	116113
30783	146119	+	JUNC_3635	1	GTAG	115336
25446	140282	-	JUNC_2890	1	CTAC	114836
25560	140285	-	JUNC_2922	1	CTAC	114725
20524	134995	+	JUNC_2257	1	GTAG	114471
27635	141940	+	JUNC_3381	1	GTAG	114305
23972	138147	+	JUNC_2583	1	GTAG	114175
6400	120456	-	JUNC_866	1	CTAC	114056
5785	119841	+	JUNC_832	1	GTAG	114056
6400	120440	-	JUNC_865	1	CTAC	114040
5801	119841	+	JUNC_834	1	GTAG	114040
6400	120424	-	JUNC_864	1	CTAC	114024
5817	119841	+	JUNC_836	1	GTAG	114024
6400	120408	-	JUNC_863	1	CTAC	114008
5833	119841	+	JUNC_838	1	GTAG	114008
6400	120392	-	JUNC_862	1	CTAC	113992
5849	119841	+	JUNC_842	1	GTAG	113992
4186	117680	-	JUNC_548	1	CTAC	113494
8561	122055	+	JUNC_943	1	GTAG	113494
19430	132704	+	JUNC_2187	1	GTAG	113274
25884	139065	+	JUNC_3028	1	GTAG	113181
37261	150147	-	JUNC_4720	1	CTAC	112886
33570	145625	-	JUNC_3856	1	CTAC	112055
31310	143272	+	JUNC_3713	1	GTAG	111962
40144	151496	+	JUNC_5545	1	GTAG	111352
22642	133252	-	JUNC_2369	1	CTAC	110610
37084	147357	+	JUNC_4649	1	GTAG	110273
29525	139089	+	JUNC_3515	1	GTAG	109564
12407	121411	+	JUNC_1592	1	GTAG	109004
38380	147030	-	JUNC_5024	1	CTAC	108650
24482	132787	-	JUNC_2658	1	CTAC	108305
31004	139286	-	JUNC_3677	1	CTAC	108282
27298	134995	+	JUNC_3317	1	GTAG	107697

25884	133439	+	JUNC_3027	1	GTAG	107555
35249	142783	-	JUNC_4013	1	CTAC	107534
4898	112238	-	JUNC_650	1	CTAC	107340
11899	118675	+	JUNC_1529	1	GTAG	106776
26530	132629	+	JUNC_3188	1	GTAG	106099
39427	145337	-	JUNC_5332	1	CTAC	105910
37237	143133	-	JUNC_4714	1	CTAC	105896
30689	136577	-	JUNC_3623	1	CTAC	105888
37706	142793	+	JUNC_4829	1	GTAG	105087
15876	120823	-	JUNC_1886	1	CTAC	104947
30174	134824	-	JUNC_3554	1	CTAC	104650
46690	150855	+	JUNC_6653	1	GTAG	104165
30840	134736	+	JUNC_3644	1	GTAG	103896
16964	120197	-	JUNC_1991	1	CTAC	103233
37036	140152	+	JUNC_4632	1	GTAG	103116
1439	104247	+	JUNC_80	1	GTAG	102808
30096	132711	+	JUNC_3547	1	GTAG	102615
36470	138557	+	JUNC_4477	1	GTAG	102087
2988	104835	-	JUNC_301	1	CTAC	101847
11586	113230	+	JUNC_1498	1	GTAG	101644
37036	138557	+	JUNC_4631	1	GTAG	101521
30932	132161	-	JUNC_3659	1	CTAC	101229
16964	118114	-	JUNC_1990	1	CTAC	101150
8169	109002	+	JUNC_929	1	GTAG	100833
4584	105301	-	JUNC_575	1	CTAC	100717
32018	132424	-	JUNC_3779	1	CTAC	100406
40258	140300	-	JUNC_5585	1	CTAC	100042
39901	139593	-	JUNC_5464	1	CTAC	99692
37237	136910	-	JUNC_4713	1	CTAC	99673
44628	144249	+	JUNC_6320	1	GTAG	99621
36111	134990	+	JUNC_4348	1	GTAG	98879
50150	148989	-	JUNC_7248	1	CTAC	98839
36470	135293	+	JUNC_4476	1	GTAG	98823
8214	106246	+	JUNC_932	1	GTAG	98032

25114	123068	+	JUNC_2769	1	GTAG	97954
1251	98965	-	JUNC_68	1	CTAC	97714
16542	114139	-	JUNC_1953	1	CTAC	97597
35471	132629	+	JUNC_4106	1	GTAG	97158
43369	140438	-	JUNC_6132	1	CTAC	97069
8276	105301	-	JUNC_935	1	CTAC	97025
42245	139265	-	JUNC_5968	1	CTAC	97020
1223	98224	-	JUNC_66	1	CTAC	97001
1050	97852	-	JUNC_61	1	CTAC	96802
5557	102318	+	JUNC_810	1	GTAG	96761
35240	131926	-	JUNC_4000	1	CTAC	96686
36948	133265	-	JUNC_4594	1	CTAC	96317
11086	107348	-	JUNC_1421	1	CTAC	96262
705	96501	-	JUNC_43	1	CTAC	95796
37706	133238	+	JUNC_4828	1	GTAG	95532
39529	134622	+	JUNC_5368	1	GTAG	95093
52383	147018	-	JUNC_7910	1	CTAC	94635
38758	133252	-	JUNC_5130	1	CTAC	94494
53675	148013	-	JUNC_8435	1	CTAC	94338
28455	122530	+	JUNC_3448	1	GTAG	94075
2372	96423	-	JUNC_233	1	CTAC	94051
55008	149037	+	JUNC_9056	1	GTAG	94029
5498	99463	+	JUNC_794	1	GTAG	93965
39469	133238	+	JUNC_5350	1	GTAG	93769
3495	96928	+	JUNC_430	1	GTAG	93433
3375	96785	-	JUNC_392	1	CTAC	93410
3483	96805	-	JUNC_424	1	CTAC	93322
37412	130085	+	JUNC_4758	1	GTAG	92673
59349	151940	+	JUNC_9884	1	GTAG	92591
35660	128182	+	JUNC_4189	1	GTAG	92522
46712	139122	+	JUNC_6665	1	GTAG	92410
23497	115691	-	JUNC_2491	1	CTAC	92194
1774	93777	+	JUNC_118	1	GTAG	92003
41441	133286	+	JUNC_5813	1	GTAG	91845

51278	142890	-	JUNC_7399	1	CTAC	91612
30245	121564	+	JUNC_3563	1	GTAG	91319
2268	93313	-	JUNC_208	1	CTAC	91045
37306	128182	+	JUNC_4732	1	GTAG	90876
7273	98115	-	JUNC_888	1	CTAC	90842
53894	144345	-	JUNC_8533	1	CTAC	90451
13646	104082	-	JUNC_1696	1	CTAC	90436
48092	138477	+	JUNC_7025	1	GTAG	90385
52431	142761	-	JUNC_7940	1	CTAC	90330
49562	139847	-	JUNC_7182	1	CTAC	90285
38645	128840	+	JUNC_5100	1	GTAG	90195
25092	115045	-	JUNC_2760	1	CTAC	89953
39311	128840	+	JUNC_5290	1	GTAG	89529
55788	144951	+	JUNC_9370	1	GTAG	89163
58756	147909	+	JUNC_9682	1	GTAG	89153
51475	140282	-	JUNC_7444	1	CTAC	88807
54113	142839	-	JUNC_8602	1	CTAC	88726
52413	140285	-	JUNC_7931	1	CTAC	87872
55989	143816	-	JUNC_9447	1	CTAC	87827
18293	106022	+	JUNC_2125	1	GTAG	87729
56490	144118	-	JUNC_9484	1	CTAC	87628
55047	142540	+	JUNC_9071	1	GTAG	87493
62058	149523	+	JUNC_10882	1	GTAG	87465
45629	133073	-	JUNC_6459	1	CTAC	87444
53210	140362	-	JUNC_8164	1	CTAC	87152
6815	93914	-	JUNC_878	1	CTAC	87099
58379	145400	-	JUNC_9566	1	CTAC	87021
36107	123068	+	JUNC_4343	1	GTAG	86961
53675	140285	-	JUNC_8434	1	CTAC	86610
11312	97773	-	JUNC_1467	1	CTAC	86461
46654	133037	+	JUNC_6645	1	GTAG	86383
10243	96501	-	JUNC_1229	1	CTAC	86258
55788	141876	+	JUNC_9369	1	GTAG	86088
53943	139830	-	JUNC_8552	1	CTAC	85887

55436	141319	+	JUNC_9253	1	GTAG	85883
35249	120928	-	JUNC_4012	1	CTAC	85679
54911	140332	-	JUNC_9007	1	CTAC	85421
54422	139830	-	JUNC_8738	1	CTAC	85408
54911	140291	-	JUNC_9006	1	CTAC	85380
46855	132183	-	JUNC_6708	1	CTAC	85328
54689	139928	-	JUNC_8885	1	CTAC	85239
55148	140282	-	JUNC_9118	1	CTAC	85134
55421	140342	-	JUNC_9243	1	CTAC	84921
50309	134995	+	JUNC_7281	1	GTAG	84686
50309	134965	+	JUNC_7280	1	GTAG	84656
9276	93764	-	JUNC_970	1	CTAC	84488
19523	103640	-	JUNC_2189	1	CTAC	84117
54243	138150	+	JUNC_8656	1	GTAG	83907
49562	133073	-	JUNC_7181	1	CTAC	83511
55772	138972	+	JUNC_9352	1	GTAG	83200
17224	100381	+	JUNC_2015	1	GTAG	83157
17224	100366	+	JUNC_2014	1	GTAG	83142
65258	148026	+	JUNC_11344	1	GTAG	82768
13267	96034	-	JUNC_1662	1	CTAC	82767
38443	121168	+	JUNC_5050	1	GTAG	82725
61689	144367	+	JUNC_10772	1	GTAG	82678
57903	140285	-	JUNC_9547	1	CTAC	82382
52768	134995	+	JUNC_8070	1	GTAG	82227
25884	107832	+	JUNC_3025	1	GTAG	81948
57760	139703	+	JUNC_9539	1	GTAG	81943
39013	120912	+	JUNC_5201	1	GTAG	81899
60804	142674	-	JUNC_10432	1	CTAC	81870
51638	133439	+	JUNC_7481	1	GTAG	81801
58695	140270	-	JUNC_9654	1	CTAC	81575
58865	140285	-	JUNC_9715	1	CTAC	81420
52026	133439	+	JUNC_7726	1	GTAG	81413
58250	139579	+	JUNC_9560	1	GTAG	81329
61256	142540	+	JUNC_10569	1	GTAG	81284

59211	140291	-	JUNC_9839	1	CTAC	81080
26283	106275	-	JUNC_3121	1	CTAC	79992
56901	136857	+	JUNC_9494	1	GTAG	79956
30635	110428	+	JUNC_3619	1	GTAG	79793
13880	93590	-	JUNC_1720	1	CTAC	79710
53606	133252	-	JUNC_8378	1	CTAC	79646
55022	134622	+	JUNC_9062	1	GTAG	79600
54075	133238	+	JUNC_8586	1	GTAG	79163
23769	102704	-	JUNC_2538	1	CTAC	78935
61197	140117	+	JUNC_10547	1	GTAG	78920
39407	118286	-	JUNC_5328	1	CTAC	78879
61425	140285	-	JUNC_10627	1	CTAC	78860
54453	133265	-	JUNC_8762	1	CTAC	78812
55412	134217	+	JUNC_9239	1	GTAG	78805
54377	133163	-	JUNC_8718	1	CTAC	78786
31004	109755	-	JUNC_3676	1	CTAC	78751
35249	113725	-	JUNC_4011	1	CTAC	78476
54983	133439	+	JUNC_9044	1	GTAG	78456
47098	125495	+	JUNC_6772	1	GTAG	78397
54332	132623	+	JUNC_8700	1	GTAG	78291
54911	133163	-	JUNC_9005	1	CTAC	78252
54911	133139	-	JUNC_9004	1	CTAC	78228
6366	84542	+	JUNC_861	1	GTAG	78176
3389	81445	-	JUNC_395	1	CTAC	78056
26382	104382	+	JUNC_3142	1	GTAG	78000
6528	84407	-	JUNC_868	1	CTAC	77879
25422	103111	+	JUNC_2881	1	GTAG	77689
39901	117526	-	JUNC_5463	1	CTAC	77625
62894	140362	-	JUNC_11015	1	CTAC	77468
54947	132400	+	JUNC_9023	1	GTAG	77453
55954	133343	+	JUNC_9437	1	GTAG	77389
13841	91205	-	JUNC_1716	1	CTAC	77364
54126	131048	+	JUNC_8609	1	GTAG	76922
63127	139939	-	JUNC_11038	1	CTAC	76812

54914	131094	-	JUNC_9010	1	CTAC	76180
38904	115045	-	JUNC_5175	1	CTAC	76141
61799	137042	-	JUNC_10805	1	CTAC	75243
23532	98743	+	JUNC_2500	1	GTAG	75211
23297	98176	-	JUNC_2449	1	CTAC	74879
66909	141593	-	JUNC_11654	1	CTAC	74684
19289	93893	-	JUNC_2182	1	CTAC	74604
51813	126179	-	JUNC_7604	1	CTAC	74366
62323	136682	-	JUNC_10961	1	CTAC	74359
39757	114025	+	JUNC_5417	1	GTAG	74268
38260	112386	+	JUNC_4976	1	GTAG	74126
26547	100670	+	JUNC_3190	1	GTAG	74123
65183	139089	+	JUNC_11335	1	GTAG	73906
54380	127855	+	JUNC_8721	1	GTAG	73475
61644	134980	-	JUNC_10747	1	CTAC	73336
68959	142215	-	JUNC_11946	1	CTAC	73256
31523	104752	+	JUNC_3743	1	GTAG	73229
23714	96760	+	JUNC_2533	1	GTAG	73046
66825	139788	+	JUNC_11625	1	GTAG	72963
39766	112728	-	JUNC_5423	1	CTAC	72962
33456	106385	+	JUNC_3850	1	GTAG	72929
61671	134470	-	JUNC_10761	1	CTAC	72799
69828	142573	-	JUNC_12091	1	CTAC	72745
71630	144073	+	JUNC_12546	1	GTAG	72443
8104	80311	+	JUNC_924	1	GTAG	72207
60516	132629	+	JUNC_10345	1	GTAG	72113
34104	106074	-	JUNC_3889	1	CTAC	71970
54914	126678	-	JUNC_9009	1	CTAC	71764
60549	132156	-	JUNC_10352	1	CTAC	71607
63383	134970	+	JUNC_11064	1	GTAG	71587
5623	77191	-	JUNC_828	1	CTAC	71568
35699	107085	+	JUNC_4214	1	GTAG	71386
35718	107085	+	JUNC_4223	1	GTAG	71367
42877	114025	+	JUNC_6062	1	GTAG	71148

25884	96928	+	JUNC_3024	1	GTAG	71044
36059	107085	+	JUNC_4330	1	GTAG	71026
70702	141708	+	JUNC_12422	1	GTAG	71006
71926	142811	+	JUNC_12565	1	GTAG	70885
61436	132161	-	JUNC_10637	1	CTAC	70725
71926	142495	+	JUNC_12564	1	GTAG	70569
59964	130451	+	JUNC_10120	1	GTAG	70487
35688	106010	+	JUNC_4207	1	GTAG	70322
23041	92917	-	JUNC_2407	1	CTAC	69876
815	70539	+	JUNC_50	1	GTAG	69724
27635	97246	+	JUNC_3380	1	GTAG	69611
77223	146679	+	JUNC_12918	1	GTAG	69456
40699	110104	+	JUNC_5735	1	GTAG	69405
54785	123973	+	JUNC_8943	1	GTAG	69188
35706	104818	+	JUNC_4217	1	GTAG	69112
25066	94098	-	JUNC_2751	1	CTAC	69032
36160	105094	+	JUNC_4372	1	GTAG	68934
24011	92922	-	JUNC_2589	1	CTAC	68911
51870	120684	-	JUNC_7642	1	CTAC	68814
59946	128693	+	JUNC_10112	1	GTAG	68747
35100	103726	+	JUNC_3950	1	GTAG	68626
37516	106074	-	JUNC_4785	1	CTAC	68558
21215	89724	-	JUNC_2299	1	CTAC	68509
25311	93728	-	JUNC_2851	1	CTAC	68417
35688	104091	+	JUNC_4206	1	GTAG	68403
25387	93390	-	JUNC_2870	1	CTAC	68003
36470	104454	+	JUNC_4475	1	GTAG	67984
6044	74014	+	JUNC_850	1	GTAG	67970
53298	121168	+	JUNC_8224	1	GTAG	67870
81245	148904	+	JUNC_13199	1	GTAG	67659
31310	98831	+	JUNC_3712	1	GTAG	67521
16964	84407	-	JUNC_1989	1	CTAC	67443
36712	103863	+	JUNC_4531	1	GTAG	67151
3375	70460	-	JUNC_391	1	CTAC	67085

54332	121375	+	JUNC_8699	1	GTAG	67043
30275	96829	-	JUNC_3567	1	CTAC	66554
71738	138147	+	JUNC_12550	1	GTAG	66409
9763	76000	+	JUNC_1107	1	GTAG	66237
25281	91463	+	JUNC_2843	1	GTAG	66182
79390	145356	+	JUNC_13039	1	GTAG	65966
38274	104223	+	JUNC_4982	1	GTAG	65949
26643	92456	+	JUNC_3213	1	GTAG	65813
40623	106385	+	JUNC_5694	1	GTAG	65762
38074	103743	+	JUNC_4909	1	GTAG	65669
31310	96928	+	JUNC_3711	1	GTAG	65618
67312	132733	+	JUNC_11792	1	GTAG	65421
61425	126190	-	JUNC_10626	1	CTAC	64765
80751	145418	-	JUNC_13124	1	CTAC	64667
25221	89787	-	JUNC_2815	1	CTAC	64566
60884	125044	+	JUNC_10465	1	GTAG	64160
40144	104005	+	JUNC_5544	1	GTAG	63861
37876	101639	+	JUNC_4874	1	GTAG	63763
60552	123887	-	JUNC_10353	1	CTAC	63335
61318	124574	+	JUNC_10590	1	GTAG	63256
44638	107832	+	JUNC_6323	1	GTAG	63194
3489	66649	+	JUNC_427	1	GTAG	63160
25737	88839	-	JUNC_2983	1	CTAC	63102
69972	133073	-	JUNC_12152	1	CTAC	63101
42214	105311	-	JUNC_5963	1	CTAC	63097
7780	70681	+	JUNC_911	1	GTAG	62901
40621	103482	-	JUNC_5693	1	CTAC	62861
36160	98933	+	JUNC_4371	1	GTAG	62773
40643	103111	+	JUNC_5707	1	GTAG	62468
71289	133429	-	JUNC_12518	1	CTAC	62140
35688	97750	+	JUNC_4205	1	GTAG	62062
42408	104394	+	JUNC_5997	1	GTAG	61986
67182	129130	+	JUNC_11758	1	GTAG	61948
71738	133556	+	JUNC_12549	1	GTAG	61818

4233	65799	-	JUNC_558	1	CTAC	61566
78069	139593	-	JUNC_12959	1	CTAC	61524
67011	128473	+	JUNC_11692	1	GTAG	61462
35718	96928	+	JUNC_4222	1	GTAG	61210
35769	96928	+	JUNC_4246	1	GTAG	61159
43568	104707	-	JUNC_6148	1	CTAC	61139
44110	105068	+	JUNC_6213	1	GTAG	60958
2213	63156	-	JUNC_188	1	CTAC	60943
84865	145400	-	JUNC_13735	1	CTAC	60535
29060	89352	-	JUNC_3494	1	CTAC	60292
53624	113698	-	JUNC_8393	1	CTAC	60074
72508	132474	+	JUNC_12610	1	GTAG	59966
49562	109382	-	JUNC_7180	1	CTAC	59820
7780	67449	+	JUNC_910	1	GTAG	59669
40036	99578	-	JUNC_5501	1	CTAC	59542
61446	120968	-	JUNC_10643	1	CTAC	59522
40153	99453	+	JUNC_5553	1	GTAG	59300
39649	98709	+	JUNC_5396	1	GTAG	59060
61959	121011	-	JUNC_10853	1	CTAC	59052
3251	62039	-	JUNC_366	1	CTAC	58788
54431	113188	-	JUNC_8746	1	CTAC	58757
48367	106883	+	JUNC_7050	1	GTAG	58516
38380	96768	-	JUNC_5022	1	CTAC	58388
24772	83135	-	JUNC_2670	1	CTAC	58363
55788	114025	+	JUNC_9368	1	GTAG	58237
35769	93738	+	JUNC_4245	1	GTAG	57969
26487	84433	+	JUNC_3174	1	GTAG	57946
63503	121402	+	JUNC_11077	1	GTAG	57899
47153	104979	+	JUNC_6786	1	GTAG	57826
3375	61107	-	JUNC_390	1	CTAC	57732
62704	120197	-	JUNC_10986	1	CTAC	57493
79381	136845	+	JUNC_13037	1	GTAG	57464
51674	108979	+	JUNC_7506	1	GTAG	57305
84413	141382	-	JUNC_13651	1	CTAC	56969

61425	118346	-	JUNC_10625	1	CTAC	56921
14836	71176	-	JUNC_1789	1	CTAC	56340
83742	139991	+	JUNC_13550	1	GTAG	56249
42113	98195	+	JUNC_5945	1	GTAG	56082
51878	107832	+	JUNC_7647	1	GTAG	55954
53675	109404	-	JUNC_8433	1	CTAC	55729
38398	93738	+	JUNC_5035	1	GTAG	55340
76707	132029	-	JUNC_12892	1	CTAC	55322
51095	106369	-	JUNC_7375	1	CTAC	55274
76707	131959	-	JUNC_12891	1	CTAC	55252
55175	110368	-	JUNC_9130	1	CTAC	55193
71531	126571	+	JUNC_12541	1	GTAG	55040
59688	114597	-	JUNC_10017	1	CTAC	54909
42319	97224	-	JUNC_5983	1	CTAC	54905
62011	116814	+	JUNC_10867	1	GTAG	54803
54911	109594	-	JUNC_9002	1	CTAC	54683
55008	109600	+	JUNC_9055	1	GTAG	54592
39136	93594	+	JUNC_5227	1	GTAG	54458
85892	140303	-	JUNC_13872	1	CTAC	54411
73681	127795	+	JUNC_12698	1	GTAG	54114
25172	79075	-	JUNC_2791	1	CTAC	53903
86230	139794	-	JUNC_13930	1	CTAC	53564
69972	123528	-	JUNC_12151	1	CTAC	53556
49562	103073	-	JUNC_7179	1	CTAC	53511
40198	93632	-	JUNC_5568	1	CTAC	53434
60202	113420	+	JUNC_10230	1	GTAG	53218
91573	144671	-	JUNC_15204	1	CTAC	53098
79034	132055	+	JUNC_13016	1	GTAG	53021
70203	123192	+	JUNC_12210	1	GTAG	52989
52375	105297	-	JUNC_7899	1	CTAC	52922
10246	63153	-	JUNC_1231	1	CTAC	52907
37084	89865	+	JUNC_4647	1	GTAG	52781
38128	90835	-	JUNC_4936	1	CTAC	52707
79305	132003	-	JUNC_13029	1	CTAC	52698

44296	96928	+	JUNC_6252	1	GTAG	52632
38753	91220	+	JUNC_5127	1	GTAG	52467
55788	108246	+	JUNC_9367	1	GTAG	52458
55492	107820	+	JUNC_9272	1	GTAG	52328
26583	78910	+	JUNC_3199	1	GTAG	52327
2291	54526	+	JUNC_222	1	GTAG	52235
61959	114182	-	JUNC_10851	1	CTAC	52223
23497	75568	-	JUNC_2489	1	CTAC	52071
81042	133045	+	JUNC_13174	1	GTAG	52003
41447	93405	+	JUNC_5815	1	GTAG	51958
45664	97612	+	JUNC_6468	1	GTAG	51948
37885	89765	+	JUNC_4877	1	GTAG	51880
38110	89915	-	JUNC_4922	1	CTAC	51805
46068	97845	+	JUNC_6546	1	GTAG	51777
59704	111367	+	JUNC_10022	1	GTAG	51663
54558	106198	+	JUNC_8827	1	GTAG	51640
88799	140013	+	JUNC_14516	1	GTAG	51214
64352	115496	-	JUNC_11232	1	CTAC	51144
39529	90278	+	JUNC_5367	1	GTAG	50749
55436	106010	+	JUNC_9252	1	GTAG	50574
89866	140285	-	JUNC_14791	1	CTAC	50419
39481	89865	+	JUNC_5355	1	GTAG	50384
96750	147018	-	JUNC_16396	1	CTAC	50268
94795	144963	-	JUNC_16109	1	CTAC	50168
88334	138468	-	JUNC_14396	1	CTAC	50134
44958	95090	+	JUNC_6357	1	GTAG	50132
4893	54912	-	JUNC_648	1	CTAC	50019
14793	64746	+	JUNC_1787	1	GTAG	49953
86998	136813	-	JUNC_14073	1	CTAC	49815
72891	122608	+	JUNC_12643	1	GTAG	49717
63044	112718	-	JUNC_11027	1	CTAC	49674
66801	116463	+	JUNC_11609	1	GTAG	49662
54941	104454	+	JUNC_9019	1	GTAG	49513
54546	103945	+	JUNC_8816	1	GTAG	49399

72165	121492	+	JUNC_12584	1	GTAG	49327
73272	122562	+	JUNC_12672	1	GTAG	49290
50309	99567	+	JUNC_7279	1	GTAG	49258
46607	95615	+	JUNC_6628	1	GTAG	49008
39340	88100	-	JUNC_5302	1	CTAC	48760
93191	141916	-	JUNC_15558	1	CTAC	48725
55436	104097	+	JUNC_9251	1	GTAG	48661
60168	108661	+	JUNC_10216	1	GTAG	48493
26596	75049	+	JUNC_3203	1	GTAG	48453
70799	119141	-	JUNC_12463	1	CTAC	48342
59946	108240	+	JUNC_10111	1	GTAG	48294
99248	147368	+	JUNC_17323	1	GTAG	48120
76707	124814	-	JUNC_12890	1	CTAC	48107
84289	132156	-	JUNC_13627	1	CTAC	47867
60903	108682	+	JUNC_10474	1	GTAG	47779
37171	84900	+	JUNC_4682	1	GTAG	47729
4815	52456	-	JUNC_614	1	CTAC	47641
54911	102503	-	JUNC_9001	1	CTAC	47592
58878	106355	+	JUNC_9720	1	GTAG	47477
54410	101861	+	JUNC_8733	1	GTAG	47451
8561	55789	+	JUNC_942	1	GTAG	47228
61683	108742	+	JUNC_10766	1	GTAG	47059
3826	50784	+	JUNC_500	1	GTAG	46958
38498	85403	+	JUNC_5062	1	GTAG	46905
90058	136845	+	JUNC_14847	1	GTAG	46787
58698	105279	-	JUNC_9657	1	CTAC	46581
67098	113664	-	JUNC_11728	1	CTAC	46566
5557	52110	+	JUNC_807	1	GTAG	46553
54707	101147	+	JUNC_8894	1	GTAG	46440
54707	101045	+	JUNC_8893	1	GTAG	46338
94453	140342	-	JUNC_16054	1	CTAC	45889
105421	151301	+	JUNC_19277	1	GTAG	45880
53624	99223	-	JUNC_8390	1	CTAC	45599
52292	97867	+	JUNC_7851	1	GTAG	45575

96819	142326	-	JUNC_16423	1	CTAC	45507
36440	81823	-	JUNC_4461	1	CTAC	45383
19724	64938	-	JUNC_2200	1	CTAC	45214
93128	138299	+	JUNC_15533	1	GTAG	45171
47428	92516	+	JUNC_6896	1	GTAG	45088
3831	48879	-	JUNC_501	1	CTAC	45048
94813	139830	-	JUNC_16112	1	CTAC	45017
53270	98276	+	JUNC_8205	1	GTAG	45006
52332	97246	+	JUNC_7869	1	GTAG	44914
52346	97150	+	JUNC_7884	1	GTAG	44804
54749	99453	+	JUNC_8926	1	GTAG	44704
88588	133292	+	JUNC_14462	1	GTAG	44704
97199	141876	+	JUNC_16538	1	GTAG	44677
69272	113926	+	JUNC_11971	1	GTAG	44654
19365	63783	-	JUNC_2185	1	CTAC	44418
76935	121333	+	JUNC_12899	1	GTAG	44398
49562	93944	-	JUNC_7178	1	CTAC	44382
47188	91497	-	JUNC_6798	1	CTAC	44309
99951	144222	+	JUNC_17539	1	GTAG	44271
76736	120743	-	JUNC_12893	1	CTAC	44007
89060	132770	-	JUNC_14577	1	CTAC	43710
63421	107130	-	JUNC_11067	1	CTAC	43709
42369	86024	+	JUNC_5992	1	GTAG	43655
60591	104223	+	JUNC_10366	1	GTAG	43632
70300	113745	+	JUNC_12241	1	GTAG	43445
46654	89801	+	JUNC_6643	1	GTAG	43147
27635	70738	+	JUNC_3379	1	GTAG	43103
36107	79196	+	JUNC_4342	1	GTAG	43089
53483	96561	-	JUNC_8304	1	CTAC	43078
65231	108283	-	JUNC_11340	1	CTAC	43052
55436	98410	+	JUNC_9250	1	GTAG	42974
53624	96531	-	JUNC_8389	1	CTAC	42907
61959	104835	-	JUNC_10850	1	CTAC	42876
4921	47774	+	JUNC_668	1	GTAG	42853

55220	97895	-	JUNC_9151	1	CTAC	42675
40574	83200	+	JUNC_5671	1	GTAG	42626
26500	69100	+	JUNC_3179	1	GTAG	42600
53870	96423	-	JUNC_8521	1	CTAC	42553
53894	96417	-	JUNC_8532	1	CTAC	42523
45629	88113	-	JUNC_6458	1	CTAC	42484
51349	93824	-	JUNC_7419	1	CTAC	42475
98224	140645	-	JUNC_16932	1	CTAC	42421
51638	93879	+	JUNC_7480	1	GTAG	42241
16971	59176	+	JUNC_1994	1	GTAG	42205
45746	87870	+	JUNC_6483	1	GTAG	42124
54726	96850	+	JUNC_8903	1	GTAG	42124
53730	95807	+	JUNC_8468	1	GTAG	42077
17893	59965	+	JUNC_2102	1	GTAG	42072
54431	96501	-	JUNC_8744	1	CTAC	42070
12552	54580	+	JUNC_1602	1	GTAG	42028
5753	47774	+	JUNC_829	1	GTAG	42021
5769	47774	+	JUNC_830	1	GTAG	42005
66256	108246	+	JUNC_11483	1	GTAG	41990
5785	47774	+	JUNC_831	1	GTAG	41989
5801	47774	+	JUNC_833	1	GTAG	41973
5817	47774	+	JUNC_835	1	GTAG	41957
5833	47774	+	JUNC_837	1	GTAG	41941
5849	47774	+	JUNC_841	1	GTAG	41925
65675	107307	-	JUNC_11399	1	CTAC	41632
99297	140821	-	JUNC_17344	1	CTAC	41524
47476	88928	-	JUNC_6911	1	CTAC	41452
91462	132824	-	JUNC_15176	1	CTAC	41362
28884	70177	+	JUNC_3478	1	GTAG	41293
61318	102593	+	JUNC_10588	1	GTAG	41275
10008	51279	-	JUNC_1173	1	CTAC	41271
6044	47276	+	JUNC_848	1	GTAG	41232
6044	47265	+	JUNC_847	1	GTAG	41221
103838	144929	+	JUNC_18508	1	GTAG	41091

99297	140285	-	JUNC_17343	1	CTAC	40988
102433	143309	-	JUNC_18143	1	CTAC	40876
83259	124028	+	JUNC_13465	1	GTAG	40769
82227	122866	+	JUNC_13332	1	GTAG	40639
64541	105076	-	JUNC_11258	1	CTAC	40535
107012	147477	+	JUNC_19909	1	GTAG	40465
60495	100810	+	JUNC_10338	1	GTAG	40315
60255	100528	-	JUNC_10254	1	CTAC	40273
61689	101912	+	JUNC_10771	1	GTAG	40223
68327	108454	+	JUNC_11897	1	GTAG	40127
14309	54244	+	JUNC_1753	1	GTAG	39935
104453	144222	+	JUNC_18860	1	GTAG	39769
54821	94416	-	JUNC_8957	1	CTAC	39595
53483	92988	-	JUNC_8303	1	CTAC	39505
83176	122602	+	JUNC_13451	1	GTAG	39426
35833	75190	-	JUNC_4267	1	CTAC	39357
104960	144299	+	JUNC_19108	1	GTAG	39339
53675	92998	-	JUNC_8432	1	CTAC	39323
54512	93738	+	JUNC_8798	1	GTAG	39226
8127	47326	+	JUNC_926	1	GTAG	39199
100077	139272	-	JUNC_17573	1	CTAC	39195
38345	77297	+	JUNC_5006	1	GTAG	38952
10282	49083	+	JUNC_1241	1	GTAG	38801
24260	63045	-	JUNC_2634	1	CTAC	38785
101608	140285	-	JUNC_17947	1	CTAC	38677
54431	92998	-	JUNC_8743	1	CTAC	38567
51431	89915	-	JUNC_7435	1	CTAC	38484
101155	139496	+	JUNC_17864	1	GTAG	38341
58575	96558	-	JUNC_9614	1	CTAC	37983
70498	108352	-	JUNC_12310	1	CTAC	37854
35594	73381	+	JUNC_4164	1	GTAG	37787
102502	140285	-	JUNC_18160	1	CTAC	37783
83169	120946	-	JUNC_13449	1	CTAC	37777
104453	142225	+	JUNC_18859	1	GTAG	37772

102112	139593	-	JUNC_18058	1	CTAC	37481
91472	128929	+	JUNC_15183	1	GTAG	37457
51653	88923	+	JUNC_7491	1	GTAG	37270
62258	99512	+	JUNC_10956	1	GTAG	37254
69272	106485	+	JUNC_11970	1	GTAG	37213
59568	96760	+	JUNC_9963	1	GTAG	37192
76707	113897	-	JUNC_12889	1	CTAC	37190
23378	60488	+	JUNC_2467	1	GTAG	37110
105647	142540	+	JUNC_19375	1	GTAG	36893
15333	52169	-	JUNC_1833	1	CTAC	36836
36027	72700	+	JUNC_4314	1	GTAG	36673
103720	140291	-	JUNC_18432	1	CTAC	36571
66481	102933	-	JUNC_11519	1	CTAC	36452
96750	133073	-	JUNC_16395	1	CTAC	36323
58695	94959	-	JUNC_9653	1	CTAC	36264
100527	136721	-	JUNC_17695	1	CTAC	36194
102271	138432	-	JUNC_18104	1	CTAC	36161
106378	142529	+	JUNC_19685	1	GTAG	36151
51725	87660	-	JUNC_7541	1	CTAC	35935
36107	71949	+	JUNC_4341	1	GTAG	35842
55954	91775	+	JUNC_9436	1	GTAG	35821
60699	96423	-	JUNC_10401	1	CTAC	35724
53709	89427	+	JUNC_8455	1	GTAG	35718
100428	136136	+	JUNC_17659	1	GTAG	35708
60834	96531	-	JUNC_10446	1	CTAC	35697
5837	41448	+	JUNC_839	1	GTAG	35611
60162	95729	-	JUNC_10210	1	CTAC	35567
16542	52089	-	JUNC_1952	1	CTAC	35547
61759	97246	+	JUNC_10796	1	GTAG	35487
111587	146930	-	JUNC_20690	1	CTAC	35343
54332	89427	+	JUNC_8698	1	GTAG	35095
104073	139035	-	JUNC_18666	1	CTAC	34962
106384	141319	+	JUNC_19692	1	GTAG	34935
67582	102503	-	JUNC_11828	1	CTAC	34921

49562	84471	-	JUNC_7177	1	CTAC	34909
60576	95474	+	JUNC_10361	1	GTAG	34898
58425	93261	+	JUNC_9571	1	GTAG	34836
104015	138591	+	JUNC_18627	1	GTAG	34576
54453	88944	-	JUNC_8759	1	CTAC	34491
54243	88617	+	JUNC_8652	1	GTAG	34374
105834	140051	+	JUNC_19450	1	GTAG	34217
53811	87918	+	JUNC_8500	1	GTAG	34107
84413	118459	-	JUNC_13649	1	CTAC	34046
13267	47235	-	JUNC_1661	1	CTAC	33968
60318	94286	-	JUNC_10278	1	CTAC	33968
4832	38789	-	JUNC_633	1	CTAC	33957
53042	86970	+	JUNC_8092	1	GTAG	33928
5603	39530	+	JUNC_819	1	GTAG	33927
36806	70681	+	JUNC_4550	1	GTAG	33875
59946	93777	+	JUNC_10109	1	GTAG	33831
5421	39209	+	JUNC_778	1	GTAG	33788
60259	93879	+	JUNC_10256	1	GTAG	33620
54512	88128	+	JUNC_8797	1	GTAG	33616
73014	106495	+	JUNC_12652	1	GTAG	33481
8272	41662	-	JUNC_934	1	CTAC	33390
52689	86079	-	JUNC_8065	1	CTAC	33390
73467	106739	+	JUNC_12683	1	GTAG	33272
37220	70464	+	JUNC_4705	1	GTAG	33244
27619	60844	+	JUNC_3372	1	GTAG	33225
106197	139122	+	JUNC_19591	1	GTAG	32925
99981	132707	+	JUNC_17548	1	GTAG	32726
64184	96820	-	JUNC_11199	1	CTAC	32636
29599	62155	-	JUNC_3521	1	CTAC	32556
88283	120711	+	JUNC_14381	1	GTAG	32428
61593	93879	+	JUNC_10711	1	GTAG	32286
36246	68387	-	JUNC_4394	1	CTAC	32141
58626	90762	-	JUNC_9633	1	CTAC	32136
107692	139794	-	JUNC_20163	1	CTAC	32102

8157	40181	-	JUNC_927	1	CTAC	32024
54714	86446	+	JUNC_8897	1	GTAG	31732
54726	86372	+	JUNC_8902	1	GTAG	31646
57591	89232	-	JUNC_9529	1	CTAC	31641
62058	93578	+	JUNC_10881	1	GTAG	31520
73272	104709	+	JUNC_12671	1	GTAG	31437
109088	140362	-	JUNC_20469	1	CTAC	31274
102502	133521	-	JUNC_18159	1	CTAC	31019
87456	118461	-	JUNC_14182	1	CTAC	31005
30245	61165	+	JUNC_3562	1	GTAG	30920
38590	69483	+	JUNC_5086	1	GTAG	30893
105963	136842	+	JUNC_19508	1	GTAG	30879
68063	98933	+	JUNC_11873	1	GTAG	30870
59736	90551	-	JUNC_10028	1	CTAC	30815
54512	85262	+	JUNC_8796	1	GTAG	30750
15951	46662	+	JUNC_1897	1	GTAG	30711
30989	61690	+	JUNC_3672	1	GTAG	30701
54636	85178	+	JUNC_8864	1	GTAG	30542
36470	66649	+	JUNC_4473	1	GTAG	30179
103004	133151	-	JUNC_18267	1	CTAC	30147
21883	52002	-	JUNC_2331	1	CTAC	30119
38860	68957	+	JUNC_5161	1	GTAG	30097
27472	57535	+	JUNC_3351	1	GTAG	30063
39635	69688	+	JUNC_5392	1	GTAG	30053
61689	91722	+	JUNC_10770	1	GTAG	30033
8552	38546	+	JUNC_941	1	GTAG	29994
32756	62642	+	JUNC_3813	1	GTAG	29886
61671	91497	-	JUNC_10756	1	CTAC	29826
100410	130211	+	JUNC_17655	1	GTAG	29801
38753	68363	+	JUNC_5126	1	GTAG	29610
63503	93111	+	JUNC_11074	1	GTAG	29608
60232	89706	+	JUNC_10246	1	GTAG	29474
30983	60445	+	JUNC_3669	1	GTAG	29462
110382	139833	+	JUNC_20567	1	GTAG	29451

1892	31189	+	JUNC_129	1	GTAG	29297
106151	135389	-	JUNC_19570	1	CTAC	29238
59946	89028	+	JUNC_10106	1	GTAG	29082
106073	135117	-	JUNC_19549	1	CTAC	29044
105834	134861	+	JUNC_19449	1	GTAG	29027
61689	90641	+	JUNC_10769	1	GTAG	28952
24792	53676	-	JUNC_2677	1	CTAC	28884
65183	93900	+	JUNC_11334	1	GTAG	28717
22750	51423	-	JUNC_2372	1	CTAC	28673
104574	133238	+	JUNC_18927	1	GTAG	28664
27175	55789	+	JUNC_3309	1	GTAG	28614
76113	104709	+	JUNC_12851	1	GTAG	28596
42109	70666	+	JUNC_5943	1	GTAG	28557
78993	107333	-	JUNC_13011	1	CTAC	28340
26487	54750	+	JUNC_3173	1	GTAG	28263
61256	89427	+	JUNC_10568	1	GTAG	28171
8127	36279	+	JUNC_925	1	GTAG	28152
92979	120928	-	JUNC_15453	1	CTAC	27949
77766	105693	+	JUNC_12936	1	GTAG	27927
7780	35689	+	JUNC_909	1	GTAG	27909
27635	55437	+	JUNC_3378	1	GTAG	27802
112579	140291	-	JUNC_20804	1	CTAC	27712
65996	93708	+	JUNC_11445	1	GTAG	27712
112579	140270	-	JUNC_20803	1	CTAC	27691
100527	128215	-	JUNC_17694	1	CTAC	27688
77223	104851	+	JUNC_12916	1	GTAG	27628
27472	54984	+	JUNC_3350	1	GTAG	27512
96310	123670	-	JUNC_16269	1	CTAC	27360
40639	67904	+	JUNC_5704	1	GTAG	27265
28566	55817	+	JUNC_3456	1	GTAG	27251
25239	52432	+	JUNC_2823	1	GTAG	27193
54659	81823	-	JUNC_8873	1	CTAC	27164
79034	106198	+	JUNC_13015	1	GTAG	27164
86820	113887	-	JUNC_14044	1	CTAC	27067

25269	52293	+	JUNC_2835	1	GTAG	27024
69620	96531	-	JUNC_12026	1	CTAC	26911
6722	33581	+	JUNC_875	1	GTAG	26859
87233	114025	+	JUNC_14126	1	GTAG	26792
20524	47276	+	JUNC_2254	1	GTAG	26752
83059	109762	-	JUNC_13427	1	CTAC	26703
53870	80443	-	JUNC_8520	1	CTAC	26573
15023	41448	+	JUNC_1806	1	GTAG	26425
20881	47096	+	JUNC_2280	1	GTAG	26215
9331	35511	+	JUNC_989	1	GTAG	26180
12402	38537	+	JUNC_1587	1	GTAG	26135
63917	89915	-	JUNC_11145	1	CTAC	25998
7618	33581	+	JUNC_893	1	GTAG	25963
55873	81823	-	JUNC_9404	1	CTAC	25950
87629	113541	-	JUNC_14224	1	CTAC	25912
621	26461	+	JUNC_33	1	GTAG	25840
36505	62321	+	JUNC_4489	1	GTAG	25816
16170	41967	-	JUNC_1919	1	CTAC	25797
38645	64299	+	JUNC_5098	1	GTAG	25654
26638	52169	-	JUNC_3210	1	CTAC	25531
107360	132890	-	JUNC_20067	1	CTAC	25530
61689	87202	+	JUNC_10768	1	GTAG	25513
97760	123148	+	JUNC_16734	1	GTAG	25388
111587	136697	-	JUNC_20689	1	CTAC	25110
48367	73249	+	JUNC_7048	1	GTAG	24882
16191	40591	-	JUNC_1922	1	CTAC	24400
60060	84433	+	JUNC_10158	1	GTAG	24373
106375	130655	+	JUNC_19680	1	GTAG	24280
82300	106385	+	JUNC_13341	1	GTAG	24085
75651	99664	+	JUNC_12825	1	GTAG	24013
45746	69688	+	JUNC_6482	1	GTAG	23942
12593	36471	+	JUNC_1606	1	GTAG	23878
66080	89915	-	JUNC_11462	1	CTAC	23835
16964	40744	-	JUNC_1988	1	CTAC	23780

40441	64203	+	JUNC_5629	1	GTAG	23762
104574	128302	+	JUNC_18926	1	GTAG	23728
50309	74008	+	JUNC_7278	1	GTAG	23699
83434	107085	+	JUNC_13501	1	GTAG	23651
36862	60496	+	JUNC_4569	1	GTAG	23634
69613	93129	+	JUNC_12022	1	GTAG	23516
98559	122055	+	JUNC_17038	1	GTAG	23496
11571	35009	-	JUNC_1495	1	CTAC	23438
84422	107820	+	JUNC_13657	1	GTAG	23398
103730	127114	-	JUNC_18438	1	CTAC	23384
38443	61771	+	JUNC_5049	1	GTAG	23328
62	23379	+	JUNC_2	1	GTAG	23317
15960	39273	-	JUNC_1898	1	CTAC	23313
66242	89496	-	JUNC_11479	1	CTAC	23254
117419	140495	+	JUNC_21418	1	GTAG	23076
24482	47477	-	JUNC_2657	1	CTAC	22995
87659	110460	-	JUNC_14237	1	CTAC	22801
61959	84733	-	JUNC_10847	1	CTAC	22774
71383	94152	+	JUNC_12530	1	GTAG	22769
118703	141462	+	JUNC_21460	1	GTAG	22759
31769	54513	+	JUNC_3762	1	GTAG	22744
111587	134317	-	JUNC_20688	1	CTAC	22730
60168	82888	+	JUNC_10215	1	GTAG	22720
117655	140285	-	JUNC_21426	1	CTAC	22630
110459	133073	-	JUNC_20575	1	CTAC	22614
30989	53535	+	JUNC_3671	1	GTAG	22546
122851	145356	+	JUNC_21908	1	GTAG	22505
106430	128929	+	JUNC_19723	1	GTAG	22499
33152	55625	+	JUNC_3833	1	GTAG	22473
106375	128840	+	JUNC_19679	1	GTAG	22465
74281	96525	-	JUNC_12753	1	CTAC	22244
59599	81823	-	JUNC_9977	1	CTAC	22224
36775	58991	+	JUNC_4542	1	GTAG	22216
39439	61549	+	JUNC_5336	1	GTAG	22110

89051	111160	+	JUNC_14574	1	GTAG	22109
114677	136721	-	JUNC_21135	1	CTAC	22044
96500	118461	-	JUNC_16325	1	CTAC	21961
9378	31311	+	JUNC_1003	1	GTAG	21933
42950	64803	+	JUNC_6070	1	GTAG	21853
53564	75295	+	JUNC_8359	1	GTAG	21731
36203	57890	+	JUNC_4385	1	GTAG	21687
66909	88575	-	JUNC_11653	1	CTAC	21666
60274	81847	+	JUNC_10258	1	GTAG	21573
53967	75424	-	JUNC_8559	1	CTAC	21457
83685	104851	+	JUNC_13544	1	GTAG	21166
102801	123950	-	JUNC_18230	1	CTAC	21149
71994	93129	+	JUNC_12571	1	GTAG	21135
66035	87036	-	JUNC_11453	1	CTAC	21001
84413	105297	-	JUNC_13648	1	CTAC	20884
119533	140285	-	JUNC_21478	1	CTAC	20752
39454	60115	-	JUNC_5341	1	CTAC	20661
111377	132035	-	JUNC_20662	1	CTAC	20658
26643	47237	+	JUNC_3212	1	GTAG	20594
105938	126433	-	JUNC_19498	1	CTAC	20495
102097	122530	+	JUNC_18052	1	GTAG	20433
109716	130012	-	JUNC_20520	1	CTAC	20296
49562	69829	-	JUNC_7176	1	CTAC	20267
116652	136845	+	JUNC_21368	1	GTAG	20193
44161	64203	+	JUNC_6227	1	GTAG	20042
102559	122530	+	JUNC_18175	1	GTAG	19971
105445	125206	-	JUNC_19289	1	CTAC	19761
60354	80026	+	JUNC_10295	1	GTAG	19672
101255	120912	+	JUNC_17889	1	GTAG	19657
98149	117735	-	JUNC_16907	1	CTAC	19586
81822	101327	-	JUNC_13272	1	CTAC	19505
121241	140645	-	JUNC_21646	1	CTAC	19404
34243	53598	+	JUNC_3895	1	GTAG	19355
33783	53112	-	JUNC_3876	1	CTAC	19329

51349	70417	-	JUNC_7418	1	CTAC	19068
69034	88100	-	JUNC_11951	1	CTAC	19066
117664	136686	-	JUNC_21428	1	CTAC	19022
78681	97619	-	JUNC_12998	1	CTAC	18938
72886	91702	+	JUNC_12641	1	GTAG	18816
121106	139833	+	JUNC_21618	1	GTAG	18727
20545	39174	+	JUNC_2260	1	GTAG	18629
35510	54105	+	JUNC_4126	1	GTAG	18595
52002	70499	-	JUNC_7716	1	CTAC	18497
20490	38939	+	JUNC_2248	1	GTAG	18449
35510	53953	+	JUNC_4125	1	GTAG	18443
37036	55437	+	JUNC_4630	1	GTAG	18401
97641	115944	+	JUNC_16698	1	GTAG	18303
122485	140771	-	JUNC_21828	1	CTAC	18286
54596	72751	+	JUNC_8853	1	GTAG	18155
95728	113776	-	JUNC_16178	1	CTAC	18048
87713	105612	+	JUNC_14250	1	GTAG	17899
116705	134523	+	JUNC_21373	1	GTAG	17818
83897	101682	+	JUNC_13570	1	GTAG	17785
94415	112128	-	JUNC_16047	1	CTAC	17713
81759	99453	+	JUNC_13264	1	GTAG	17694
1667	19287	-	JUNC_98	1	CTAC	17620
72387	89787	-	JUNC_12603	1	CTAC	17400
127842	145235	+	JUNC_22325	1	GTAG	17393
119719	136894	+	JUNC_21484	1	GTAG	17175
82305	99463	+	JUNC_13342	1	GTAG	17158
79034	96115	+	JUNC_13014	1	GTAG	17081
114416	131468	-	JUNC_21085	1	CTAC	17052
103841	120867	+	JUNC_18512	1	GTAG	17026
4824	21840	-	JUNC_621	1	CTAC	17016
53624	70499	-	JUNC_8388	1	CTAC	16875
101233	118101	+	JUNC_17884	1	GTAG	16868
134440	151285	+	JUNC_23340	1	GTAG	16845
37699	54543	+	JUNC_4824	1	GTAG	16844

133264	150063	-	JUNC_23016	1	CTAC	16799
21114	37868	+	JUNC_2295	1	GTAG	16754
117464	134121	+	JUNC_21421	1	GTAG	16657
53155	69811	+	JUNC_8136	1	GTAG	16656
106197	122789	+	JUNC_19589	1	GTAG	16592
90490	107007	-	JUNC_15000	1	CTAC	16517
1050	17554	-	JUNC_60	1	CTAC	16504
90834	107333	-	JUNC_15085	1	CTAC	16499
80835	97260	-	JUNC_13140	1	CTAC	16425
37084	53505	+	JUNC_4645	1	GTAG	16421
54113	70499	-	JUNC_8596	1	CTAC	16386
38156	54513	+	JUNC_4947	1	GTAG	16357
105774	122020	+	JUNC_19433	1	GTAG	16246
48905	65138	+	JUNC_7119	1	GTAG	16233
15005	31189	+	JUNC_1804	1	GTAG	16184
94134	110287	-	JUNC_15939	1	CTAC	16153
117972	134098	+	JUNC_21433	1	GTAG	16126
54431	70460	-	JUNC_8741	1	CTAC	16029
31188	47154	+	JUNC_3693	1	GTAG	15966
131035	146985	+	JUNC_22452	1	GTAG	15950
98987	114667	+	JUNC_17198	1	GTAG	15680
123186	138846	-	JUNC_22013	1	CTAC	15660
61609	77257	+	JUNC_10727	1	GTAG	15648
121088	136716	+	JUNC_21610	1	GTAG	15628
66648	82228	+	JUNC_11555	1	GTAG	15580
18044	33581	+	JUNC_2113	1	GTAG	15537
86221	101720	-	JUNC_13926	1	CTAC	15499
98017	113499	-	JUNC_16849	1	CTAC	15482
39757	55159	+	JUNC_5416	1	GTAG	15402
68308	83678	-	JUNC_11890	1	CTAC	15370
121565	136857	+	JUNC_21740	1	GTAG	15292
106375	121636	+	JUNC_19678	1	GTAG	15261
100683	115901	-	JUNC_17746	1	CTAC	15218
132447	147592	-	JUNC_22675	1	CTAC	15145

130265	145410	+	JUNC_22395	1	GTAG	15145
106375	121417	+	JUNC_19677	1	GTAG	15042
39724	54750	+	JUNC_5410	1	GTAG	15026
117461	132474	+	JUNC_21419	1	GTAG	15013
106384	121176	+	JUNC_19691	1	GTAG	14792
9329	23970	+	JUNC_988	1	GTAG	14641
3711	18348	-	JUNC_484	1	CTAC	14637
102200	116816	-	JUNC_18084	1	CTAC	14616
82282	96850	+	JUNC_13336	1	GTAG	14568
26382	40881	+	JUNC_3141	1	GTAG	14499
127794	142225	+	JUNC_22323	1	GTAG	14431
99808	114025	+	JUNC_17483	1	GTAG	14217
122482	136686	-	JUNC_21827	1	CTAC	14204
44628	58826	+	JUNC_6318	1	GTAG	14198
51799	65958	+	JUNC_7595	1	GTAG	14159
83035	97168	+	JUNC_13423	1	GTAG	14133
59985	74086	-	JUNC_10127	1	CTAC	14101
80739	94796	-	JUNC_13121	1	CTAC	14057
99471	113318	-	JUNC_17385	1	CTAC	13847
124010	137805	+	JUNC_22108	1	GTAG	13795
39821	53598	+	JUNC_5436	1	GTAG	13777
55788	69420	+	JUNC_9364	1	GTAG	13632
38590	52218	+	JUNC_5085	1	GTAG	13628
130927	144542	+	JUNC_22436	1	GTAG	13615
119244	132857	-	JUNC_21467	1	CTAC	13613
121502	134995	+	JUNC_21728	1	GTAG	13493
91453	104643	-	JUNC_15173	1	CTAC	13190
92997	106152	-	JUNC_15469	1	CTAC	13155
127218	140349	-	JUNC_22300	1	CTAC	13131
57414	70542	-	JUNC_9520	1	CTAC	13128
99297	112403	-	JUNC_17342	1	CTAC	13106
42700	55789	+	JUNC_6039	1	GTAG	13089
73527	86460	+	JUNC_12685	1	GTAG	12933
124132	137040	+	JUNC_22147	1	GTAG	12908

103944	116692	+	JUNC_18579	1	GTAG	12748
60415	73144	-	JUNC_10314	1	CTAC	12729
16580	28998	+	JUNC_1958	1	GTAG	12418
91543	103945	+	JUNC_15199	1	GTAG	12402
78069	90454	-	JUNC_12958	1	CTAC	12385
82071	94416	-	JUNC_13309	1	CTAC	12345
10339	22599	+	JUNC_1260	1	GTAG	12260
122214	134432	+	JUNC_21793	1	GTAG	12218
94602	106644	+	JUNC_16087	1	GTAG	12042
54714	66649	+	JUNC_8896	1	GTAG	11935
58626	70480	-	JUNC_9630	1	CTAC	11854
136341	148161	-	JUNC_23628	1	CTAC	11820
123147	134947	+	JUNC_21997	1	GTAG	11800
121486	133265	-	JUNC_21724	1	CTAC	11779
42356	54028	+	JUNC_5990	1	GTAG	11672
119719	131076	+	JUNC_21483	1	GTAG	11357
55788	67123	+	JUNC_9362	1	GTAG	11335
36059	47213	+	JUNC_4329	1	GTAG	11154
55014	66063	+	JUNC_9059	1	GTAG	11049
71648	82363	+	JUNC_12547	1	GTAG	10715
134577	145214	+	JUNC_23377	1	GTAG	10637
104501	115089	-	JUNC_18876	1	CTAC	10588
138335	148890	-	JUNC_24045	1	CTAC	10555
59946	70291	+	JUNC_10105	1	GTAG	10345
88271	98437	-	JUNC_14378	1	CTAC	10166
54707	64850	+	JUNC_8891	1	GTAG	10143
126572	136700	-	JUNC_22282	1	CTAC	10128
104135	114182	-	JUNC_18713	1	CTAC	10047
85792	95720	+	JUNC_13859	1	GTAG	9928
105656	115568	-	JUNC_19379	1	CTAC	9912
47549	57451	+	JUNC_6938	1	GTAG	9902
51758	61594	+	JUNC_7570	1	GTAG	9836
96605	106407	+	JUNC_16349	1	GTAG	9802
70799	80593	-	JUNC_12462	1	CTAC	9794

87536	97246	+	JUNC_14197	1	GTAG	9710
44628	54333	+	JUNC_6317	1	GTAG	9705
123270	132870	-	JUNC_22027	1	CTAC	9600
141688	151252	-	JUNC_25045	1	CTAC	9564
130380	139939	-	JUNC_22397	1	CTAC	9559
88076	97619	-	JUNC_14329	1	CTAC	9543
83359	92860	+	JUNC_13485	1	GTAG	9501
44145	53505	+	JUNC_6222	1	GTAG	9360
53835	63063	+	JUNC_8510	1	GTAG	9228
61318	70464	+	JUNC_10587	1	GTAG	9146
45786	54873	+	JUNC_6488	1	GTAG	9087
61114	70066	+	JUNC_10524	1	GTAG	8952
63067	71964	-	JUNC_11033	1	CTAC	8897
24808	33581	+	JUNC_2679	1	GTAG	8773
52714	61465	+	JUNC_8069	1	GTAG	8751
105130	113831	-	JUNC_19197	1	CTAC	8701
77019	85696	-	JUNC_12902	1	CTAC	8677
42545	51168	-	JUNC_6016	1	CTAC	8623
97695	106309	+	JUNC_16717	1	GTAG	8614
43097	51692	-	JUNC_6095	1	CTAC	8595
97695	106205	+	JUNC_16716	1	GTAG	8510
25076	33581	+	JUNC_2755	1	GTAG	8505
82647	91092	-	JUNC_13391	1	CTAC	8445
69700	78070	-	JUNC_12049	1	CTAC	8370
28396	36668	+	JUNC_3443	1	GTAG	8272
53675	61936	-	JUNC_8429	1	CTAC	8261
139121	147321	-	JUNC_24272	1	CTAC	8200
25422	33581	+	JUNC_2879	1	GTAG	8159
134444	142512	+	JUNC_23342	1	GTAG	8068
105518	113499	-	JUNC_19318	1	CTAC	7981
137670	145625	-	JUNC_23924	1	CTAC	7955
88762	96538	-	JUNC_14506	1	CTAC	7776
76639	84347	+	JUNC_12886	1	GTAG	7708
105848	113455	+	JUNC_19456	1	GTAG	7607

102226	109830	+	JUNC_18092	1	GTAG	7604
82740	90278	+	JUNC_13397	1	GTAG	7538
80961	88335	-	JUNC_13164	1	CTAC	7374
91472	98831	+	JUNC_15182	1	GTAG	7359
105774	113091	+	JUNC_19432	1	GTAG	7317
91715	98933	+	JUNC_15224	1	GTAG	7218
52380	59597	+	JUNC_7905	1	GTAG	7217
121362	128501	+	JUNC_21682	1	GTAG	7139
133162	140285	-	JUNC_22973	1	CTAC	7123
98559	105621	+	JUNC_17037	1	GTAG	7062
58626	65657	-	JUNC_9629	1	CTAC	7031
91042	97867	+	JUNC_15105	1	GTAG	6825
39187	46009	+	JUNC_5254	1	GTAG	6822
53730	60488	+	JUNC_8467	1	GTAG	6758
100245	106883	+	JUNC_17607	1	GTAG	6638
100312	106941	+	JUNC_17628	1	GTAG	6629
107129	113698	-	JUNC_19967	1	CTAC	6569
40643	47154	+	JUNC_5706	1	GTAG	6511
46152	52557	+	JUNC_6562	1	GTAG	6405
130872	137051	-	JUNC_22429	1	CTAC	6179
55052	61198	+	JUNC_9072	1	GTAG	6146
48412	54556	+	JUNC_7055	1	GTAG	6144
133162	139145	-	JUNC_22972	1	CTAC	5983
106274	112238	-	JUNC_19622	1	CTAC	5964
132889	138846	-	JUNC_22854	1	CTAC	5957
59101	65007	-	JUNC_9807	1	CTAC	5906
55788	61690	+	JUNC_9361	1	GTAG	5902
27706	33581	+	JUNC_3390	1	GTAG	5875
46712	52542	+	JUNC_6664	1	GTAG	5830
46654	52447	+	JUNC_6642	1	GTAG	5793
67699	73468	+	JUNC_11842	1	GTAG	5769
54441	60094	+	JUNC_8751	1	GTAG	5653
88268	93900	+	JUNC_14377	1	GTAG	5632
23378	28804	+	JUNC_2466	1	GTAG	5426

54243	59566	+	JUNC_8651	1	GTAG	5323
91372	96459	-	JUNC_15158	1	CTAC	5087
116905	121955	-	JUNC_21404	1	CTAC	5050
25788	30825	+	JUNC_3003	1	GTAG	5037
96819	101814	-	JUNC_16422	1	CTAC	4995
139859	144839	+	JUNC_24539	1	GTAG	4980
86322	91267	+	JUNC_13955	1	GTAG	4945
133464	138384	+	JUNC_23107	1	GTAG	4920
106151	111047	-	JUNC_19569	1	CTAC	4896
138929	143816	-	JUNC_24223	1	CTAC	4887
101312	106198	+	JUNC_17899	1	GTAG	4886
4930	9792	-	JUNC_675	1	CTAC	4862
98719	103501	-	JUNC_17069	1	CTAC	4782
28803	33581	+	JUNC_3474	1	GTAG	4778
45437	50201	-	JUNC_6425	1	CTAC	4764
127912	132590	+	JUNC_22326	1	GTAG	4678
92997	97619	-	JUNC_15468	1	CTAC	4622
96291	100909	+	JUNC_16260	1	GTAG	4618
88574	93188	-	JUNC_14457	1	CTAC	4614
97919	102509	-	JUNC_16802	1	CTAC	4590
103828	108352	-	JUNC_18498	1	CTAC	4524
26853	31311	+	JUNC_3258	1	GTAG	4458
144950	149385	+	JUNC_25976	1	GTAG	4435
134556	138978	-	JUNC_23372	1	CTAC	4422
23249	27620	+	JUNC_2442	1	GTAG	4371
47701	52071	+	JUNC_6973	1	GTAG	4370
95680	100041	-	JUNC_16175	1	CTAC	4361
42950	47213	+	JUNC_6069	1	GTAG	4263
130839	134970	+	JUNC_22425	1	GTAG	4131
39139	43263	-	JUNC_5228	1	CTAC	4124
140284	144345	-	JUNC_24721	1	CTAC	4061
55332	59350	+	JUNC_9207	1	GTAG	4018
100470	104454	+	JUNC_17679	1	GTAG	3984
3452	7391	-	JUNC_415	1	CTAC	3939

118850	122789	+	JUNC_21462	1	GTAG	3939
93737	97623	+	JUNC_15787	1	GTAG	3886
93230	97089	-	JUNC_15577	1	CTAC	3859
132232	136073	+	JUNC_22662	1	GTAG	3841
84649	88473	-	JUNC_13699	1	CTAC	3824
90474	94291	-	JUNC_14994	1	CTAC	3817
66740	70460	-	JUNC_11584	1	CTAC	3720
29990	33677	+	JUNC_3545	1	GTAG	3687
115684	119357	+	JUNC_21282	1	GTAG	3673
23018	26690	+	JUNC_2403	1	GTAG	3672
92979	96553	-	JUNC_15452	1	CTAC	3574
55788	59350	+	JUNC_9360	1	GTAG	3562
54911	58394	-	JUNC_8996	1	CTAC	3483
71963	75424	-	JUNC_12568	1	CTAC	3461
60828	64203	+	JUNC_10441	1	GTAG	3375
35504	38861	+	JUNC_4119	1	GTAG	3357
40315	43653	-	JUNC_5597	1	CTAC	3338
134208	137520	-	JUNC_23275	1	CTAC	3312
30287	33581	+	JUNC_3572	1	GTAG	3294
81897	85159	-	JUNC_13280	1	CTAC	3262
83890	87104	+	JUNC_13565	1	GTAG	3214
36160	39174	+	JUNC_4368	1	GTAG	3014
104414	107419	+	JUNC_18839	1	GTAG	3005
3452	6445	-	JUNC_414	1	CTAC	2993
119796	122789	+	JUNC_21485	1	GTAG	2993
136303	139254	+	JUNC_23621	1	GTAG	2951
43640	46578	+	JUNC_6154	1	GTAG	2938
35510	38399	+	JUNC_4123	1	GTAG	2889
87403	90278	+	JUNC_14165	1	GTAG	2875
91496	94332	-	JUNC_15187	1	CTAC	2836
91472	94303	+	JUNC_15180	1	GTAG	2831
71980	74806	+	JUNC_12570	1	GTAG	2826
106197	109000	+	JUNC_19588	1	GTAG	2803
134097	136860	+	JUNC_23253	1	GTAG	2763

51725	54454	-	JUNC_7540	1	CTAC	2729
101801	104394	+	JUNC_17997	1	GTAG	2593
31020	33581	+	JUNC_3679	1	GTAG	2561
102834	105371	-	JUNC_18239	1	CTAC	2537
103944	106395	+	JUNC_18578	1	GTAG	2451
104993	107433	+	JUNC_19124	1	GTAG	2440
78290	80652	-	JUNC_12972	1	CTAC	2362
59349	61690	+	JUNC_9881	1	GTAG	2341
144758	147087	+	JUNC_25876	1	GTAG	2329
106384	108704	+	JUNC_19690	1	GTAG	2320
34780	37085	+	JUNC_3916	1	GTAG	2305
104037	106309	+	JUNC_18642	1	GTAG	2272
79340	81607	+	JUNC_13033	1	GTAG	2267
29990	32242	+	JUNC_3543	1	GTAG	2252
143382	145625	-	JUNC_25484	1	CTAC	2243
91426	93608	-	JUNC_15166	1	CTAC	2182
3093	5269	-	JUNC_319	1	CTAC	2176
37036	39209	+	JUNC_4629	1	GTAG	2173
71358	73528	-	JUNC_12526	1	CTAC	2170
71131	73273	+	JUNC_12494	1	GTAG	2142
44628	46713	+	JUNC_6316	1	GTAG	2085
53714	55789	+	JUNC_8458	1	GTAG	2075
38860	40907	+	JUNC_5160	1	GTAG	2047
86554	88512	-	JUNC_14006	1	CTAC	1958
137670	139593	-	JUNC_23923	1	CTAC	1923
49847	51753	-	JUNC_7210	1	CTAC	1906
96927	98831	+	JUNC_16450	1	GTAG	1904
56994	58879	+	JUNC_9506	1	GTAG	1885
31769	33581	+	JUNC_3760	1	GTAG	1812
103120	104897	+	JUNC_18288	1	GTAG	1777
104504	106275	-	JUNC_18883	1	CTAC	1771
84573	86303	-	JUNC_13683	1	CTAC	1730
99012	100735	+	JUNC_17211	1	GTAG	1723
59349	61027	+	JUNC_9880	1	GTAG	1678

70293	71964	-	JUNC_12237	1	CTAC	1671
104453	106022	+	JUNC_18858	1	GTAG	1569
139106	140645	-	JUNC_24268	1	CTAC	1539
88472	89990	-	JUNC_14427	1	CTAC	1518
143858	145373	+	JUNC_25511	1	GTAG	1515
121119	122608	+	JUNC_21621	1	GTAG	1489
37044	38531	-	JUNC_4634	1	CTAC	1487
90999	92456	+	JUNC_15100	1	GTAG	1457
50246	51606	+	JUNC_7260	1	GTAG	1360
103964	105315	+	JUNC_18589	1	GTAG	1351
44220	45527	+	JUNC_6235	1	GTAG	1307
98679	99970	+	JUNC_17053	1	GTAG	1291
87659	88944	-	JUNC_14236	1	CTAC	1285
123186	124462	-	JUNC_22008	1	CTAC	1276
1779	3055	+	JUNC_119	1	GTAG	1276
104938	106198	+	JUNC_19094	1	GTAG	1260
47499	48753	+	JUNC_6923	1	GTAG	1254
112456	113709	+	JUNC_20784	1	GTAG	1253
53675	54912	-	JUNC_8428	1	CTAC	1237
92997	94211	-	JUNC_15466	1	CTAC	1214
68683	69868	+	JUNC_11930	1	GTAG	1185
46712	47840	+	JUNC_6660	1	GTAG	1128
35355	36471	+	JUNC_4058	1	GTAG	1116
134195	135293	+	JUNC_23273	1	GTAG	1098
27364	28456	+	JUNC_3329	1	GTAG	1092
29000	30072	-	JUNC_3487	1	CTAC	1072
27364	28420	+	JUNC_3328	1	GTAG	1056
139266	140285	-	JUNC_24321	1	CTAC	1019
53504	54513	+	JUNC_8320	1	GTAG	1009
101318	102318	+	JUNC_17901	1	GTAG	1000
127218	128212	-	JUNC_22299	1	CTAC	994
149645	150639	+	JUNC_26488	1	GTAG	994
122977	123950	-	JUNC_21939	1	CTAC	973
2291	3264	+	JUNC_217	1	GTAG	973

53111	54081	-	JUNC_8117	1	CTAC	970
84721	85670	-	JUNC_13712	1	CTAC	949
142170	143110	+	JUNC_25150	1	GTAG	940
90834	91739	-	JUNC_15083	1	CTAC	905
137670	138574	-	JUNC_23919	1	CTAC	904
144473	145373	+	JUNC_25726	1	GTAG	900
54203	55086	+	JUNC_8635	1	GTAG	883
105457	106309	+	JUNC_19296	1	GTAG	852
79195	80023	+	JUNC_13024	1	GTAG	828
56229	57049	+	JUNC_9468	1	GTAG	820
36712	37529	+	JUNC_4529	1	GTAG	817
47212	48016	+	JUNC_6809	1	GTAG	804
27619	28417	+	JUNC_3370	1	GTAG	798
91573	92354	-	JUNC_15203	1	CTAC	781
14823	15599	-	JUNC_1788	1	CTAC	776
46682	47426	+	JUNC_6652	1	GTAG	744
134231	134965	+	JUNC_23279	1	GTAG	734
132451	133181	-	JUNC_22676	1	CTAC	730
106333	107060	+	JUNC_19650	1	GTAG	727
91774	92456	+	JUNC_15233	1	GTAG	682
134646	135293	+	JUNC_23400	1	GTAG	647
66600	67230	+	JUNC_11549	1	GTAG	630
80961	81571	-	JUNC_13161	1	CTAC	610
104504	105102	-	JUNC_18882	1	CTAC	598
59349	59947	+	JUNC_9879	1	GTAG	598
88445	89031	-	JUNC_14420	1	CTAC	586
53669	54244	+	JUNC_8424	1	GTAG	575
1630	2195	+	JUNC_89	1	GTAG	565
48191	48753	+	JUNC_7036	1	GTAG	562
139793	140349	-	JUNC_24501	1	CTAC	556
51902	52456	-	JUNC_7655	1	CTAC	554
107342	107886	-	JUNC_20058	1	CTAC	544
83434	83969	+	JUNC_13500	1	GTAG	535
61314	61834	+	JUNC_10585	1	GTAG	520

41652	42147	+	JUNC_5847	1	GTAG	495
47482	47973	+	JUNC_6916	1	GTAG	491
104246	104709	+	JUNC_18767	1	GTAG	463
4233	4676	-	JUNC_554	1	CTAC	443
121565	122008	+	JUNC_21739	1	GTAG	443
139846	140285	-	JUNC_24535	1	CTAC	439
40174	40612	+	JUNC_5560	1	GTAG	438
54453	54889	-	JUNC_8758	1	CTAC	436
123632	124065	+	JUNC_22066	1	GTAG	433
61314	61744	+	JUNC_10584	1	GTAG	430
61412	61834	+	JUNC_10618	1	GTAG	422
122607	123025	+	JUNC_21851	1	GTAG	418
134790	135198	+	JUNC_23434	1	GTAG	408
60735	61122	-	JUNC_10412	1	CTAC	387
95737	96115	+	JUNC_16180	1	GTAG	378
99162	99519	-	JUNC_17285	1	CTAC	357
51947	52293	+	JUNC_7690	1	GTAG	346
87773	88113	-	JUNC_14266	1	CTAC	340
144495	144828	+	JUNC_25739	1	GTAG	333
69536	69868	+	JUNC_12000	1	GTAG	332
42950	43278	+	JUNC_6068	1	GTAG	328
74379	74707	+	JUNC_12770	1	GTAG	328
24940	25264	+	JUNC_2711	1	GTAG	324
88334	88652	-	JUNC_14395	1	CTAC	318
100440	100753	+	JUNC_17663	1	GTAG	313
91438	91739	-	JUNC_15170	1	CTAC	301
113999	114299	-	JUNC_21003	1	CTAC	300
89030	89328	-	JUNC_14569	1	CTAC	298
37115	37413	+	JUNC_4661	1	GTAG	298
60834	61122	-	JUNC_10445	1	CTAC	288
90163	90446	-	JUNC_14889	1	CTAC	283
36806	37085	+	JUNC_4549	1	GTAG	279
96261	96538	-	JUNC_16249	1	CTAC	277
96270	96545	-	JUNC_16253	1	CTAC	275

36964	37238	-	JUNC_4602	1	CTAC	274
120785	121059	-	JUNC_21533	1	CTAC	274
5182	5456	+	JUNC_725	1	GTAG	274
94266	94536	-	JUNC_15995	1	CTAC	270
106384	106644	+	JUNC_19689	1	GTAG	260
105927	106182	+	JUNC_19495	1	GTAG	255
61593	61834	+	JUNC_10709	1	GTAG	241
70311	70551	-	JUNC_12244	1	CTAC	240
145385	145625	-	JUNC_26151	1	CTAC	240
81585	81823	-	JUNC_13242	1	CTAC	238
54749	54984	+	JUNC_8921	1	GTAG	235
141507	141737	-	JUNC_25029	1	CTAC	230
36964	37178	-	JUNC_4600	1	CTAC	214
107119	107333	-	JUNC_19962	1	CTAC	214
81759	81973	+	JUNC_13263	1	GTAG	214
42739	42951	+	JUNC_6047	1	GTAG	212
144236	144445	+	JUNC_25614	1	GTAG	209
142920	143126	-	JUNC_25342	1	CTAC	206
59889	60094	+	JUNC_10082	1	GTAG	205
70498	70699	-	JUNC_12307	1	CTAC	201
66481	66680	-	JUNC_11518	1	CTAC	199
133072	133265	-	JUNC_22936	1	CTAC	193
38398	38591	+	JUNC_5034	1	GTAG	193
5254	5446	-	JUNC_742	1	CTAC	192
27221	27413	+	JUNC_3312	1	GTAG	192
120795	120987	+	JUNC_21535	1	GTAG	192
18074	18264	-	JUNC_2115	1	CTAC	190
97442	97627	-	JUNC_16607	1	CTAC	185
52033	52218	+	JUNC_7730	1	GTAG	185
55037	55221	-	JUNC_9067	1	CTAC	184
74058	74239	+	JUNC_12725	1	GTAG	181
141951	142129	-	JUNC_25097	1	CTAC	178
10123	10301	+	JUNC_1197	1	GTAG	178
106151	106326	-	JUNC_19568	1	CTAC	175

39862	40034	+	JUNC_5449	1	GTAG	172
37084	37253	+	JUNC_4644	1	GTAG	169
136673	136842	+	JUNC_23676	1	GTAG	169
107792	107958	-	JUNC_20199	1	CTAC	166
88574	88737	-	JUNC_14456	1	CTAC	163
80961	81121	-	JUNC_13160	1	CTAC	160
42388	42546	-	JUNC_5993	1	CTAC	158
134404	134557	-	JUNC_23326	1	CTAC	153
145037	145188	+	JUNC_26006	1	GTAG	151
6740	6889	-	JUNC_876	1	CTAC	149
119352	119501	+	JUNC_21469	1	GTAG	149
91166	91311	-	JUNC_15120	1	CTAC	145
96314	96459	-	JUNC_16272	1	CTAC	145
93593	93738	+	JUNC_15723	1	GTAG	145
35817	35959	+	JUNC_4263	1	GTAG	142
113559	113698	-	JUNC_20910	1	CTAC	139
61593	61724	+	JUNC_10708	1	GTAG	131
51349	51476	-	JUNC_7417	1	CTAC	127
120071	120197	-	JUNC_21489	1	CTAC	126
6044	6170	+	JUNC_846	1	GTAG	126
25737	25855	-	JUNC_2982	1	CTAC	118
96156	96270	+	JUNC_16207	1	GTAG	114
84787	84900	+	JUNC_13726	1	GTAG	113
69613	69723	+	JUNC_12020	1	GTAG	110
63917	64026	-	JUNC_11144	1	CTAC	109
106378	106485	+	JUNC_19684	1	GTAG	107
104358	104464	-	JUNC_18820	1	CTAC	106
133251	133355	-	JUNC_23011	1	CTAC	104
93523	93626	-	JUNC_15688	1	CTAC	103
54936	55038	+	JUNC_9017	1	GTAG	102
58749	58850	-	JUNC_9676	1	CTAC	101
74218	74317	+	JUNC_12742	1	GTAG	99
62025	62122	+	JUNC_10873	1	GTAG	97
95473	95568	+	JUNC_16156	1	GTAG	95

54821	54912	-	JUNC_8955	1	CTAC	91
39187	39278	+	JUNC_5253	1	GTAG	91
123186	123276	-	JUNC_22005	1	CTAC	90
2965	3055	+	JUNC_295	1	GTAG	90
62025	62114	+	JUNC_10872	1	GTAG	89
37084	37172	+	JUNC_4643	1	GTAG	88
107262	107348	-	JUNC_20024	1	CTAC	86
60048	60130	-	JUNC_10149	1	CTAC	82
54671	54750	+	JUNC_8877	1	GTAG	79
40315	40391	-	JUNC_5596	1	CTAC	76
52287	52363	-	JUNC_7847	1	CTAC	76
41652	41728	+	JUNC_5846	1	GTAG	76
91237	91311	-	JUNC_15129	1	CTAC	74
134486	134557	-	JUNC_23350	1	CTAC	71
38398	38468	+	JUNC_5033	1	GTAG	70
45595	45665	+	JUNC_6454	1	GTAG	70
3307	3376	-	JUNC_381	1	CTAC	69
104028	104097	+	JUNC_18633	1	GTAG	69
122865	122934	+	JUNC_21910	1	GTAG	69
97223	97291	-	JUNC_16544	1	CTAC	68
151438	151505	+	JUNC_26536	1	GTAG	67
97382	97448	-	JUNC_16587	1	CTAC	66
88099	88163	-	JUNC_14338	1	CTAC	64
68386	68447	-	JUNC_11899	1	CTAC	61
89426	89487	+	JUNC_14661	1	GTAG	61
106151	106211	-	JUNC_19567	1	CTAC	60
122461	122520	-	JUNC_21825	1	CTAC	59
3721	3780	+	JUNC_489	1	GTAG	59
96762	96820	-	JUNC_16402	1	CTAC	58
10282	10340	+	JUNC_1240	1	GTAG	58
119819	119875	-	JUNC_21486	1	CTAC	56
6366	6422	+	JUNC_859	1	GTAG	56
56994	57049	+	JUNC_9503	1	GTAG	55
36107	36161	+	JUNC_4340	1	GTAG	54

132935	132988	-	JUNC_22877	1	CTAC	53
3093	3126	-	JUNC_315	1	CTAC	33
123115	123148	+	JUNC_21984	1	GTAG	33

ıctions description="Mapsplice junctions"
