# Supplementary material for: Hidden regulation of herpes simplex virus 1 pre-mRNA splicing and polyadenylation by virally encoded immediate early gene ICP27
Source: PLoS Pathog. 2019 Jun 17;15(6):e1007884. doi: 10.1371/journal.ppat.1007884 (PMC6597130; doi:10.1371/journal.ppat.1007884)
Supplement: S3 Table — (PDF) [file ppat.1007884.s003.pdf]

Table S3. Sequences of synthesized oligonucleotide primers, probes and point mutations for splicing reporter genes.

Name of Genes	Name of Primers	Sequence of the Primers
LAT Exon 1 forward (Primer set 1)	oST1174	CCCGGTGTCGTTCAACAAAGAC
LAT Exon 2 backward forward (for Primer set 1 and 2)	oST1173	CCTCCTCCGCTTCCGCCTCCT
LAT Intron 1 forward (primer set 2 for 1.68kb and 1.45 kb intron)	oST1172	TTACTTACCCGTCCGACCACCAACT
ICP0 Exon 2 forward	oST1077	TTATCTGGACGGGCAATCAGCGGT
ICP0 Exon 3 reverse	oST1078	GTGGTGTTAGTGTTACTGCTGCCGTGT
ICP0 Exon 1 forward	oST1198	GTCAGCGACCCTCCAGCCGCATA
ICP0 intron 1 forward	oST1199	CGTGGTCCCCACTGACTCATACGCA
ICP0 Exon2 reverse	oST1197	CTCGAACAGTTCCGTGTCCGTGCTGT
UL15 forward	oST1119	CGAGCAGGGCCAGGGCGAAGT
UL15 forward	oST1110	CAGGTCGCTGTCTGCGGGACTCA
UL15 forward	oST1111	TGGGCGGCATCTCGGTCCATTC
UL15 forward	oST1112	TGTTTGAGGAGATCGACGCCTGCCT
UL15 forward	oST1118	CATCAGGGCGGCCAGAGGGAT
UL15 reverse	oST1113	GGCTGGAGGCAAACACGATGGT
UL15 reverse	oST1114	CCCATAATCGTCTGGACCGCAT
ICP34.5 Exon 1 forward (for both Primer set 1 and 4)	oST1069	CGCACATGCTTGCCTGTCAAACTCTAC
ICP34.5 reverse (for Primer set 1 and 2)	oST1071	TCAGGCCGGCGGGTACTCGCT
ICP34.5 reverse (for Primer set 4)	oST1074	CTGTCCGGCCAGTCGTCGTCGT
ICP34.5 Intron forward (for Primer set 2 and 3)	oST1169	CGCTCAATGAACCCGCATTGGT
ICP0 Exon 2 reverse (for Primer set 3)	oST1073	CGAACAGTTCCGTGTCCGTGCTGT
ICP34.5 forward (For Northern blot probe plasmid cloning)	oST11075B	GTTACCTGGGACTGTGCGGTTGGGA
ICP34.5 reverse (For Northern blot probe plasmid cloning)	oST1076	GGCCGAGACTAGCGAGTTAGACAGGCA
UL52 forward	oST1126	GCGCGGATCATCTCATATTGTTCCT
UL52 reverse	oST1127	GCGTCGTCGTAGCAGGGAAACCAGT
UL5 forward (spanning intron)	oST1025	GCAACCGCACGCTGCGCGAGTA
UL5 reverse (spanning intron)	oST1026	CGAGGGTCCTCTGGGTCGCATCCA
UL5 exon 2 forward	oST1027	GGCTGCGAGATGCTGACCGGCTC
UL5 exon 2 reverse	oST1028	GAGGCCCTGGCGTGGCATAGGCTA
UL4 forward	oST1029	CACCGCGAGATGTCCAATCCACAG
UL4 reverse	oST1030	GCGAGGAGCTGACCCAGGAGTCGA
Pri-miR-H6/UDG reverse	oST1035A	ACGGGTTTCCGTCGAGGCATCGT
Pri-miR-H6/UDG forward<br>*Pri-miR-H6/UDG* forward	oST1034A<br>oST1153	ATGCCTTCTTGGAGTACGTGGGTCA<br>TATAAAGGCTCAGATGACGCAGCAA
gL forward	oST1089	TCGTGGTTGTGTTACTGGGCAAACA
gL reverse	oST1087	GTCACACCAGCGGGACAGCCTCTA
UL37 forward	oST1105	GGCTAACGGACGAAACGCGGA
UL37 reverse	oST1106	CACCACGCCTTCCAGGATGACA
US3 forward	oST1083	GGTGCTCGTTGGTTGGCACTCA
US3 backward	oST1085	CCTGTCCATGGCGTCCAGCTCTTCT
gH forward	oST1079	CGCGGATCAGGGCGTTGTAGTGT
gH reverse	oST1081	CCGACGACGGAGCTGGACATCA
UL12 forward	oST1064	CGGAGAACGAGAGGCCCATAGCT
UL12 reverse	oST1065	GAGGAGGCTCTCGTCACGCAA
gE forward	oST1108	GCGTGTATGACCTGTTGGCGCA
gE reverse	oST1109	GGGTTTCTTCTCGGTGTTTGTGT
Ul24 forward	oST1124	GGAGCCAGAACGGCGTCGGTCA
Ul24 reverse	oST1125	GTCCGTTGGGCGACAAACACCA
UL34 forward	oST1159	TTCCTGGACCTGCACCAGATTCT
UL34 reverse	oST1160	GCTGATCCACATGCTGGCCATCAT
VHS forward	oST1066	AGCAGTAACCAGGTCCGTCCAGA
VHS reverse	oST1067	GGTCGTCCTCCTCGGAGATACTCAC
gD forward	oST1103	AGCTTCAGCGCCGTCAGCGA
gD reverse	oST1104	GAGTTCTGGCTGCGTGGCGT
gB forward	oST1115	TTTCACCGGGACGACCACGAGA
gB reverse	oST1116	CAACCTGACCGAGTACCCGCTCT
ICP8 forward	oST1120	CTTCAAGGAGGCCGTGTGCATCA
ICP8 reverse	oST1121	GGTTGGAGTCCGCGACGTATCGA
US11 forward	oST1128	GGCGGGACCGTGACCGACAGT
US11 reverse	oST1129	CGTCCAGAGGGTAGAGCCCTGAGT
TK forward	oST1122	ACCACCACCACGCAACTGCTGGT
TK reverse	oST1123	CGCGGGTTCCTTCCGGTATTGTCT
UL42 forward<br>*UL42 reverse*	oST1161<br>oST1162	GTGAGCGACGGTCCGGTTGCT<br>GAGTGACCCGCGTGGTCGACGA
UL46 forward	oSt1163	GGCCGTCAGATACGTGGCTTCCA

UL46 reverse	oST1164	ACGTCCTGGGCCACCTGACGT
UL26 forward	oST1195	CTGTTCTCGGGACCCAGCCCACT
UL26 reverse	oST1196	CGTGTGCTGCGCTGCTGGCGT
UL22C forward	oST1177	CATCGCACCCGAGCACCAGCT
UL22C reverse	oST1178	GCTCCATACCGACGATCTGCGA
UL4-5C forward	oST1176	GCGTTCGAGCTTCTGGTTGCTGT
UL4-5C reverse	oST1175	GGACGAACAACAGCTCGACGTGT
ICP47 forward	oST1130	GACAGAAACCCACCGGTCCGCCT
ICP47 reverse	oST1131	CGCATGTTGTCCAGGAAGGTGTC
UL49SC forward	oST1191	GGGAGTGCGGCGGATTCTGGCT
UL49SC backward	oST1192	GGCTCGTCATCCGAAGACGACGA
UL41-42C Exon 1 forward	oST1201	AAAGCAGTAACCAGGTCCGTCCA
UL41-42C Exon 3 reverse	oST1202	CAAGCAACCGGACCGTCGCT
UL41-42C Exon 2 reverse	oST1204	GTCCAGGTCCCGAACGCGCA
UL41-42C Exon 2 forward	oST1205	GCGTCGTGGATGACGTGGCGT
LAT Northern blot Probe1 (gBlock)		GTAGGTTAGACACCTGCTTCTCCCCAATAGAGGGGGGGGGACCCAAACGACAGGGGGCG CCCCAGAGGCTAAGGTCGGCCACGCCACTCGCGGGTGGGCTCGTGTTACAGCACACCAG CCCGTTATTTTCCCCCCCTCCCACCCTTAGTTAGACTCTGTTACTTACCCGTCCGACCACC AACTGCCCCCTTATCTAAGGGCCGGCTGGAAGACCGCCAGGGGGTCGGCCGGTGTCGCT GTAACCCCCCACGCCAATGACCCACGTACTCCAAGAAG
LAT Northern blot Probe 2 (gBlock)		TTGTGGCGGCCAGAGGCCAGGTCAGTCCGGGCGGGCAGGCGCTCGCGGAAACTTAACAC CCACACCCAACCCACTGTGGTTCTGGCTCCATGCCAGTGGCAGGATGCTTTCGGGGATCG GTGGTCAGGCAGCCCGGGCCGCGGCTCTGTGGTTAACACCAGAGCCTGCCCAACATGGC ACCCCCACTCCCACGCACC
