# Supplementary figures and images for: Hidden regulation of herpes simplex virus 1 pre-mRNA splicing and polyadenylation by virally encoded immediate early gene ICP27
Source: PLoS Pathog. 2019 Jun 17;15(6):e1007884. doi: 10.1371/journal.ppat.1007884 (PMC6597130; doi:10.1371/journal.ppat.1007884)

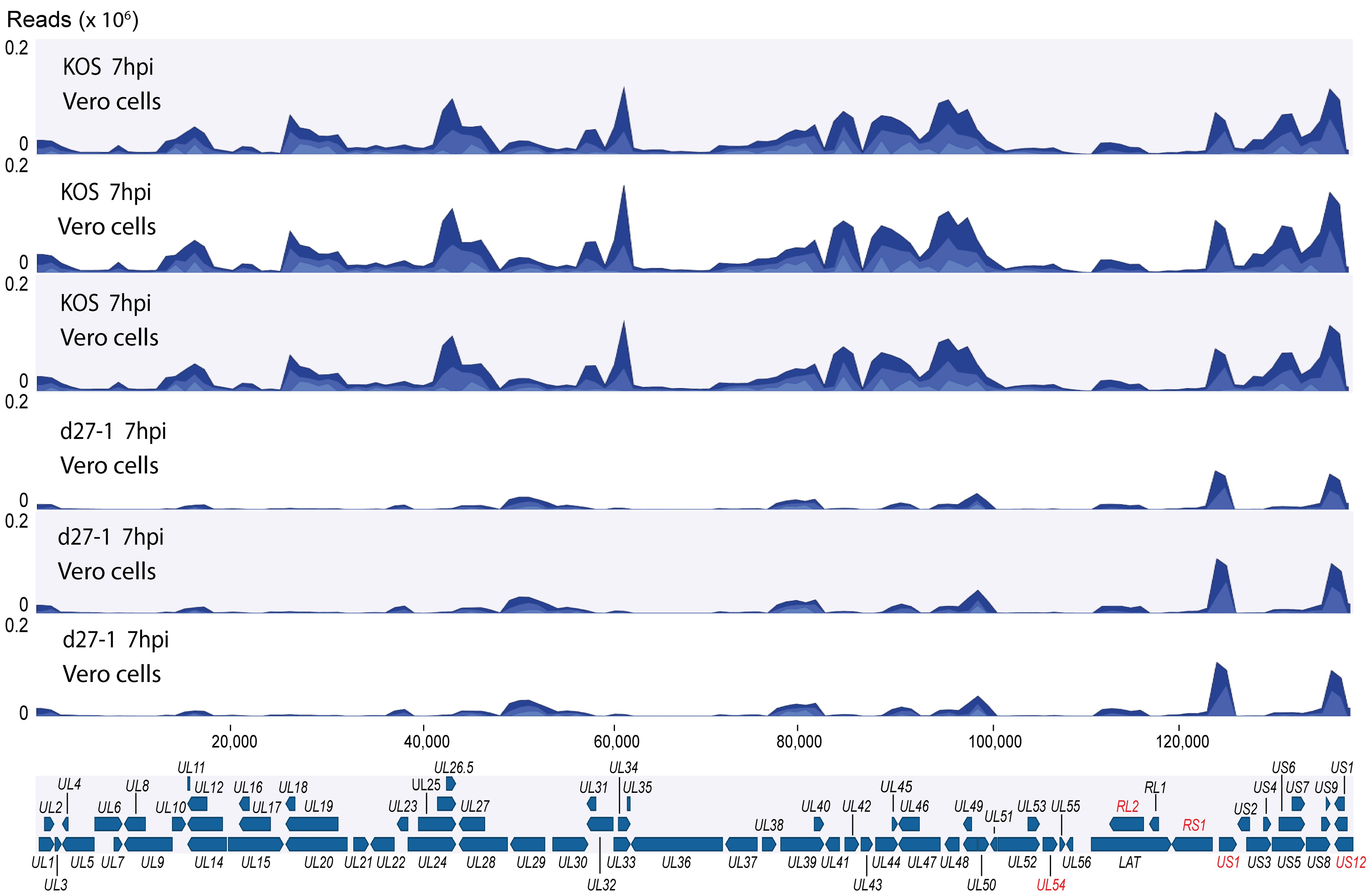

Supplement: S1 Fig — RNA sequences from Vero cells infected with an HSV-1 ICP27 deletion mutant (d27-1) or its wild-type parental strain (KOS) at 7 hpi (in triplicate) were aligned to the HSV-1 genome (after removal of terminal repeat sequences, which are represented by internal repeats) and graphed as number of viral reads at each genome location. Genome positions of HSV genes relative to the trimmed genome are shown under the graph. Expression of HSV-1 IE genes including RL2 (ICP0), RS1 (ICP4), US1 (ICP22) and US12 (ICP47) labelled in red was similar between KOS or d27-1 infected cells. IE gene UL54 (ICP27) is not detectable in d27-1 infected cells since the coding region of UL54 was deleted in d27-1. (TIF) [file ppat.1007884.s004.tif]

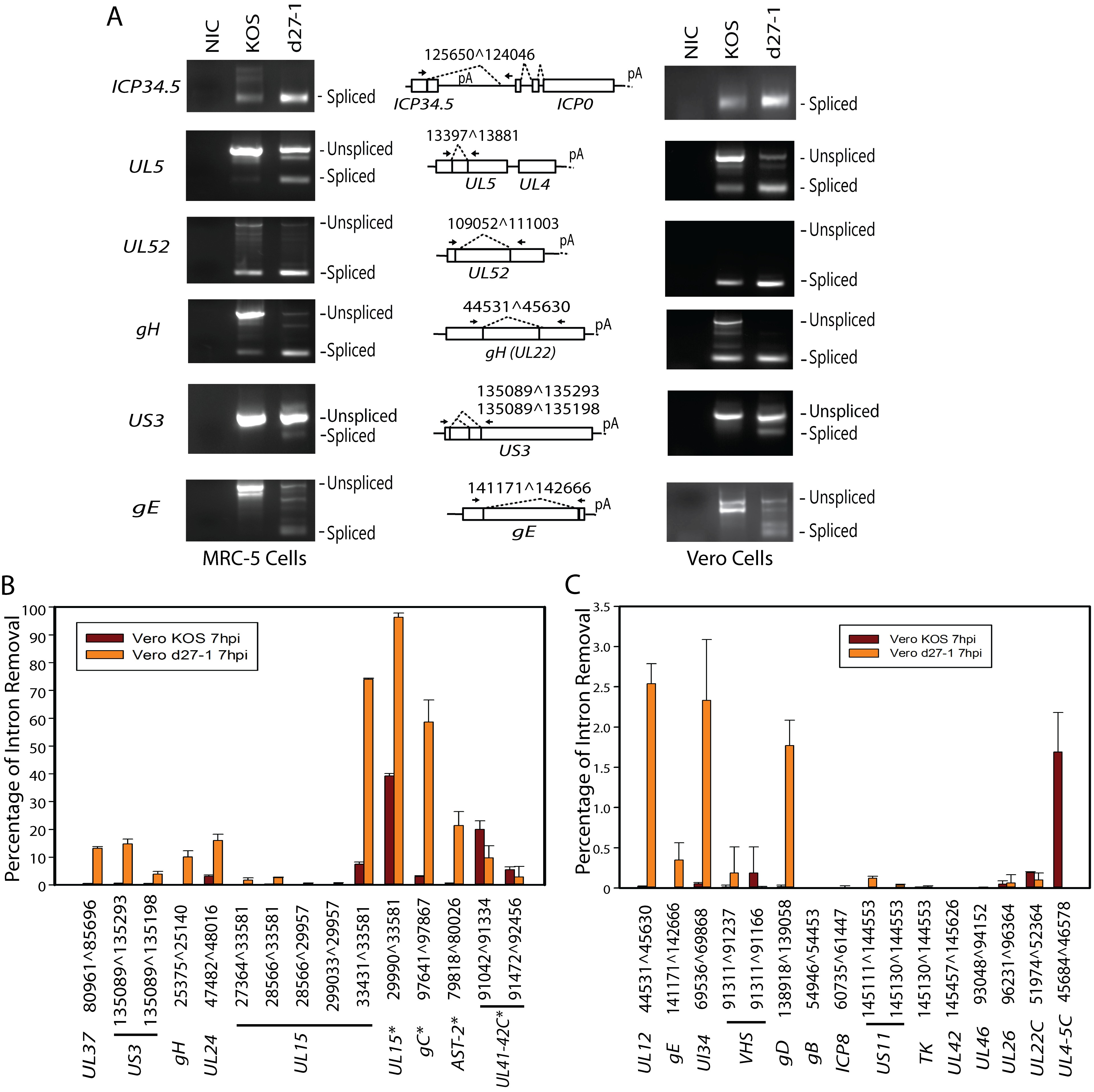

Supplement: S2 Fig — (A) cDNAs were prepared from total RNAs from KOS or d27-1 infected MRC-5 cells at 7hpi (left) or Vero cells at 8 hpi (right). Novel spliced isoforms were amplified using primers specific to the representative genes listed. The diagrams in the middle illustrate the relative location of the primers and the verified splice sites. Splicing efficiency of spliced variants listed in Table 2 but not described in other figures were determined by mapping the high throughput sequencing data obtained from infected Vero cells (in triplicate) (B) for transcripts with relative splicing efficiency ≥5% or (C) for transcripts with relative splicing efficiency <5%. *Relative splicing efficiency of previously identified non-IE spliced genes including gC, AST-2 and UL41-42C were also included in the analysis. (TIF) [file ppat.1007884.s005.tif]

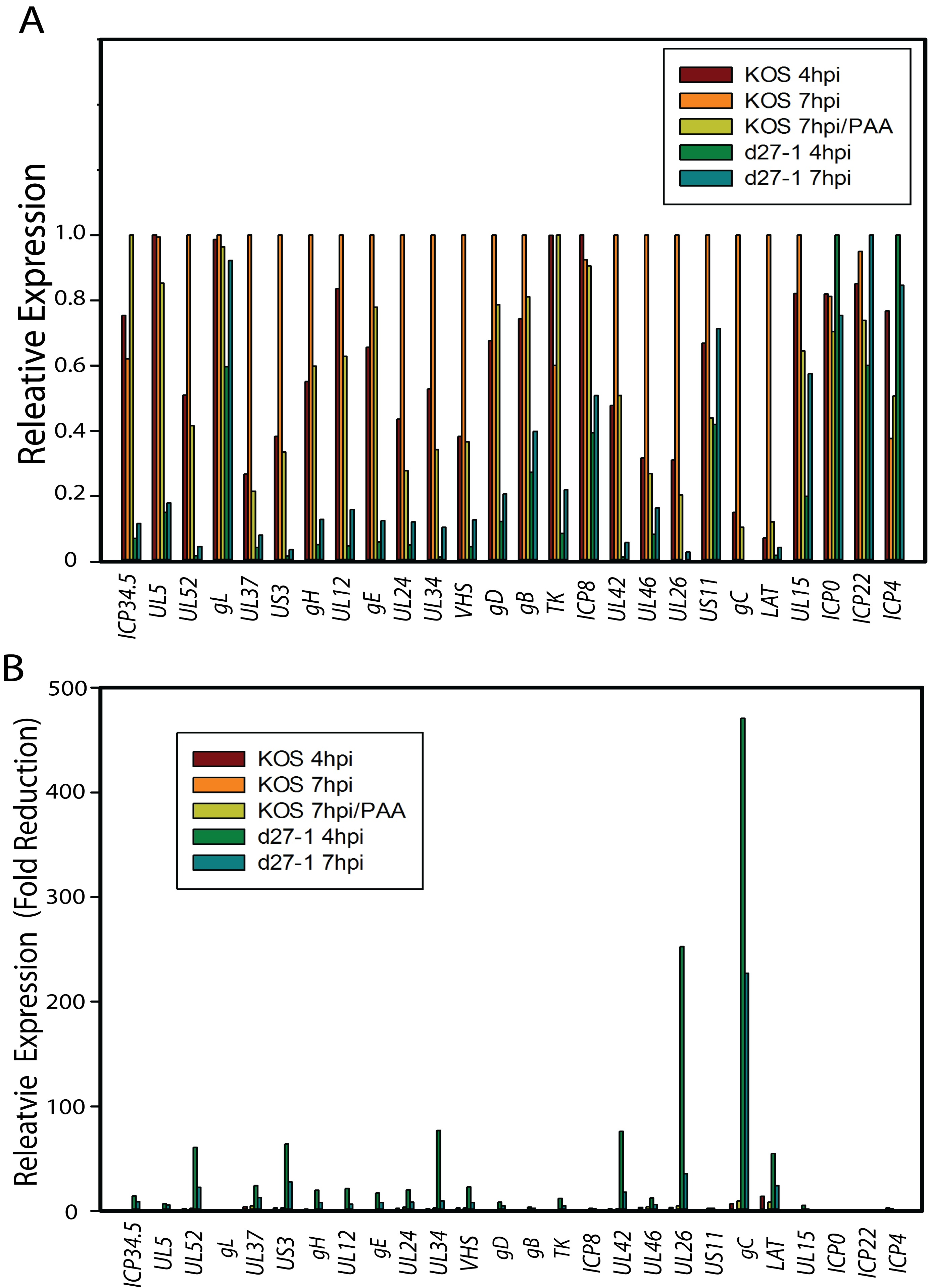

Supplement: S3 Fig — (A) The RNA-Seq reads from infected HEK-293 cells at 4 and 7 hpi with KOS or d27-1 in the presence of PAA or not were mapped to 44bp reference sequences of the genes listed. The reference sequences for gL, UL24, US11 and UL15 were taken from sequences immediate downstream of the 3’ss in order to represent coding sequences. The reference sequence for ICP22 (US1) was taken from the end of its coding sequences in order to distinguish ICP22 from ICP47, for which sequences near their splice sites are the same. The first 44 bp sequences following the start codon was used for the reference sequence for ICP4 as a control. All other reference sequences were taken from immediately upstream of the 5’ss of the genes. The expression level was normalized to the most abundant reads obtained among KOS and d27-1 infected cells. Results should be cautiously interpreted since some viral genes may share the same PAS. For example, although the US11 reference sequence was taken from its exon 2 coding region, ICP47 (US12) transcripts also share the same PAS. (B) The data presented in the panel (A) was replotted to show relative fold reduction. (TIF) [file ppat.1007884.s006.tif]
